# Ultrasound guidance in difficult radial artery puncture for blood gas analysis: A prospective, randomized controlled trial

**DOI:** 10.1371/journal.pone.0213683

**Published:** 2019-03-20

**Authors:** Romain Genre Grandpierre, Xavier Bobbia, Laurent Muller, Thibaut Markarian, Bob-Valéry Occéan, Stéphane Pommet, Claire Roger, Jean Yves Lefrant, Jean Emmanuel de la Coussaye, Pierre-Géraud Claret

**Affiliations:** 1 Department of Anesthesiology, Emergency and Critical Care Medicine, Intensive Care Unit, Nîmes University Hospital, Nîmes, France; 2 Emergency Department, Timone 2 Hospital, Aix-Marseille University, Marseille, France; 3 Department of Biostatistics, Nîmes University Hospital, Nîmes, France; University of Messina, ITALY

## Abstract

**Background:**

Ultrasound (US) guidance has yet to prove its applicability in radial arterial blood gas analysis (ABGA) punctures. The main objective of our study was to compare the number of first-attempt successes (NFAS) for radial arterial puncture in difficult patients with or without US guidance. The Secondary aims were to compare the number of punctures (NOP), puncture time, and patient pain.

**Methods:**

In this single-center, randomized controlled trial, patients who required a radial ABGA and in whom the arterial puncture was assessed as difficult (because of non-palpable radial arteries or two previous puncture failures by a nurse) were assigned to the US group or no-US (NUS) group (procedure performed by a trained physician).

**Results:**

Thirty-six patients were included in the US group and 37 in the NUS groups. The NFAS was 7 (19%) in the NUS group and 19 (53%) in the US group. The relative risk of success in the US group was 2.79 (95% CI,1.34 to 5.82), *p* = 0.01. In the NUS and US groups, respectively, the median NOP was 3 [2; 6] vs. 1 [1; 2], estimated difference −2.0 (95%CI, −3.4 to −0.6), *p* < 0.01; the respective puncture time was 3.1 [1.6; 5.4] vs. 1.4 [0.6; 3.1] min, estimated difference −1.45 (95%CI, −2.57 to −0.39), *p* = 0.01; the respective median patient pain was 6 [4; 8] vs. 2 [1; 4], estimated difference −4.0 (95%CI, −5.8 to −2.3); *p* < 0.01.

**Conclusion:**

US guidance by a trained physician significantly improves the rate of success in difficult radial ABGA patients.

## Introduction

Arterial blood gas analysis (ABGA) is routinely performed in emergency, critical care, and pulmonary departments principally to assess acid–base disturbances or to diagnose and quantify respiratory insufficiency. Although the number of inappropriate ABGAs is sometimes large [1], venous blood gas analysis cannot fully replace it for clinical decision-making [2]. Pain is the most frequent side effect [3].

In overcrowded emergency departments, difficult punctures for ABGA leads to increased workloads and wasted time. The number of failures at the first attempt is currently reported at around 10% [3, 4], and the procedure frequently results in multiple punctures. Moreover, repeated punctures of the radial artery have an important long-term effect on radial artery patency [5].

Ultrasound (US) guidance has demonstrated its benefit in various procedures [6–8]. This effect is less evident in techniques with a low rate of failure and complications such as peripheral intravenous access. In this case, US guidance seems to be a good tool for difficult cases [9]. In a first study, we showed than US guidance does not benefit arterial puncture success for ABGA in a general population of emergency patients [10]. This result was confirmed by Laursen et al. [4]. Our hypothesis was that US guidance would increase the number of first-attempt successes (NFAS) for radial arterial puncture in case of difficult cases.

The main aim of our study was to compare the NFAS for radial arterial puncture in difficult patients (defined as having non-palpable radial arteries or having had two previous nurse puncture failures) with or without US guidance. The secondary objectives were to compare the number of punctures (NOP), puncture time, patient pain, and physician satisfaction.

## Materials and methods

### Research involving human participants

The study protocol was approved by the *Comité de protection des personnes* (CCP Sud-Mediteranée III, ref. 2012.12.02 bis). This study was registered at clinicaltrials.gov (NCT01789801). All procedures were performed in accordance with the relevant guidelines and regulations. Written informed consent was obtained from all participants and/or their legal guardians.

### Study design and setting

This was a single-center, randomized controlled trial conducted in the emergency medicine department of a university hospital. An SAS (Carry,NC, USA) program was used to create random block sizes of 4 or 6 and to stratify the reason for inclusion as non-palpable artery and two failures by the nurse, with a ratio of 1:1. The subjects were enrolled between February 2014 and June 2016 and were followed until the ABGA was obtained.

### Selection of participants

Subjects were included who met all the following criteria: (a) provided written informed consent; (b) were affiliated or beneficiary of a health-insurance plan; (c) were aged 18 years or older; (d) had not previously been included in this study; (e) presented with a need for ABGA and at least one of the two following features: (i) non-palpable radial arteries, or (ii) two previous nurse puncture failures. The non-palpable radial artery was judged by a nurse and confirmed by a physician. We excluded subjects if they chose not to continue to participate in the study.

### Interventions

Subjects were prospectively enrolled when a US-trained doctor was available. Subjects who met the inclusion criteria were randomized at a ratio of 1:1 to either the US or the no-US (NUS) group. The randomization was done online at a dedicated website. When the emergency nurse received a medical prescription of ABGA and the patient met all the inclusion criteria (particularly non-palpable radial arteries or two failed attempts), the patient was randomized and the physician performed the radial puncture with or without US according to the randomization code. In the US group, US guidance was performed in real time with a conventional US device (Vivid S6, GE Healthcare, Medical System Israel Ltd., 4 Haetgar St. Tirat Carmel) and a vascular probe (Vivid 9L-RS, superficial probe 3.5–10.0 MHz). All physicians had a university degree in point-of-care US and all had previously used ABGA US guidance in clinical practice. After skin disinfection by a local antiseptic and the application of sterile gel at the puncture site, a timer was started when the probe touched the skin. Physicians could spot the radial artery by US using various modes (2B mode, color-flow mode, or pulse-wave mode) and centered it in the middle of the screen. A vascular probe was placed perpendicular to the artery. At this time, a 23 G needle was inserted at a 70° angle, with respect to the probe and aimed at the center of the arterial lumen. The physician controlled the needle’s position in real time, according to the short-axis approach [11, 12]. In the NUS group, after skin disinfection by a local antiseptic, the timer was started upon the first contact with the skin for palpation to find the radial artery. Once the artery was identified, the physician introduced a 23 G needle at 70°. The physician could choose which arm to use for the trial. A successful attempt was defined as blood return and the timer was stopped at that point. Success was confirmed when the ABGA results showed that the puncture was definitely arterial.

There were no changes to the methods after the commencement of the trial. The data were collected on an internal institutional computer site.

### Outcomes

The primary outcome was the number of successful punctures on the first attempt. One attempt corresponded to one break of the skin. The secondary outcomes were: (a) the number of attempts until successful puncture; (b) the elapsed time to successful puncture; (c) patient pain during the procedure; and (d) physician satisfaction. Patient pain and physician satisfaction were evaluated with a verbal numerical rating scale (VNRS) from 0 to 10 with 0 representing “no pain” and “not satisfied,” respectively. There were no changes to the trial outcomes after the trial began.

### Sample size

To the best of our knowledge, the success rate of difficult arterial punctures has not been previously reported. We considered the placement of radial arterial catheters as a comparable procedure. According to Shiver et al. [13], a 50% first-attempt puncture success rate could be expected in the NUS group and an 87% rate in the US group. To demonstrate a difference of 37% in the first-attempt puncture success rate between the two groups with a two-tailed alpha of 5% and a power of 90%, the number of subjects needed was 62 patients. In a conservative spirit, we increased this number by 20%, leading to a sample of 74 patients, with 37 patients per group.

### Analysis

The data were analyzed using SAS version 9 (SAS Institute, Cary, NC, USA), and expressed as numbers with percentages for categorical variables and as means with standard deviations (SD) or as median with 25^th^ and 75^th^ percentiles ([25^th^ percentile; 75^th^ percentile]) for quantitative variables according to their distribution. The normality of the distribution of the quantitative variables was explored using the Shapiro-Wilks normality test. The differences between the categorical and continuous variables were analyzed using the chi-squared test (or Fisher’s exact test) and Mann–Whitney U test (or Student’s *t*-test), respectively. Any randomized patient in the study was also included in the analysis on an intent-to-treat basis. Statistical significance was assumed at a *p*-value of < 0.05.

## Results

### Characteristics of study subjects

**Fig 1:**
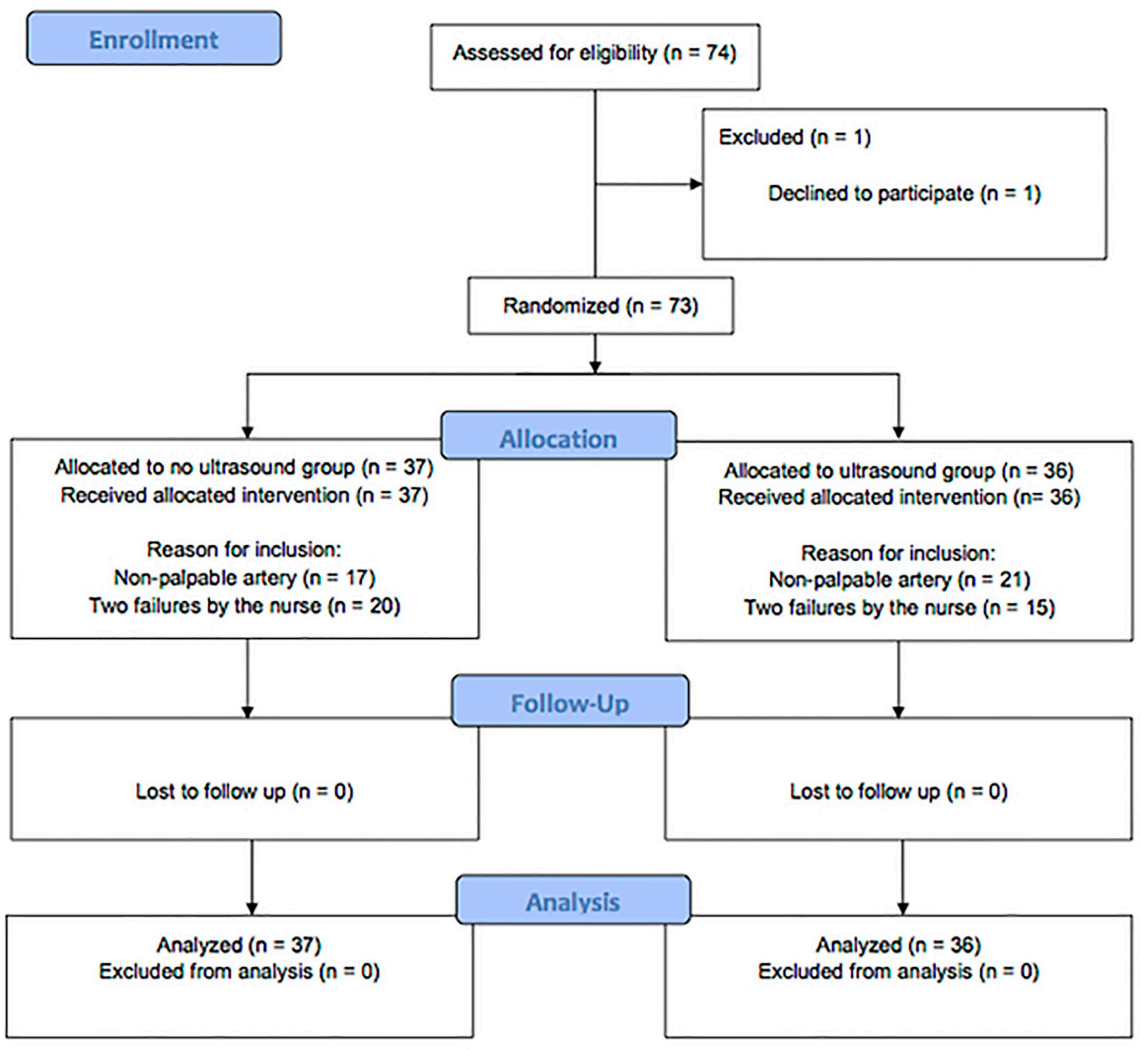
Flow chart.

From February 2014 through June 2016, a total of 74 patients were screened, and one was excluded prior to randomization (Fig 1). Seventy-three patients were randomized to either the US (*n* = 36) or the NUS group (*n* = 37). The trial ended when the number of subjects needed was reached. The baseline characteristics of the patients and physicians were similar in the two groups (Table 1). All outcomes were analyzed with 36 patients in the US group and 37 in the NUS group. Twenty-one emergency physicians (10 female, 48%) women participated in the study, with a US experience of 6 [4; 7] years.

**Table 1:** Characteristics of the population and reasons for blood gas analysis and for inclusion.

	No-ultrasound group (*n* = 37)	Ultrasound group (*n* = 36)
**Patient variables**		
Age (years)	73.3 (14.9)	73.3 (15)
Female gender, *n* (%)	23 (62)	23 (64)
History of arterial disease	11 (30)	7 (19)
Height (cm)	165 (6)	163 (11)
Weight (kg)	73 (18)	77 (22)
Systolic blood pressure (mmHg)	126 (34)	130 (31)
Diastolic blood pressure (mmHg)	71 (18)	70 (19)
**Indications of arterial blood gas analysis** ***n* (%)**		
Dyspnea	22 (59)	24 (67)
Suspicion of acid–base balance disruption	8 (22)	8 (22)
Suspicion of pulmonary embolism	5 (14)	5 (14)
**Reason for inclusion** ***n* (%)**		
Non-palpable artery	17 (46)	21 (58)
Two failures by a nurse	20 (54)	15 (42)

### Main results

The number of first-attempt successes was 7 (19%) in the NUS group and 19 (53%) in the US group. The relative risk of success in the US group was 2.79 (95% CI,1.34 to 5.82), *p* = 0.01. We had to perform US guidance for three patients to prevent one additional failure.

The median NOP after inclusion was 3 [2; 6] versus 1 [1; 2], respectively, in the NUS and US groups with an estimated difference of −2.0 (95% CI, −3.4 to −0.6), *p* < 0.01. The puncture time was 3.1 [1.6; 5.4] minutes in the NUS group versus 1.4 [0.6; 3.1] minutes in the US group with an estimated difference of −1.45 (95% CI, −2.57 to −0.39), *p* = 0.01. The median of patient pain and physician satisfaction for the NUS and US groups, respectively, were 6 [4; 8] versus 2 [1; 4] (estimated difference −4.0 (95% CI, −5.8 to −2.3), *p* < 0.01) and 4 [2; 8] versus 8 [6; 9] (estimated difference 4.0 (95% CI, 1.7 to 6.3], *p* = 0.01).

## Discussion

The main finding of this study is that US guidance increases every quality outcome related to difficult radial arterial punctures. If a low rate of radial arterial punctures is difficult (around 90% of first attempt success routinely [3, 4]), given the high prescription number, difficult arterial radial puncture is a daily problem in emergency department. Our study shows than when a nurse cannot palpate the radial artery or fails to puncture after two attempts, the use of US guidance decreases the risk of first-attempt failure by a factor of two. The success rate of difficult arterial punctures has not been previously reported. When we calculated the sample size, we assumed that a difficult arterial puncture was as difficult as an arterial catheterization. Our definition of difficult puncture (two failures or a non-palpable artery) seems to be associated with a higher failure rate. The problem of difficult radial arterial puncture is comparable to difficult peripheral venous access: As for radial arterial puncture, the majority of peripheral venous cannulations are easy. US guidance increases peripheral venous cannulation success in difficult cases [9, 14] but not among patients who are expected to have easy access [14]. For a slightly more difficult procedure, such as the catheterization of the radial artery, the benefit of the systematic US use is more obvious. In anesthesia, critical care and emergency medicine, US guidance decreases first-attempt failures, mean attempts to success, mean time to success, and overall complications of arterial radial catheterization [6, 15]. However, the benefit seems greater in more difficult conditions, such as shock or hypotension [16]. In cardiology, radial access has become the vascular access of choice in acute coronary syndrome patients undergoing invasive management [17]. US guidance improves the success and efficiency of radial artery cannulation [18]. A recent meta-analysis confirms these results and recommends routine use of US guidance for radial artery access [19].

The decrease in puncture time and in NOP reported in this study are also very interesting for emergency units. When US guidance is used, two fewer punctures are needed, and the ABGA is accomplished in two minutes sooner. These results can have a real impact in difficult conditions. First, all procedures must be done quickly in the case of acute-care patients [20, 21]. When a nurse or a physician recognizes the need for ABGA, the procedure can delay other urgent care. Second, in routinely overcrowded emergency departments, the time spent on testing has an effect on the unit’s overall efficiency [22]. The ABGA can also be considered as a moderately painful procedure [23]. In our study, US guidance significantly decreased the VNRS pain score from 6 to 2 points. This seems to be a consequence of the reduced NOP and puncture time. Thus, radial puncture is a painful procedure that can be ameliorated by US guidance. This is a significant finding, as previous studies have reported that reducing pain during ABGA, which is associated with the difficulty of the procedure [23], is difficult [24, 25]. Decreasing the puncture difficulty by using US is a valuable means of reducing the procedure’s pain. Moreover, repeated radial artery punctures have long-term consequences on arterial patency. The number of radial puncture attempts can predict pulsation loss and arterial occlusion at 30 days [5].

The present results raise several questions for future research. First, in our study, we performed US guidance with a short axis (out of plane). Two previous studies on radial ABGA US guidance also used this technique [4, 10]. In US-guided radial artery cannulation, short-axis and long-axis techniques have the same first-attempt cannulation success rate [26]. If ABGA is truly comparable to peripheral venous cannulation in term of US guidance, then an oblique approach could improve the first-attempt success rate and puncture time [27]. Optimization of the technique should thus be a research focus. Second, further research will also determine the performance of nurses with this technique. In difficult-to-access patients, nurses have more success with venous access when using US guidance [28]. Consequently, it would be better if the nurses became autonomous with regard to radial ABGA punctures, even when those are difficult. Finally, in this study, many physicians participated, improving external validity. These physicians were all experienced; several had participated in the first study [10] and have had continuous practice of radial ABGA US guidance since then. Nurses and paramedics are capable of a greater than a 70% success rate after the placement of only four peripheral intravenous catheters and the success rate increases to more than 88% after 15–26 attempts [29]. We hypothesize that the learning curve for radial ABGA US guidance would be no longer, but this requires specific studies. The development of a specific training regimen is also a necessity. A puncture simulator seems to be a good solution for training doctors and nurses in emergency departments.

### Limitations

The current study has several limitations. The trial was conducted at a single center and was not blinded. The open-label design of the study may have introduced a bias for the operator, particularly motivation bias. Indeed, the operators may have been convinced before the study of the value of the US in this application. The patients may also have been reassured by the use of the US device. The definition of difficult patients was dependent on the nurse and physician experience. Despite the participation of many physicians, the median experience was acceptable, but the results may have been poorer with beginners. We compared the puncture times without considering the total preparation time, and it is likely that this time will be longer when the US device needs to be prepared.

## Conclusions

In cases of difficult arterial radial puncture, US guidance by a trained physician significantly improves the first-attempt success rate. US guidance also reduces the puncture time and number of attempts and reduces procedure-related pain.

## Supporting information

S1 File Protocol English version. (PDF)

S2 File Protocol original version. Original protocol in French.
(PDF)

S3 File CONSORT checklist. (DOC)

S4 File Datasets used. (XLSX)
