# Supplementary material for: Ultrasound guidance in difficult radial artery puncture for blood gas analysis: A prospective, randomized controlled trial
Source: PLoS One. 2019 Mar 20;14(3):e0213683. doi: 10.1371/journal.pone.0213683 (PMC6426205; doi:10.1371/journal.pone.0213683)
Supplement: S1 File — (PDF) [file pone.0213683.s001.pdf]

ultrasound guidance Interest in "difficult" radial arterial blood puncture.

Dr. Romain GENRE GRANDPIERRE

Clinical research protocol

1 General informations

1.1 Identification

Intérêt de l'ECHOguidage dans la Ponction Artérielle sanguine Radiale « DIFficile ».

ECHO-PARDIF study

N° RCB: 2012-A01525-38
Code number : Local/2012/RGG-01
Version number and protocol date : Version N°11 - 7 mars 2016
Type of invitation to tender : Local 2012
Type of study : monocentric, pilot, comparative, controlled 2 parallel arm, randomized, open-label, controlled trial.
Source of funding : CHU de Nîmes
Project duration : 3 years
Total number of subjects required : 74

1.2 Main correspondents

Promoter :

CHU de Nîmes, Direction de la Recherche et de l'Innovation, Place du Professeur Debré, 30029 Nîmes Cedex 09 ; Tél : 04.66.68.42.36 ; Fax : 04.66.68.34.00 ; email : drc@chu-nimes.fr

Name and position of the principal investigator:

Dr Romain GENRE-GRANDPIERRE, Service des Urgences, DARDU, CHU de Nîmes, Place du Professeur Debré, 30029 Nîmes Cedex 09 ; Tél : 04.66.68.30.50 ; Fax : 04.66.6838.51 ; email : romainsbs@hotmail.com

Name and capacity of the person(s) authorized to sign the protocol and any amendments on behalf of the proponent:

Anissa Megzari, Director of Research, Partnerships and Medical Projects.
CHU de Nîmes, Direction, Place du Professeur Debré, 30029 Nîmes Cedex 09 ; Tél : 04.66.68.42.36 ; Fax : 04.66.68.34.00 ; email : drc@chu-nimes.fr

Methodologist :

Dr. Christophe Demattei (PhD), Service de Service de Biostatistique, Epidémiologie Clinique, Santé Publique et Information Médicale (BESPIM), CHU de Nîmes, Place du Professeur Debré, 30029 Nîmes Cedex 09 ; Tél : 04.66.68.35.53 ; Fax : 04.66.68.34.33 ; email : christophe.demattei@chu-nimes.fr

1.3 Approval of the protocol

Name Date and Signature of the Legal Representative of the Promoter:	Name Date and Signature of the Investigator Coordinator or Principal Investigator :
Le 10 mars 2016<br>La Directrice de la Recherche Clinique, des Partenariats, et des Projets Médicaux<br>Anissa MEGZARI	Le 10 mars 2016<br>Dr Romain GENRE GRANDPIERRE

1.4 History of protocol updates :

Version	Date	Raison de la mise à jour
1	26 Juillet 2012	Creation
2	30 Août 2012	CD proofreading and modifications
3	31 Août 2012	PR corrections
4	7 Septembre 2012	RGG corrections
5	7 Septembre 2012	Front version CS
6	24 Septembre 2012	Final version
7	21 Novembre 2012	CPP
8	12 décembre 2012	CD Corrections
9	5 mars 2012	Deferred notice response CPP
10	22 octobre 2014	MS 1
11	7 mars 2016	MS 2

1.5 List of investigators

Title. First name Last name	*Centre Adress*	*Téléphone - Fax - email*
**Dr. Romain GENRE-GRANDPIERRE**<br>***principal investigator***<br>*Qualification : PH*<br>*Specialty: Emergency Medicine*	CHU de Nîmes<br>Service des Urgences<br>Place du Professeur Debré<br>30029 Nîmes Cedex 09	Tél : 04 66 68 30 50<br>Fax : 04 66 68 38 51<br>romainsbs@hotmail.com
**Dr. Patrick Richard**<br>***Investigator***<br>*Qualification : PH*<br>*Specialty: Emergency Medicine*	CHU de Nîmes<br>Service Urgences-Réanimation<br>Place du Professeur Debré<br>30029 Nîmes Cedex 09	Tél : 04.66.68.36.28<br>Fax : 04.66.68.38.51<br>patrick.richard@chu-nimes.fr<br>N° RPPS : 10003196747
**Dr. Xavier BOBBIA**<br>***Investigator***<br>*Qualification : PH*<br>*Specialty: Emergency Medicine*	CHU de Nîmes<br>Service des Urgences<br>Pôle A.R.D.U.<br>Place du Professeur Debré<br>30029 Nîmes Cedex 09	Tél : 04 66 68 42 24<br>Fax : 04 66 68 38 51<br>xavier.bobbia@chu-nimes.fr
**Dr. Rémi PERRIN-BAYARD**<br>***Investigator***<br>*Qualification : PH*<br>*Specialty: Emergency Medicine*	CHU de Nîmes<br>Service des Urgences<br>Pôle A.R.D.U.<br>Place du Professeur Debré<br>30029 Nîmes Cedex 09	Tél : 06 08 32 68 70<br>Fax : 04 66 68 38 51<br>remspb@yahoo.fr
**Dr. Stéphane POMMET**<br>***Investigator***<br>*Qualification : PH*<br>*Specialty: Emergency Medicine*	CHRU de Nîmes<br>Services des Urgences-SAMU 30<br>Place du Professeur Debré<br>30029 Nîmes Cedex 09	Tél : 04.66.68.30.50<br>Fax : 04.66.68.38.51<br>stephane.pommet@free.fr<br>N° RPPS : 10100029379
**Dr. Alexandre MOREAU**<br>***Investigator***<br>*Qualification : PH*<br>*Specialty: Emergency Medicine*	CHRU de Nîmes<br>Services des Urgences-SAMU 30<br>Place du Professeur Debré	Tél : 04.66.68.30.50<br>Fax : 04.66.68.38.51<br>Duphalac69@yahoo.fr<br>N° RPPS : 1010097871

Title. First name Last name	*Centre Adress*	*Téléphone - Fax - email*
	30029 Nîmes Cedex 09	
**Dr. Pierre-Géraud CLARET**<br>***Investigator***<br>*Qualification : PH*<br>*Specialty: Emergency Medicine*	CHU de Nîmes<br>Service d'Accueil des Urgences<br>Pôle ARDU<br>Place du Professeur Debré<br>30029 Nîmes Cedex 09	Tél : 04 66 68 80 83<br>Fax : 04 66 68 38 51<br>pierre.geraud.claret@gmail.com<br>N° RPPS : 10003255907
**Dr. Yan BODIN**<br>***Investigator***<br>*Qualification : PH*<br>*Specialty: Emergency Medicine*	CHU de Nîmes<br>Service Urgences-Réanimation<br>Place du Professeur Debré<br>30029 Nîmes Cedex 09	Tél : 04.66.68.36.28<br>Fax : 04.66.68.38.51<br>yan.bodin@chu-nimes.fr<br>N° RPPS : 10100187318
**Dr. Emmanuelle GERVAIS**<br>***Investigator***<br>*Qualification : PH*<br>*Specialty: Emergency Medicine*	CHU de Nîmes<br>Service des Urgences<br>Pôle A.R.D.U.<br>Place du Professeur Debré<br>30029 Nîmes Cedex 09	Tél : 04 66 68 42 24<br>Fax : 04 66 68 38 51<br>emmanuelle.gervais@chu-nimes.fr<br>N° RPPS : 10100215424
**Dr. Aurélie COTTEL**<br>*Collaborator*<br>*Qualification : PH*<br>*Specialty: Emergency Medicine*	CHU de Nîmes<br>Service des Urgences<br>Pôle A.R.D.U.<br>Place du Professeur Debré<br>30029 Nîmes Cedex 09	Tél : 06 08 32 68 70<br>Fax : 04 66 68 38 51<br>aurelie.cottel@chu-nimes.fr<br>N° RPPS : 10100215390
**Dr. Nadine HANSEL**<br>*Collaborator*<br>*Qualification : PH*<br>*Specialty: Emergency Medicine*	CHRU de Nîmes<br>Services des Urgences-SAMU 30<br>Place du Professeur Debré<br>30029 Nîmes Cedex 09	Tél : 04.66.68.30.50<br>Fax : 04.66.68.38.51<br>nadine.hansel@chu-nimes.fr<br>N° RPPS : 10100029379
**Dr. Charlotte BOYARD**<br>*Collaborator*<br>*Qualification : PH*<br>*Specialty: Emergency Medicine*	CHRU de Nîmes<br>Services des Urgences-SAMU 30<br>Place du Professeur Debré<br>30029 Nîmes Cedex 09	Tél : 04.66.68.30.50<br>Fax : 04.66.68.38.51<br>Charlotte.boyard@chu-nimes.fr<br>N° RPPS : 10005181051

Title. First name Last name	*Centre Adress*	*Téléphone - Fax - email*
**Dr. Patricia WAGNER**<br>*Collaborator*<br>*Qualification : PH*<br>*Specialty: Emergency Medicine*	CHU de Nîmes<br>Service d'Accueil des Urgences<br>Pôle ARDU<br>Place du Professeur Debré<br>30029 Nîmes Cedex 09	Tél : 04 66 68 80 83<br>Fax : 04 66 68 38 51<br>patricia.wagner@ chu-nimes.fr<br>N° RPPS : 10003201083
**Dr. Aurélie DARDALHON**<br>*Collaborator*<br>*Qualification : PH*<br>*Specialty: Emergency Medicine*	CHU de Nîmes<br>Service des Urgences<br>Pôle A.R.D.U.<br>Place du Professeur Debré<br>30029 Nîmes Cedex 09	Tél : 04 66 68 42 24<br>Fax : 04 66 68 38 51<br>N° RPPS : 10003248951
**Dr. François HERNANDEZ**<br>*Collaborator*<br>*Qualification : PH*<br>*Specialty: Emergency Medicine*	CHU de Nîmes<br>Service des Urgences<br>Pôle A.R.D.U.<br>Place du Professeur Debré<br>30029 Nîmes Cedex 09	Tél : 06 08 32 68 70<br>Fax : 04 66 68 38 51<br>aurelie.cottel@chu-nimes.fr<br>N° RPPS : 10100215390
**Dr. Nadine HANSEL**<br>*Collaborator*<br>*Qualification : PH*<br>*Specialty: Emergency Medicine*	CHRU de Nîmes<br>Services des Urgences-SAMU 30<br>Place du Professeur Debré<br>30029 Nîmes Cedex 09	Tél : 04.66.68.30.50<br>Fax : 04.66.68.38.51<br>Francois.hernandez@chu-nimes.fr<br>N° RPPS : 10003199873
**Dr. Michael ROBERT**<br>*Collaborator*<br>*Qualification : PH*<br>*Specialty: Emergency Medicine*	CHRU de Nîmes<br>Services des Urgences-SAMU 30<br>Place du Professeur Debré<br>30029 Nîmes Cedex 09	Tél : 04.66.68.30.50<br>Fax : 04.66.68.38.51<br>michael.robert@chu-nimes.fr<br>N° RPPS : 10100165884
**Dr. Hélène ROBERT**<br>*Collaborator*<br>*Qualification : PH*<br>*Specialty: Emergency Medicine*	CHU de Nîmes<br>Service d'Accueil des Urgences<br>Pôle ARDU<br>Place du Professeur Debré<br>30029 Nîmes Cedex 09	Tél : 04 66 68 80 83<br>Fax : 04 66 68 38 51<br>Helene.robert@ chu-nimes.fr<br>N° RPPS : 10003256285

Title. First name Last name	*Centre Adress*	*Téléphone - Fax - email*
**Dr. Andrew STOWELL**<br>*Collaborator*<br>*Qualification : PH*<br>*Specialty: Emergency Medicine*	CHRU de Nîmes<br>Services des Urgences-SAMU 30<br>Place du Professeur Debré<br>30029 Nîmes Cedex 09	Tél : 04.66.68.30.50<br>Fax : 04.66.68.38.51<br>andrew.stowell@chu-nimes.fr<br>N° RPPS : 10100158053
**Dr. Jacques CRAMPAGNE**<br>*Collaborator*<br>*Qualification : PH*<br>*Specialty: Emergency Medicine*	CHRU de Nîmes<br>Services des Urgences-SAMU 30<br>Place du Professeur Debré<br>30029 Nîmes Cedex 09	Tél : 04.66.68.30.50<br>Fax : 04.66.68.38.51<br>jacques.crampagne@chu-nimes.fr<br>N° RPPS : 10003201117
**Dr. Jean FLECHET**<br>*Collaborator*<br>*Qualification : PH*<br>*Specialty: Emergency Medicine*	CHU de Nîmes<br>Service d'Accueil des Urgences<br>Pôle ARDU<br>Place du Professeur Debré<br>30029 Nîmes Cedex 09	Tél : 04 66 68 80 83<br>Fax : 04 66 68 38 51<br>Jean.flechet@ chu-nimes.fr<br>N° RPPS : 10003245510

1.1 Additional document

1. RCB number

2012-A01525-38

2. Full title of the research

Interest of ultrasound guidance in "difficult" radial arterial blood puncture.

3. Justification and critical analysis of the relevance of the research

Arterial blood gas puncture is a very frequent procedure in medical, intensive care or emergency room departments (8 to 12% of patients). The sampling technique was described in the nineteenth century and then updated. After disinfection with an antiseptic solution, it consists of identifying the artery by palpation followed by the introduction of a needle with a spontaneous flow of arterial blood into the syringe. The preferred route remains the radial route rather than the femoral or axillary routes because the artery is superficial, easily accessible and relatively far from adjacent veins.

However, this highly practiced procedure is not totally without risks. Even if complications remain rare (less than 1% for catheters), arterial puncture can cause vascular complications (pseudoaneurysm, hematoma), infection or nerve damage), but also bad experiences on the part of the patient following pain, a high number of attempts or failures.

Various solutions have been studied in order to try to reduce the bad feelings associated with the gesture, such as the use of anesthetic ointment such as EMLA, but without any significant results. The use of ultrasound imaging technique on difficult peripheral venous punctures has shown encouraging results in terms of number of attempts, duration of performance and number of complications. This experience could be applied to arterial puncture and cases were described as early as the mid-1970s.

However, there are very few randomized studies evaluating the value of ultrasound in the placement of arterial catheters; Levin et al (2003) in a study of 69 adults undergoing surgery, showed a 60% success rate at the first attempt using ultrasound compared to the 34% rate in the traditional palpation group. Out of 60 patients requiring randomized catheterization in the emergency room, Shiver et al (2006) showed a first trial success rate of 87% in the ultrasound group versus 50% in the palpation group. In a study of the catheterization of 30 children before neurosurgery, Schwemmer et al (2006) described a 100% success rate in the ultrasound group (with a 67% success rate at the first attempt) while it is only 80% in the palpation group.

However, other randomized studies describe more equivocal results. Thus, of 152 pediatric patients scheduled for surgery, Ganesh et al (2009) found that ultrasound did not facilitate faster radial arterial catheterization by inexperienced anesthesiologists in both palpation and ultrasound techniques; moreover, there was no difference between the two groups in terms of success rates, number of attempts or number of catheters used. (2003) did not find any benefit in using bi-directional Doppler in routine arterial catheterization in 166 patients undergoing general anesthesia.

In 2010, we carried out a preliminary study in order to validate ultrasound as a radial arterial blood sampling technique since we were able to show that it does not increase the estimate

of the pain felt during the puncture and that it gives satisfaction to patients. However, it increases the time required to perform the procedure, which is not negligible in emergencies in terms of medical time management and the quality of patient care. However, we had selected for this first study all patients presenting at the University Hospital of Nîmes UAS whose state of health required a blood arterial puncture for diagnostic, prognostic or therapeutic purposes.

In their study, Ganesh et al (2009) found that an experienced ultrasound operator was successful on many occasions in performing the ultrasound puncture while the initial operator had failed with the palpation technique.

These findings lead us to formulate the hypothesis that the use of ultrasound guidance could be validated as an alternative to the traditional technique in the case of punctures considered difficult. This is what we will seek to evaluate in this study, which will only include patients whose radial arteries are not palpable or who have had two puncture failures by the IDE.

4. Main research hypothesis and objectives

We hypothesize that the use of ultrasound guidance for difficult radial arterial punctures will improve the first attempt success rate, reduce the number of skin burns and immediate complications.

The main objective of this study is to determine whether performing a "difficult" radial arterial puncture (non-palpable artery or two consecutive failures by the IDE) with EPS under ultrasound improves the success rate at the first attempt. Failure is defined as insufficient blood collected, no blood flow or accidental venipuncture.

The secondary objectives of this study are to determine whether the use of an ultrasound scanner to perform a "difficult" SDS radial arterial puncture allows:

A. A decrease in the number of skin break-ins

B. A reduction in the duration of the project (min)

C. A decrease in immediate complications (hematoma, nerve damage, vagal reaction, pseudoaneurysm)

D. An improvement in the patient's experience of the procedure (EVA)

E. An improvement in the feeling of the nursing staff (EVA)

F. An improvement in patient pain (EVA)

G. A decrease in the number of catheters used

H. A decrease in the use of additional assistance

5. Assessment of the benefits and risks of the research, including the expected benefits for those who participate in the research and the foreseeable risks associated with the treatment and investigation procedures of the research (including, in particular, pain, discomfort, harm to the physical integrity of those who participate in the research, measures to avoid and/or manage unexpected events)

Ultrasound guidance could lead to an increase in the number of successful first attempts, as already observed in the case of difficult peripheral venous punctures, and therefore a decrease in the number of skin burglaries.

The risks associated with an arterial puncture with ultrasound guidance are the same as those associated with a puncture without ultrasound guidance: risk of skin breakdown, pain, haematomas, pseudo-aneurism, infection, nerve damage, vagal reaction.

6. Justification for the inclusion of the persons referred to in Articles L. 1121-5 to L. 1121-8 and L. 1122-1-2 of the Public Health Code (e. g. minors, protected adults, etc.) and the procedure implemented to inform and obtain the consent of these persons or their legal representatives

1. Justifications for the inclusion of persons referred to in Articles L. 1121-5 to L. 1121-8 and L. 1122-1-2 of the Public Health Code:

Minors, pregnant women, women who are pregnant, parturient, breastfeeding mothers, people in emergency situations as well as those under the protection of justice are not included in the study.

2. Procedures implemented to inform:

Patients will be recruited into the Emergency Department. When a patient meets the selection criteria, he/she will be informed by the investigator of the existence of the study. During this interview, the objectives as well as the constraints related to the conduct of the study and his rights will be explained to the patient who will be able to ask any questions he wishes.

Once this information has been provided and the investigator is satisfied that the patient understands the implications of his or her participation in the study, the patient will be free to accept or refuse to participate in the study from which he or she may withdraw at any time and for any reason, and without incurring any liability. Whatever the reason, refusal to participate will have no impact on the quality of care that the investigator will continue to provide or on the quality of the physician-patient relationship.

In accordance with article R.5120 of the Public Health Code, investigators and all persons called upon to collaborate in the study are bound by professional secrecy, in particular with regard to the nature of the study, the persons who participate in it and the results obtained. The investigator will ensure that the anonymity of patients is respected. On all documents, patients will be identified by a numerical code.

The subject's signature of free and informed consent will be collected during the inclusion visit. The patient will be given a few minutes to reflect between the presentation of the information note and the signing of the consent.

7. Description of how to recruit people

It is a monocentric, pilot, comparative, controlled trial with 2 parallel arms, randomized, open-label.

Patients will be recruited upon admission to the Emergency Department.

Criteria for inclusion of patients: the patient must have given free and informed consent and signed the consent, must be affiliated or beneficiary of a health insurance scheme, must be at least 18 years

old. The patient's condition must require a radial arterial blood puncture and the patient's radial arteries are not palpable or there have been two prior puncture failures by the IDE.

Criteria for non-inclusion of patients: the subject participates in another study, is in an exclusion period determined by a previous study, is under the protection of justice, under guardianship or curatorship. The subject refuses to sign the consent, it is impossible to give the subject informed information. The patient is pregnant, parturient, or breastfeeding. The puncture must be performed on the femoral, humeral or axillary artery. The patient is in cardiorespiratory arrest, has known circulatory disorders, ischemia, local infection. The patient has coagulation disorders or severe hemostasis (hemophilia, hypoprothrombinemia, platelets < 50,000). The patient is amputated with an upper limb or has his arm in a cast. The patient has known allergies to methyl or propylbenzoate, propylene glycol or chlorxhidine gluconate. The patient has arteriovenous fistula or arteriovenous malformation.

8. Investigation procedures conducted and differences from standard care

Only one inclusion visit/research completion visit is required to complete this study.

Following the inclusion of a patient, a randomization is made to assign him/her either to the reference management group ("palpation") or to the intervention management group ("ultrasound guidance"). The comparison of these two groups provides the methodological framework for this research.

The pain experienced, patient and caregiver satisfaction will be assessed using an EVA.

Apart from performing an ultrasound scan in the interventional arm, the patient's management is unchanged compared to normal medical practice. No specific biological examination is associated with this study.

The inclusion period will be 30 months. The total duration of the trial will be 3 years.

9. Justification of the existence or not of: (1) a prohibition to participate simultaneously in other research; (2) an exclusion period during which participation in other research is prohibited.

Patients who participated in a study in the 3 months prior to their inclusion in this protocol are excluded in order to eliminate any interference (known or unknown) that could lead to over- or under-estimation of potential changes in the assessment of assessment criteria during the study and thus distort the conclusions. Included patients are prohibited from simultaneously participating in another intervention research for the same reasons.
There will be no exclusion period at the end of the trial.

10. Terms and amount of compensation for research subjects

There are no plans to compensate individuals for research.

11. Reasons for setting up or not an independent supervisory committee

There are no plans to establish an independent oversight committee.

12. Expected number of people to be included in the research

74 or 37 per arm

1.2 Executive Summary

1 General information ________ *Erreur ! Signet non défini.*
1.1 Identification ________ Erreur ! Signet non défini.
1.2 Main correspondents ________ Erreur ! Signet non défini.
1.3 Approval of the protocol ________ Erreur ! Signet non défini.
1.4 History of protocol updates ________ Erreur ! Signet non défini.
1.5 List of investigators ________ Erreur ! Signet non défini.
1.6 List of abbreviations and definitions ________ Erreur ! Signet non défini.
1.7 Additional document ________ Erreur ! Signet non défini.
1.8 Executive Summary ________ **5**
2 Scientific justification and general description of the research ________ *10*
2.1 Current state of knowledge ________ **10**
2.1.1 Radial arterial puncture ________ 10
2.1.2 Research hypotheses ________ 12
2.1.3 Originality and expected impact of the study ________ 12
2.2 Summary of foreseeable and known benefits and risks for research subjects ________ **12**
2.2.1 Benefits ________ 12
2.2.2 Risks ________ 12
2.3 Statement that the research will be conducted in accordance with the protocol, good practices and legislative and regulatory provisions in force ________ **12**
2.4 References ________ **12**
3 Research objectives ________ *12*
3.1 Main objective ________ **13**
3.2 Secondary objectives ________ **13**
4 Research design ________ *13*
4.1 Methodology and methodology ________ **13**
4.1.1.1 Type of test and characteristics ________ 13
4.1.2 Evaluation criteria ________ 15
4.1.3 Cofactors ________ 15
4.2 Planned visits and reviews ________ **15**
4.2.1 Pre-inclusion visit ________ 16
4.3 Description of the evaluation parameters and methods for measuring, collecting and analysing these parameters ________ **18**
4.3.1 Clinical observations ________ 18
4.3.2 Visual analogue scales ________ 18
4.3.3 Arterial punctures ________ 18
4.3.4 Criteria extracted from the database ________ 19

4.4 Identification of all data to be collected directly in the observation notebooks 19

5 Selection and exclusion of persons from research 19

5.1 Description of the population to be studied 19

5.2 Criteria for including people who are suitable for research 19

5.3 Criteria for not including people who are suitable for research 20

5.4 Expected duration of participation of individuals, and description of the chronology and duration of all research periods, including follow-up 20

5.4.1 Expected duration of a subject's participation 20

5.4.2 Expected duration of the research 20

5.4.3 End of the research 21

5.5 Description of the rules for permanent or temporary cessation 21

5.5.1 An individual's participation in research 21

5.5.2 Some or all of the research 22

5.6 Procedures for monitoring persons 22

6 Treatment used in research subjects 23

6.1 Description of any medical device or in vitro diagnostic medical device or drug that is used for research purposes including the follow-up period 23

6.2 Drugs and treatments authorised and prohibited under the Protocol, including rescue drugs 23

7 Feasibility 23

7.1 Patient management 23

7.1.1 Expected number of people to be included in the research 23

7.1.2 Patient Recruitment 24

7.1.3 Patient circuit 24

7.1.4 Patient transport 24

7.1.5 Additional information for patients 25

7.2 Medical-technical management 25

7.2.1 Pharmaceutical circuit 25

7.2.2 Biological analyses 25

7.2.3 Imaging 25

7.3 Randomization and blinding 25

7.3.1 The draw 25

7.3.2 Blinding methods 26

7.3.3 Provisions implemented to maintain blinding and procedures for removing blinding 26

7.4 Personnel management 26

7.4.1 Training to be planned 26

7.4.2 Required ARC/TEC time ____ 26

7.4.3 Meetings to be scheduled ____ 27

8 Security assessment ____ 27

8.1 Description of safety assessment parameters ____ 27

8.2 Planned methods and schedule for measuring, collecting and analyzing security assessment parameters ____ 27

8.3 Procedures in place for recording and reporting adverse events ____ 27

8.3.1 Reporting Responsibilities ____ 27

8.3.2 Definitions ____ 27

8.3.3 Description of Expected Adverse Events ____ 28

8.3.4 Action to be taken in the event of an adverse event or new fact ____ 28

8.4 Methods and duration of follow-up of individuals following the occurrence of adverse events 29

8.5 Oversight Committee ____ 29

9 Statistics and statistics ____ 29

9.1 Description of the planned statistical methods, including the timetable for the planned interim analyses ____ 29

9.1.1 Description of the included population and the main parameters studied ____ 30

9.1.2 Statistical analyses ____ 30

9.2 Expected level of significance ____ 30

9.3 Statistical criteria for stopping the search ____ 30

9.4 Method of taking into account missing, unused or invalid data; ____ 31

9.5 Choice of persons to be included in the analyses ____ 31

9.6 Management of changes to the initial statistical plan ____ 31

10 Right of access to source data and documents ____ 31

10.1 Right of access of the promoter and the competent authority to source data and documents ____ 31

10.2 Mention of the submission to the CNIL ____ 31

11 Quality control and assurance ____ 31

11.1 Monitoring & quality control ____ 31

11.2 Audit and inspection ____ 32

12 Ethical considerations ____ 32

12.1 Duty of the investigator ____ 33

12.1.1 The possibility for the investigator to reserve certain information ____ 33

12.2 Proponent's obligation ____ 33

12.2.1 Amendment to the Protocol ____ 33

12.3 Justification for the collection of ethnic or racial information, if applicable. _________ 34
13 Data processing and storage of documents and research data _______________ 34
13.1 Data collection and management ____ 34
13.2 Data Entry and Data Management Procedures__________________________________ 34
13.3 Conservation and Archiving ____ 35
14 Financing and insurance ____ 36
14.1 Duration of the insurance coverage and specific terms and conditions for covering risks beyond 36
14.2 Acts and processing specific to research ______________________________________ 36
14.3 Total amount needed for research ____ 36
14.4 Sources of funding ____ 36
15 Rules on publication ____ 36
15.1 Study Report : ____ 36
15.2 Publication Rules : ____ 36
15.2.1 Mention of the University Hospital of Nîmes, Nîmes, France _______________________36
15.2.2 Articles and Conferences ____37
15.2.3 Press Releases and Reactive Nîmes ____37
15.3 Communication of results to patients ____ 37
15.4 Data transfer____ 37
16 Appendices____ 38
16.1 Patient information letter ____ 38
16.2 Free and Informed Consent Form for the patient participating in biomedical research _ 42
16.3 Visual Analogue Scale (VAS)____ 45
16.4 References ____ 46

2 Scientific justification and general description of the research

2.1 Current state of knowledge

2.1.1 Radial arterial puncture

Arterial blood gas puncture is a very frequent procedure in medical services (Sarrot-Reynauld et Paramelle 2003), intensive care or emergency room (8 to 12% of patients).

The study of blood gases is an established aid in the diagnosis and monitoring of many pathologies such as tromboembolic diseases (Lorut 2005), respiratory pathologies in decompensation (Chabot et al. 2009; ,Taytard et al. 2001)patients ventilated in pre-hospitalTaytard et al. 2001)A and in hospital (Maggiore et Jolliet 2005), disturbances of the acid-base balance or carbon monoxide intoxications (Donati, Gainnier, et Chibane-Donati 2005).

The sampling technique was described in the nineteenth century and then updated (Marshall 1964). After disinfection with an antiseptic solution, it consists of identifying the artery by palpation followed by the introduction of a needle with a spontaneous influx of arterial blood into the syringe (Franklin 1995). The preferred pathway remains the radial pathway rather than the femoral or axillary pathways (Martin 1995) because the artery is superficial, easily accessible and relatively distant from adjacent veins.

However, this highly practiced procedure is not totally without risks. Even if complications remain rare (less than 1% for catheters (Scheer, Perel, et Pfeiffer 2002)), arterial puncture can cause vascular complications (pseudoaneurysm, hematoma), infection or nerve damage (Sarrot-Reynauld et Paramelle 2003; Spunda et al. 2005)), but also bad experience on the part of the patient due to pain, a high number of attempts or failures (Sarrot-Reynauld et Paramelle 2003).

Various solutions have been studied in an attempt to reduce the bad feelings associated with the gesture, such as the use of anesthetic ointment of the EMLA type (Giner et al. 2000; Micu et al. 2006), but without any significant results.

The use of ultrasound imaging technique on difficult peripheral venous punctures (Costantino et al. 2005; Bauman, Braude, et Crandall 2009) has shown encouraging results in terms of number of attempts, duration of performance and number of complications. This experience could be applied to arterial puncture and cases were described as early as the mid-1970s (Nagabhushan et al. 1976). Studies show that in addition to standard arterial catheterization for monitoring purposes, ultrasound has also been used to guide interventional procedures (Shiloh and Eisen 2010). Some case reports have identified new uses for ultrasound guidance in arterial cathetherization. Kannan (2005) describes a case of brachial artery cathetherization by ultrasound assistance in intensive care units in a patient with significant edema in whom conventional tests were unsuccessful. Sandhu and Patel (2005) reported a few cases where the use of ultrasound could be a backup alternative in the event of failure of radial artery cannulations because it overcomes obstacles such as oedema, vasospasm or hematoma. Wilson et al (2010) report a case where ultrasound is validated in the emergency room as a tool to guide the cathetherization of the radial artery in a patient who is unable to cooperate in the traditional Allen's test.

However, there are very few randomized studies evaluating the value of ultrasound in the placement of arterial catheters (Shiloh and Eisen 2010); Levin et al (2003) in a study of 69 adults undergoing surgery, demonstrated a 60% success rate at first attempt using ultrasound compared to the 34% rate in the traditional palpation group. Out of 60 patients requiring randomized catheterization in the emergency room, Shiver et al (2006) showed a first trial success rate of 87% in the ultrasound group versus 50% in the palpation group.

In a study of the catheterization of 30 children before neurosurgery, Schwemmer et al (2006) described a 100% success rate in the ultrasound group (with a 67% success rate at the first attempt) while it is only 80% in the palpation group. One of the main reasons for the difficulty of catheterization in children is of course the small section of the radial artery making palpation difficult. Schwemmer et al (2006) describe a section of the radial artery between 1 and 4.2 mm2, this area being reduced by 19% by a dorsiflexion of the wrist. Mizukoshi et al (2009) evaluated the optimal wrist positioning by ultrasound guidance during radial artery cannulation in 17 healthy patients and 17 patients requiring coronary artery bypass surgery (PAC); radial artery dimensions were measured at wrist angles of 0°, 15°, 30°, 45°, 60° and 75°. In healthy patients, the dimensions of the radial artery do not change when the wrist joint is increased to an angle of 45°. However, an extension to 60° in healthy patients and 75° in PAC patients results in a reduction in the height of the radial artery, which could make arterial catheterization more difficult.

In these first three studies, a significantly lower number of attempts and catheters were required for positioning catheters in the radial artery under ultrasound. Also, radial arterial puncture under ultrasound required significantly less time than more traditional techniques. However, other randomized studies describe more equivocal results. Thus, of 152 pediatric patients scheduled for surgery, Ganesh et al (2009) found that ultrasound did not facilitate faster radial arterial catheterization by inexperienced anesthesiologists in both palpation and ultrasound techniques; moreover, there was no difference between the two groups in terms of success rates, number of attempts or number of catheters used. (2003) did not find any benefit in using bi-directional Doppler in routine arterial catheterization in 166 patients undergoing general anesthesia. Finally, in 2004, Dudeck et al. in the case of femoral artery puncture in interventional investigations in 112 patients concluded that ultrasound should be used in patients with a weak arterial pulse or leg circumference greater than or equal to 60 cm; in these subjects, there was a decrease in the number of attempts and a decrease in the time required for puncture compared to traditional palpation technique.

In 2010, we carried out a preliminary study in order to validate ultrasound as a radial arterial blood sampling technique since we were able to show that it does not increase the estimate of the pain felt during the puncture and that it gives satisfaction to patients. However, it increases the time required to perform the procedure, which is not negligible in emergencies in terms of medical time management and the quality of patient care. However, we had selected for this first study all patients presenting at the University Hospital of Nîmes UAS whose state of health required a blood arterial puncture for diagnostic, prognostic or therapeutic purposes.

In their study, Ganesh et al (2009) found that an experienced ultrasound operator was successful on many occasions in performing the ultrasound puncture while the initial operator had failed with the palpation technique.

These findings lead us to formulate the hypothesis that the use of ultrasound guidance could be validated as an alternative to the traditional technique in the case of punctures considered difficult. This is what we will seek to evaluate in this study, which will only include patients whose radial arteries are not palpable or who have had two puncture failures by the IDE.

2.1.2 Research hypotheses

We hypothesize that the use of ultrasound guidance for difficult radial arterial punctures will improve the first attempt success rate, reduce the number of skin burns and immediate complications.

2.1.3 Originality and expected impact of the study

The expected outcomes of this study are a change in professional practices during arterial blood gas punctures.

2.2 Summary of foreseeable and known benefits and risks for research subjects

2.2.1 Benefits

Ultrasound guidance could lead to an increase in the number of successful first attempts, as already observed in the case of difficult peripheral venous punctures, and therefore a decrease in the number of skin burglaries.

2.2.2 Risks and risks

The risks associated with an arterial puncture with ultrasound guidance are the same as those associated with a puncture without ultrasound guidance: risk of skin breakdown, pain, haematomas, pseudo-aneurism, infection, nerve damage, vagal reaction.

2.3 Statement that the research will be conducted in accordance with the protocol, good practices and legislative and regulatory provisions in force

The investigator undertakes to work in accordance with Good Clinical Practices defined by the decision of 24 November 2006 by the Ministry of Health and Solidarity, and in accordance with the regulations in force (Public Health Code).

The investigator undertakes, for himself and for all persons involved in monitoring the conduct of the study, to guarantee the confidentiality of all information relating to the project until the publication of the trial results. This confidentiality obligation shall not apply to information that the investigator may disclose to patients in the course of their participation in the trial or to information already published.

2.4 References

See Annex 16.4 at the end of the protocol.

3 Research objectives

3.1 Main objective

The main objective of this study is to determine whether performing a "difficult" radial arterial puncture (non-palpable artery or two consecutive failures by the IDE) with EPS under ultrasound improves the success rate at the first attempt. Failure is defined as insufficient blood collected, no blood flow or accidental venipuncture.

3.2 Secondary objectives

The secondary objectives of this study are to determine whether performing a "difficult" radial arterial puncture (non-palpable artery or two consecutive failures by the IDE) with ultrasound to SDS allows:

A- A decrease in the number of skin break-ins

B- A reduction in the duration of the project (min)

C- A decrease in immediate complications (hematoma, nerve damage, vagal reaction, pseudoaneurysm)

D- An improvement in the patient's experience of the procedure (EVA)

E- An improvement in the feeling of the nursing staff (EVA)

F- An improvement in patient pain (EVA)

G- A decrease in the number of catheters used

H- A decrease in the use of additional assistance

4 Research design

4.1 Methodology and methodology

4.1.1 Type of test and characteristics

It is a monocentric, pilot, comparative, controlled trial with 2 parallel arms, randomized, open-label.

Following the inclusion of a patient, a randomization is made to assign him/her either to the reference management group ("palpation") or to the intervention management group ("ultrasound guidance"). The comparison of these two groups provides the methodological framework for this research (Figure 4.1).

The caregiver who will perform the difficult radial arterial puncture should be designated before randomization (to avoid bias due to a lack of control in the use of ultrasound guidance) which implies that only caregivers who are able to perform the puncture with both techniques (palpation and ultrasound guidance) can perform the puncture.

All physicians participating in the study have validated a diploma in ultrasound and are trained in the practical performance of this type of puncture (at least 20 half-days); a training on

"ghosts" (synthetic silicone gel prosthesis used for training and gesture training) will also be conducted for the study.

If the puncture related to the test is performed after two unsuccessful attempts of the IDE, it shall be performed on the same side with a rest period of 15 minutes.

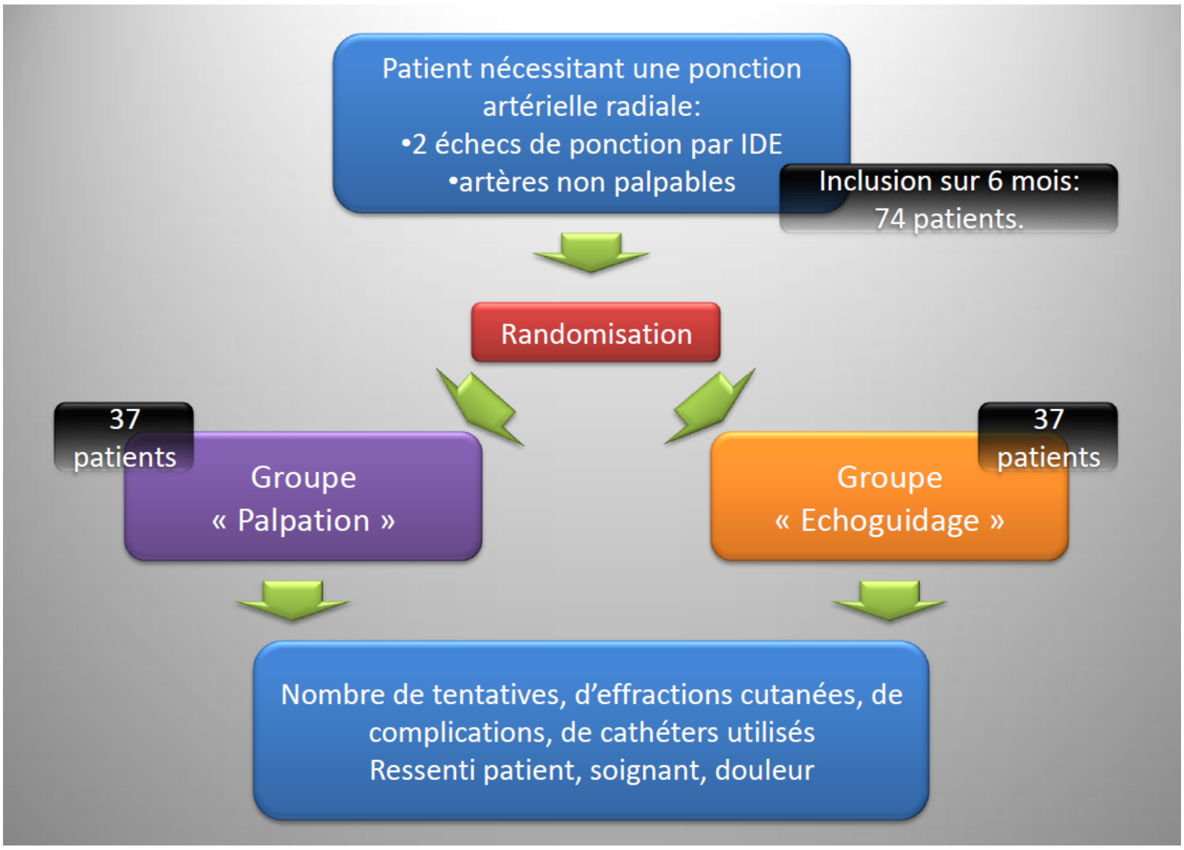

Figure 4.1: Study design.

4.1.2 Evaluation criteria

Table 4-1: Evaluation criteria, as well as associated units, time horizons and objectives.

Evaluation Criteria	Units	Time Horizon	Objective
Number of skin break-ins	N	J0	A
Duration of realization	min	J0	B
**Only one attempt is necessary**	**Y/N**	**J0**	**Principal**
Complications (hematoma, nerve damage, vagal reaction, pseudo-aneurism)	Y/N	J0	C
Patient Satisfaction (EVA)	0-10	J0	D
Caregiver Satisfaction (VAS)	0-10	J0	E
Patient pain assessment (EVA)	0-10	J0	F
Number of catheters used	N	J0	G
Use of additional assistance	Y/N	J0	H

4.1.3 Co-factors

Age (year)

Gender (F/M)

Patient with an artery that is not palpable/patient who has had two puncture failures by the IDE

Medical indication for arterial puncture

Systolic blood pressure

Underlying vascular disease: Y/N

Function of the caregiver performing the puncture

Level of expertise in ultrasound: DIUE, DU

4.2 Planned visits and reviews

Only one inclusion/end of research visit (Vinc/Vfin) is required to conduct this study.

Table 4-2 The planned visits, their chronology, and the associated procedures, examinations and samples.

	Vinc/Vfin
Timeline (days, weeks, months, years)	J0
Acts, examinations, samples:	
Consultation	✖

General information	✖
Presentation of the information note	✖
Validation of inclusion and non-inclusion criteria	✖
Collection of informed consent	✖
Randomization	✖
Ultrasound (in the Echoguidance group)	✖*
Pain Assessment (EVA)	✖*
Patient Satisfaction Assessment (EVA)	✖*
Caregiver Satisfaction Assessment (CSA)	✖*
Complications report	✖*
Collection of adverse events	✖

*These examinations are not part of the patient's usual management and are specific to research.

4.2.1 Inclusion visit

During this visit the investigator:

- Provide oral and written information (delivery of the information note; see Annex 16.1 at the end of the protocol) on the implementation of the study, its objectives, its constraints and the rights of the patient.
- Collect informed consent (see Annex 16.2 at the end of the protocol)
- Once the consent is signed, the subject will be randomized. This randomization is accessible via a WEB application made available by the Biostatistics, Clinical Epidemiology, Public Health, & Medical Information (BESPIM) department of the University Hospital of Nîmes.
- Perform radial arterial puncture with or without ultrasound depending on the randomization arm
- After puncture, assess the patient's pain (EVA)
- Assess patient satisfaction (EVA)
- Evaluate caregiver satisfaction (VAS)

4.2.1.1 Procedures implemented to inform, and signature of the subject's free and informed consent before any study procedure:

4.2.1.1.1 Procedures implemented to inform adults

Patients will be recruited into the Emergency Department. When a patient meets the selection criteria, he/she will be informed by the investigator of the existence of the study. During this

interview, the objectives as well as the constraints related to the conduct of the study and his rights will be explained to the patient who will be able to ask any questions he wishes.

Once this information has been provided and the investigator is satisfied that the patient understands the implications of his or her participation in the study, the patient will be free to accept or refuse to participate in the study from which he or she may withdraw at any time and for any reason, and without incurring any liability. Whatever the reason, refusal to participate will have no impact on the quality of care that the investigator will continue to provide or on the quality of the physician-patient relationship.

In accordance with article R.5120 of the Public Health Code, investigators and all persons called upon to collaborate in the study are bound by professional secrecy, in particular with regard to the nature of the study, the persons who participate in it and the results obtained. The investigator will ensure that the anonymity of patients is respected. On all documents, patients will be identified by a numerical code.

The subject's signature of free and informed consent will be collected during the inclusion visit. The patient will be given a few minutes to reflect between the presentation of the information note and the signing of the consent.

4.2.1.1.2 Procedures implemented to inform minors and their parents/legal representatives

Minors are excluded from this search. There are therefore no specific provisions for the inclusion of these persons with regard to free and informed consent.

4.2.1.1.3 Procedures implemented to inform patients in an emergency situation

There will be no emergency situation, as this is a scheduled response. There are therefore no specific provisions for emergency situations regarding free and informed consent.

4.2.1.1.4 Procedures implemented to inform patients under guardianship or curatorship and their trusted persons/legal guardians

Persons under guardianship or curatorship are excluded from this research. There are therefore no specific provisions for the inclusion of these persons with regard to free and informed consent.

4.2.1.1.5 Procedures implemented to inform patients under the protection of justice

Persons under the protection of justice are excluded from this research. There are therefore no specific provisions for the inclusion of these persons with regard to free and informed consent.

4.2.1.2 The prohibition for persons to participate simultaneously in another research and the exclusion period provided for at the end of the research

Patients who participated in a study in the 3 months prior to their inclusion in this protocol are excluded in order to eliminate any interference (known or unknown) that could lead to over- or under-estimation of potential changes in the assessment of assessment criteria during the study and thus

distort the conclusions. Included patients are prohibited from simultaneously participating in another intervention research for the same reasons.

There will be no exclusion period at the end of the trial.

4.2.1.3 Compensation of participants

There will be no compensation for patients participating in the study.

4.3 Description of the evaluation parameters and methods for measuring, collecting and analysing these parameters

4.3.1 Clinical observations

Clinical observations will be recorded in the observation booklet as the study progresses.

4.3.2 Visual analogue scales

The EVA scale (see section 16.3 at the end of the protocol) will be presented to patients, who will mark the level of pain experienced during the assessment directly on the scale. An identical scale will be used to assess the patient's or caregiver's satisfaction with the procedure.

4.3.3 Arterial punctures

If the puncture related to the test is performed after two unsuccessful attempts of the IDE, it shall be performed on the same side with a rest period of 15 minutes.

4.3.3.1 Non-echo-guided arterial puncture known as standard ("palpation" group)

The so-called standard arterial puncture is performed according to the algorithm defined as follows:

- Skin disinfection with local antiseptic
- Identification of the radial artery by palpation
- Introduction of the needle positioned at 70° opposite the palpated artery
- Check for arterial blood build-up in the syringe.

To measure the duration of the procedure, the stopwatch will be activated as soon as the artery is first palpated and stopped as soon as blood rises in the syringe.

The sampling will only be carried out by tea doctors, usual practitioners of the Emergency Department.

4.3.3.2 Echo-guided arterial puncture

Arterial puncture with ultrasound guidance is performed as follows:

- Skin disinfection with local antiseptic
- Application of sterile gel type K-Y (ETHICON SAS)
- Identification of the artery by 9L vascular probe on ***Philips Vivid S6*** device

-Centering the artery in the center of the screen

-Insert the syringe at 70° opposite the probe, right in the middle of it

- Check for arterial blood build-up in the syringe.

To measure the duration of the procedure, the stopwatch will be activated from the first disinfection step and stopped as soon as blood rises in the syringe.

4.3.4 The criteria extracted from the database

Composite criteria based on clinical observations do not necessarily appear in the observation book, but are extracted from the final database. For example, the patient's weight and height will be recorded in the observation book, but the BMI (= kg/m2) will be calculated using computers.

4.4 Identification of all data to be collected directly in the observation notebooks

The data to be collected directly in the observation books includes the minimum data necessary for analysis:

- the judgment criteria described in section 4.1.2
- the cofactors described in section 4.1.3
- the variables described in the visit descriptions (section 4.2.1)
- the inclusion and non-inclusion criteria described in Section 5
- complications and adverse events related to the study

5 Selection and exclusion of persons from research

5.1 Description of the population to be studied

The study population consists of patients presenting to the Emergency Department (SAU) of the University Hospital of Nîmes and requiring a radial arterial puncture for diagnostic, prognostic or therapeutic purposes.

5.2 Criteria for including people who are suitable for research

General inclusion criteria :

- The patient must have given free and informed consent and signed the consent
- The patient must be a member or beneficiary of a health insurance plan
- Women and men are included
- The patient is at least 18 years old

Inclusion criteria for the target population:

- The patient's condition requires a radial arterial blood puncture AND the patient's radial arteries are non-palpable or there have been two previous puncture failures by the IDE.

5.3 Criteria for not including people who are suitable for research

General non-inclusion criteria :

- The subject is participating in another study
- The subject is in an exclusion period determined by a previous study
- The subject is under the protection of justice, guardianship or curatorship
- Subject refuses to sign consent
- It is impossible to give informed information about

Criteria for non-inclusion regarding interfering diseases or associated conditions:

- The patient is pregnant, parturient, or breastfeeding
- The puncture must be performed on the femoral, humeral or axillary artery.
- The patient is in cardiorespiratory arrest
- The patient has known circulatory disorders, ischemia, local infection
- The patient has coagulation or severe hemostasis disorders (hemophilia, hypoprothrombinemia, platelets < 50,000)
- The patient is amputated with an upper limb or has his arm in a cast.
- The patient has arteriovenous fistula or arteriovenous malformation.
- The patient has known allergies to methyl or propylbenzoate, propylene glycol or chlorxhidine gluconate.

5.4 Expected duration of participation of individuals, and description of the chronology and duration of all research periods, including follow-up

5.4.1 Expected duration of participation of a subject

The subject's participation in the study consists of a single visit of no more than 2 hours.

5.4.2 Expected duration of the search

The study's provisional schedule includes 30 months of inclusion, 6 months of data management, statistical analysis and writing of the study report (see Figure 4-2).

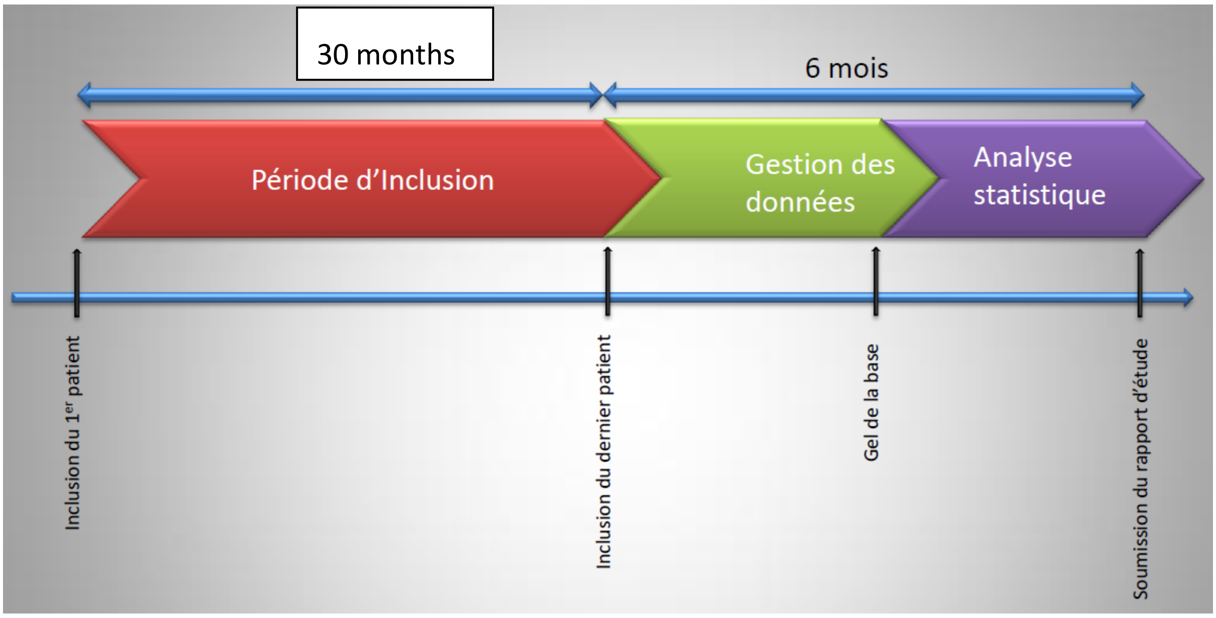

Figure 4.2: Diagram of the different phases of the study.

Table 5-1: Forecast Schedule

Milestone	Date
CPP, ANSM requests	April 2013
Beginning of inclusions	February 2014
End of inclusions	August 2015
End of research + end of statistical analysis and report writing	February 2016

5.4.3 End of the search

The end of the clinical part of this research corresponds to the date of the last visit of the last person who is suitable for the research.

The trial end form will be completed for any patient who has normally completed the study or who has left early.

After the end of the trial, the usual follow-up will be carried out by the investigator, who will decide on the frequency of follow-up visits.

The end of the search corresponds to the date of the database freeze (see Figure 4-2).

5.5 Description of the rules for permanent or temporary cessation

5.5.1 An individual's participation in the research

The protocol output will be done

- or by a deliberate decision of the subject (a patient's participation in the research will stop if consent is withdrawn).
- or by necessity, after a decision by the investigator, in the following cases:
    - Non-compliance with the study conditions (poor compliance with any prescribed treatment, intercurrent use of prohibited drugs, etc.)
    - Appearance of an adverse reaction (serious or non-serious)

The trial end form will be completed for any patient who has left the study early.

The usual follow-up will be carried out by the investigator, who will decide on the frequency of follow-up visits.

5.5.2 Some or all of the research

The search may stop temporarily or permanently:

- By decision of the principal investigator
- In the event of knowledge of data compromising the conduct of the study for reasons of patient safety
- In the event of the publication of new scientific data that calls into question research
- In the event of an adverse event considered to be severe and likely to affect the health of patients not clearly established as being unrelated to the research.

5.6 Procedures for monitoring persons

Complications and adverse event monitoring is programmed according to Table 4-2.

Any emergency event will be handled by the investigator.

Any patient, who has experienced an adverse event (or not) that resulted in (or did not result in) his exclusion from the research will be followed by the investigating physician until the event is fully resolved.

6 Treatment used in research subjects

6.1 Description of any medical device or in vitro diagnostic medical device or drug that is used for research purposes including the follow-up period

The realization of the ultrasound, requires the use:

- an ultrasound scanner: the device available at the SDS that will be used in the study is a ***Philips Vivid S6***
- a sterile gel of type K-Y whose composition based on a water-soluble substance does not present any risk in the event of passage into the systemic circulation (gel also used for urinary catheterisation or percutaneous biopsy cytopunctions of the liver or thyroid).
- arterial sampling equipment consisting of a 26 Gauge needle and a 2 cc syringe.

6.2 Drugs and treatments authorised and prohibited under the Protocol, including rescue drugs

There are no drugs prohibited under this protocol.

7 Feasibility

7.1 Patient management

7.1.1 Expected number of people to be included in the research

7.1.1.1 Statistical justification

To our knowledge, data on arterial punctures do not exist in the literature. But the placement of radial arterial catheters can be considered as an example of a comparable procedure. Thus, according to the study by Shiver et al (2006), we can expect a first attempt puncture success rate of 50% in the "no echoguidance" group and a first attempt puncture success rate of 87% in the "with echoguidance" group. »

To highlight a difference in first attempt puncture success rate of 37% between the two groups with a bilateral alpha risk of 5% and a potency of 90%, the total number of subjects to be included is 62 patients. In a conservative spirit, we will increase this figure by 20%, giving us a total of 74 patients or 37 patients per group.

7.1.1.2 Estimation of recruitment capacity

As arterial gas measurement is not rated in the General Emergency Department, we based ourselves on the recruitment potential observed in the first study: in 2 months, 74 patients were recruited (the reasons for the puncture observed are shown in Figure 1 below).

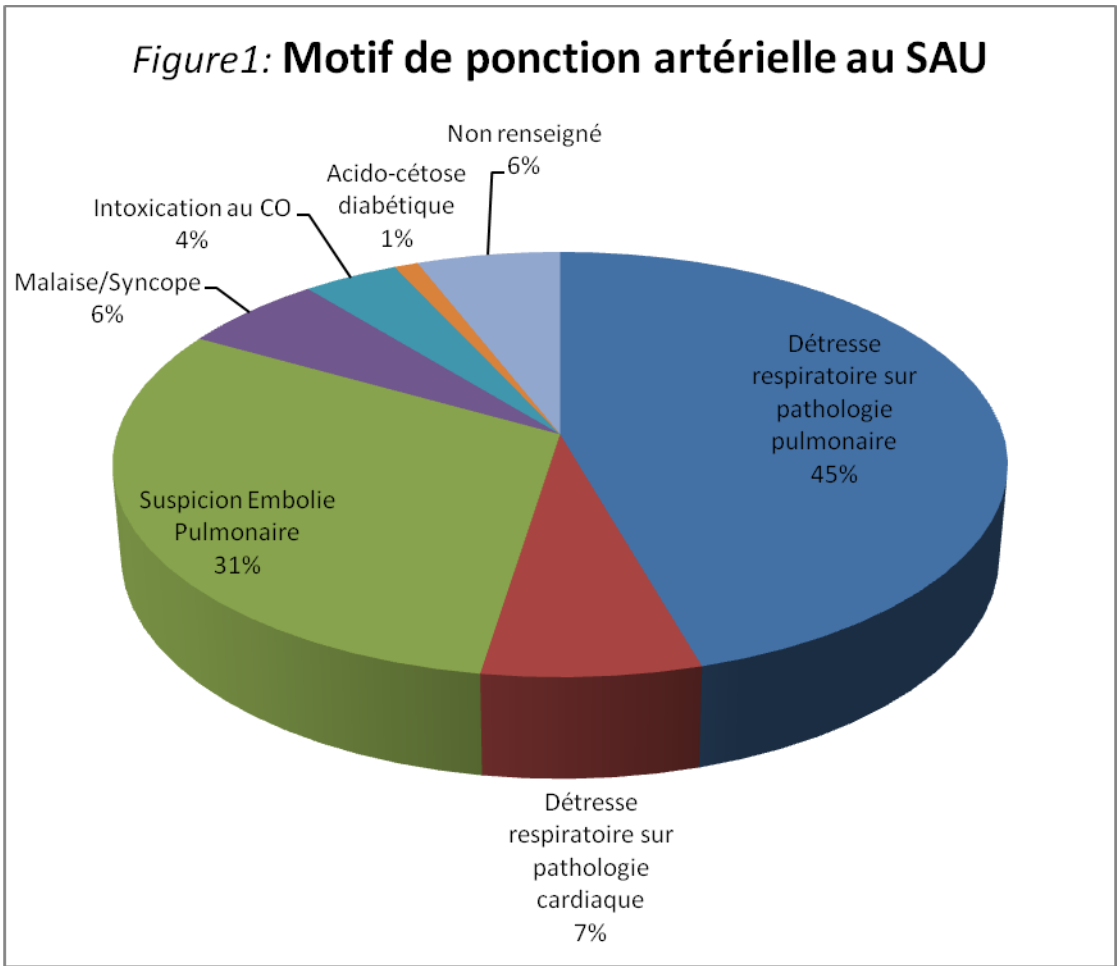

7.1.2 Patient Recruitment

7.1.2.1 Recruitment location

Patients will be recruited upon admission to the Emergency Department.

7.1.2.2 Factors that may affect the quality of representation of the experimental population.

A priori, the recruitment method will provide an experimental population representative of the target population.

However, the reasons for any refusal to participate in the study will be collected. The refusal rate of participation will be compared between patients who had 2 failures and patients with non-palpable arteries.

7.1.3 Patient circuit

The patients' circuit corresponds to that of the usual care. In the "Echoguidage" interventional arm, patients will benefit from an ultrasound puncture aid that will be performed within the UAS.

7.1.4 Patient transport

This research does not require additional travel beyond the usual patient management. It is therefore not necessary to provide specific transport for this study.

7.1.5 Additional information for patients

Not applicable.

7.2 Medical-technical management

The patient circuit will take place entirely within the Emergency Department. No pharmaceutical circuit is necessary for this research; transversal services will not be solicited for the needs of this research.

7.2.1 Pharmaceutical circuit

The authorized treatments correspond to the patient's usual management and are not specific to research. As a result, the supply of the necessary medicines is part of the service's own supply and dispensing circuit.

7.2.2 Biological analyses

Not applicable.

7.2.3 Imaging

In the case of the use of ultrasound, after skin disinfection with hydroalcoholic antiseptic solution, we will apply a type K lubricant gel to the sterile area. This gel is sterile, fat-free, water-soluble and non-irritating. It is suitable for cytoscopic explorations such as liver biopsy punctures or kidney biopsy punctures since it is also used in this context with larger diameter needles without any adverse events being reported. The only contraindications are known allergies to methyl or propylbenzoate or propylene glycol, or chlorinexhidine gluconate. Therefore, the systemic passage of a small amount of gel is not dangerous, except in the case of a known allergy.

7.2.3.1 Identification :

Ultrasound equipment available in the department; use of a 9L linear vascular probe.

All measurements will be made with the same device.

7.2.3.2 Imaging locations

SAU du CHU de Nîmes.

7.3 Randomization and blinding

7.3.1 The drawing of lots

Randomization is performed after checking the inclusion and non-inclusion criteria, and before the first difficult radial arterial puncture. The caregiver who will perform the difficult radial arterial puncture should be designated before randomization (in order to avoid a bias due to the control of the use of ultrasound guidance) which implies that only caregivers able

to perform the puncture with both techniques (palpation and ultrasound guidance) can perform the puncture.

A randomization list will be compiled by the methodologist of the Biostatistics, Clinical Epidemiology, Public Health and Medical Information Department (BESPIM) of the University Hospital of Nîmes. A specially designed SAS program (Cary, NC, USA) will ensure this draw.

In order to ensure that the 2 reasons for inclusion (non-palpable artery or two consecutive failures by the EDI) will be equitably distributed in the 2 groups, randomization will be stratified on this criterion.

A WEB application (Internet®) will be specifically created. After identifying the user, the patient (the first letter of the surname + the first letter of the first name + year of birth) and checking the inclusion and non-inclusion criteria, the type of treatment will be indicated to the user. The use of a WEB application thus allows permanent accessibility and ensures a high degree of security with regard to randomization (impossibility to change the order of randomization, definitive assignment of the patient to a type of treatment and randomization number).

7.3.2 Blinding methods

Not applicable

7.3.3 Measures implemented to maintain blinding and procedures for lifting blinding

Not applicable

7.4 Personnel management

7.4.1 Training to be planned

A presentation of the study is planned for a medical staff in the emergency department.

Only practitioners with a DU or IUD validating for the use of ultrasound, particularly in the vascular area, with theoretical and practical technical training in radial arterial sampling technique, will be eligible to participate in this study.

There is therefore no additional training to be provided except for a demonstration for ghost recall at the presentation meeting.

7.4.2 ARC/TEC time required

A Clinical Study Technician (CST) will assist the investigator in including the patient and collect the available trial data from the patient record. These data will be validated by the principal investigator.

He will keep the investigator folder up to date and will be in charge of archiving the trial.

The estimated time to complete this mission is 0.2 Full Time Equivalent.

An ARC monitoring delegated by the promoter will monitor the study in accordance with the regulations in force.

7.4.3 Meetings to be scheduled

A meeting to set up the study will be organised in the department.

8 Security assessment

8.1 Description of safety assessment parameters

This is the collection of complications and adverse events.

The serial numbers of the ultrasound scanner will be noted in the observation notebooks.

8.2 Planned methods and schedule for measuring, collecting and analyzing security assessment parameters

The collection of data on complications and adverse events is scheduled according to Table 4.1.

8.3 Procedures in place for recording and reporting adverse events

8.3.1 Reporting Responsibilities

According to the Public Health Code, Art L. 1123-10 :

"The adverse events and reactions defined for each type of research shall be reported by the investigator to the sponsor and by the sponsor to the competent authority referred to in Article L. 1123-12* and to the competent persons' protection committee respectively. In this case, the committee shall ensure, if necessary, that the persons participating in the research have been informed of the adverse reactions and that they confirm their consent. »

"Where a new development relevant to the research or product being researched is likely to affect the safety of appropriate persons, the sponsor and the investigator shall take appropriate urgent safety measures. The proponent shall inform the competent authority and the Persons Protection Committee without delay of such developments and, where appropriate, of the measures taken. »

*ANSM : Agence nationale de sécurité du médicament et des produits de santé.

8.3.2 Definitions of the terms

Adverse event: any harmful event occurring in a person who is engaged in biomedical research, whether or not it is related to the research or the product to which the research relates.

Adverse reaction: any adverse event related to the research or product to which this research relates is an adverse reaction.

Serious adverse event / reaction: any event / adverse reaction that

- results in death or endangers the life of the person who is the subject of the search
- requires hospitalization or prolongation of hospitalization

- causes a significant or lasting disability or handicap
- results in a congenital anomaly or malformation
- is medically significant *

*ICH (International Conference of Harmonization) definition applicable in France

Unexpected adverse reaction: Any adverse reaction whose nature, severity or course does not match the information about the products, procedures and methods used in the research.

New fact: Any fact relevant to the research or drug being researched that may affect the safety of suitable persons, leading the sponsor and investigator to take appropriate urgent safety measures.

8.3.3 Description of expected adverse events

In this study, the adverse events described below are considered to be expected:

8.3.3.1 Related to the products / acts practiced / methods used in the research

The risks associated with an arterial puncture with ultrasound guidance are the same as those associated with a puncture without ultrasound guidance:

- Risk of skin break-ins,
- Pain
- Haematomas
- Pseudo-aneurism
- Infection
- Nerve damage
- Vagal reaction

The ultrasound is performed after skin disinfection with hydroalcoholic antiseptic solution and then application of the K-Y gel to the sterile area (data sheet in appendix 16.4): no risk of toxic or infectious contamination

Adverse reactions described in the instructions for use of these products will be considered as expected

8.3.3.2 Linked to the evolution of the disease

Not applicable.

8.3.4 Action to be taken in the event of an adverse event or new fact

All serious and non-serious adverse events, both expected and unexpected, should be recorded in the observation booklet.

All new developments must be recorded in the observation book.

8.3.4.1 Notification of adverse events/reactions to the Sponsor:

The investigator shall inform the DRCI vigilance cell at the University Hospital of Nîmes of any occurrence of an expected or unexpected serious adverse event (SAE), as well as of any new fact likely to affect the safety of persons undergoing research, as soon as he becomes aware of it. Reports of expected and unexpected serious adverse events are made by completing the ISG reporting form and sending it by fax or e-mail to the following address:

Clinical Research and Innovation Department / Vigilance Unit

Nîmes University Hospital - Multi-purpose building
Place du professeur Debré
30029 Nîmes Cedex 9

Tel: 04 66 66 68 67 88 88
Fax: 04 66 68 68 34 33

Mail: vigilec@chu-nimes.fr

The original of this form and the confirmation of shipment are kept in the patient file / observation book.

8.3.4.2 **Reporting serious adverse events to health authorities**:

It is carried out by the vigilance cell of the DRCI of the University Hospital of Nîmes. In accordance with Articles L. 1123-10, R. 1123-42 and R. 1123-48 of the Public Health Code, any suspected unexpected serious adverse reaction and/or new fact is reported by the promoter to the competent authority, the CPP concerned and (where applicable) the other investigators within the legal deadlines.

8.4 Methods and duration of follow-up of individuals following the occurrence of adverse events

Adverse events should be monitored until they are fully resolved.

Additional information relating to the case reported will be sent to the vigilance unit of the DRCI of the University Hospital of Nîmes by fax or e-mail, by completing the declaration follow-up form.

The original of this form and the confirmation of shipment will be kept in the patient file / observation book.

8.5 Oversight Committee

There are no plans to establish an independent oversight committee.

9 Statistics and statistics

9.1 Description of the planned statistical methods, including the timetable for the planned interim analyses

9.1.1 Description of the included population and the main parameters studied

A first analysis of the data will allow the description of the total population and by group. The normality of the distribution of quantitative variables will be explored using the Shapiro-Wilks normality test, as well as the Kurtosis and Skewness coefficients.

The statistical results will be presented as means ± standard deviations for quantitative variables with Gaussian distribution, means and 95% confidence intervals for variables with Gaussian distribution after a transformation, and medians and interquartile intervals for the other variables. For qualitative variables, the numbers and associated percentages will be presented.

9.1.2 Statistical analyses

The statistical analysis will be conducted by the BESPIM of the University Hospital of Nîmes under SAS (SAS Institute, Cary, NC, USA) version 9 or R 2.9.2 (R Development Core Team (2009). R Foundation for Statistical Computing, Vienna, Austria).

9.1.2.1 Verification of the initial comparability of groups

The qualitative variables will be compared by a Chi-2 test. Otherwise, if the conditions for carrying out this test are not met, the exact Fisher test will be used.

The quantitative variables will be compared between the two groups:

- in the case of Gaussian variables, by a student t-test or an analysis of variance
- in case of non-Gaussian variables, by a Mann-Withney- Wilcoxon test

9.1.2.2 Analysis of the main evaluation criterion

The first attempt success rate will be compared between the two groups by a chi2 test or an exact Fisher test. The relative risk of success at the first attempt in the echoguidance group compared to the reference group will be estimated as well as its 95% confidence interval. The number of subjects to be treated with the echoguided technique to achieve success at the first attempt will be estimated as well as its 95% confidence interval.

9.1.2.3 Analysis of secondary judgment criteria

The quantitative secondary judgment criteria (number of skin burns, duration of performance, patient satisfaction VAS, caregiver satisfaction VAS, pain VAS, number of catheters used) will be compared between the 2 groups by a Student test or a Mann Whitney-Wilcoxon test.

The qualitative secondary endpoints (presence of complications, use of additional assistance) will be compared between the 2 groups by a Chi-2 test or an accurate Fisher test.

9.2 Expected level of significance

A difference will be considered statistically significant when the degree of significance of the test is less than or equal to 0.05.

9.3 Statistical criteria for stopping the search

Not applicable.

9.4 Method of taking into account missing, unused or invalid data;

For the main judgment criterion, it is very unlikely that we have missing data.

For the secondary judgment criteria, we do not foresee a method to replace missing data.

9.5 Choice of persons to be included in the analyses

Any patient randomized to the study will also be included in the analyses that will be conducted with the intention of treating. If a patient is included and randomized but no difficult puncture attempt is made (e.g., death before puncture), he or she will be excluded from the analysis.

9.6 Management of changes to the initial statistical plan

The decision to modify the statistical methods provided for in this protocol is taken by the methodologist indicated on page 1.

A change in the statistical method for the analysis of the main criterion must be the subject of an amendment to the protocol.

Any changes in the statistical methods planned for the analysis of the secondary criteria will be reported in the study report.

10 Right of access to source data and documents

10.1 Right of access of the promoter and the competent authority to source data and documents

Investigators agree to comply with the requirements of the sponsor and the Competent Authority for an audit or inspection of the study.

The audit may be applied at all stages of the study, from the development of the protocol to the publication of results and the classification of the data used or produced as part of the study.

10.2 Mention of the submission to the CNIL

This study is part of the "Reference Methodology" MR-001 in application of the provisions of article 54 paragraph 5 of the amended law of 6 January 1978 on data processing, files and freedoms.

The Nîmes University Hospital, promoter of the study, has undertaken to respect this reference methodology and received, on May 9, 2006, the MR 001 declaration of conformity receipt.

11 Quality control and assurance

11.1 Monitoring & quality control

A clinical research assistant, delegated by the sponsor (CRA sponsor), will visit each study centre on a regular basis when the trial is set up, once or several times during the study depending on the rate of inclusions and at the end of the study.

The purpose of these visits is:

- to check compliance with the protocol,
- to verify informed consents,
- to verify the notification of Serious Adverse Events,
- to monitor the traceability of the study's medical devices (visit to the pharmacy, storage and accounting of medicines),
- to ensure quality control: compare the data in the observation book with the centre's source documents.

Persons responsible for quality control of biomedical research and duly mandated for this purpose by the sponsor shall have access, subject to the agreement of the persons concerned, to the individual data strictly necessary for such control; they shall be subject to professional secrecy.

All visits will be subject to a monitoring report by written report (traceability of visits).

11.2 Audit and inspection

Investigators agree to comply with the requirements of the sponsor and the Competent Authority for an audit or inspection of the study.

The audit may be applied at all stages of the study, from the development of the protocol to the publication of results and the classification of the data used or produced as part of the study.

12 Ethical considerations

The research shall be conducted in accordance with this Protocol. Except in emergency situations requiring the implementation of specific therapeutic procedures, the investigator(s) undertake to comply with the protocol in all respects, in particular with regard to the collection of consent and the reporting and monitoring of serious adverse events.

The protocol will be submitted to the Comité Protection des Personnes Sud Méditerranée III of Nîmes in accordance with the regulations in force.

An information letter will be presented to the patient, specifying: the purpose, objectives and conduct of the study in accordance with the regulations, as well as their rights to refuse to participate in the study or to leave it at any time.

The patient's consent will be sought and obtained before the patient enters the study. A copy of the signed consent will be provided, one copy will be retained by the investigator, the last copy will be retained by the sponsor.

Minors, or adults subject to a legal protection measure, cannot be included.

There will be no emergency situation as long as it is a scheduled response.

12.1 Duty of the investigator

The investigator undertakes that this study will be carried out in accordance with Law No. 2004-806 of 9 August 2004 on public health policy and its implementing decrees, the Helsinki Declaration and Good Clinical Practices.

All data, documents and reports may be subject to regulatory audits and inspections without the possibility of medical confidentiality.

All information collected is confidential and may not be disclosed. The investigator will ensure that the anonymity of each patient participating in the study is guaranteed. No information allowing the identification of persons will be communicated to third parties other than those, representing the promoter and the Ministry of Health, who are authorised by law to hold this information (and who are bound by professional secrecy).

12.1.1 The possibility for the investigator to book certain information

There is the possibility for the investigator, exceptionally, in accordance with his or her confidence, to reserve certain information related to the diagnosis (when in the interest of a sick person the diagnosis of his or her illness could not be revealed to him or her).

12.2 Proponent's obligation

In accordance with Act No. 2004-806 of 9 August 2004 on public health policy and its implementing decrees, the promoter undertakes to carry out all the operations for which it is responsible:

Insurance: The University Hospital of Nîmes, promoter of the study, subscribes for the entire duration of the study an insurance guaranteeing its own civil liability as well as that of any intervener involved in the realization of the trial, independently of the nature of the existing links between the interveners and the promoter.

Competent Authority : The proponent will seek authorization from ANSM. The research will only be carried out after the PPC has given a positive opinion and the competent authority has given its authorisation.

Commission Nationale Informatique et Liberté: The information collected during this study may be processed electronically. The file will be produced in accordance with the CNIL (Commission Nationale de l'Informatique et des Libertés).

12.2.1 Amendment to the Protocol

Any substantial modification, i.e. any modification likely to have a significant impact on the protection of persons, on the conditions of validity and results of research, on the quality and safety of tested products, on the interpretation of scientific documents supporting the conduct of the research or on the methods of conducting it, shall be the subject of a written amendment submitted to the sponsor; the latter shall obtain, before its implementation, a favourable opinion from the CPP and an authorisation from the competent authority.

Non-substantial changes, i.e. those that do not have a significant impact on any aspect of the research, are communicated to the PPC for information purposes.

All amendments to the protocol must be made known to all investigators involved in the research. Investigators undertake to respect its content.

Any amendment that modifies patient management or the benefits, risks and constraints of the research is the subject of a new information note and a new consent form, the collection of which follows the same procedure as that described above.

12.3 Justification for the collection of ethnic or racial information, if applicable.

Not applicable.

13 Data processing and storage of documents and research data

13.1 Data collection and management

The patient will only be identified by a unique 2-character identification number (P☐☐), and the first letter of the surname, the first letter of the first name, and the year of birth.

A subject identification list will be kept in the investigator's file.

The investigator will ensure that the anonymity of each person participating in the study is guaranteed. No information allowing the identification of persons will be communicated to third parties other than those authorised by law to hold this information (and who are bound by professional secrecy).

Information will be collected for each patient on a standardized observation logbook completed by the investigator or co-investigator. This workbook will include subject identification, characteristics of the initial pathology and follow-up data.

An observation booklet will be kept for each subject participating in the study; the observation and medical follow-up regarding the study should be recorded in the source document.

All study data will be transcribed into the observation booklet and/or stored on a computer by the investigating physicians or their delegate(s). The observation notebooks shall be filled in legibly and indelibly with a blue or black ballpoint pen. In the event of an error, the incorrect information will be crossed out with a single line, the initial data will remain visible, and the correct information will be written next to it. Each correction will be justified and authenticated (dated and signed or initialled by the investigator). The principal investigator will sign each observation booklet to attest to his agreement with the data contained therein.

The data will be analysed by the Methodology and Clinical Research Unit of the BESPIM of the University Hospital of Nîmes.

13.2 Data Entry and Data Management Procedures

Data entry:

The seizure will be carried out by a designated person.

This entry will be made on an e-CRF directly or from the paper version of the observation book.

Only persons participating in the research project and identified will have access to the software allowing this entry: OpenClinica.

OpenClinica is a software that generates electronic CRFs (e-CRFs), electronically captures, manages and restores data. It also makes it possible to monitor the progress of studies in terms of data entry and to manage them. It is an open source software that is scalable, modular and based on international standards.

OpenClinica allows:

- Data entry, validation and annotation by clinicians and researchers
- Data extraction, filtering and analysis by investigators
- Study management by study coordinators
- Monitoring, auditing, configuration and reporting by administrators

The entry in the e-CRF is controlled and formatted to prevent the entry of off-target or outliers data. In the event of a modification of the data entry, traceability and monitoring of activities is ensured. An electronic signature engaging the responsibility of the investigator of each centre will allow the validation of the visit and the e-CRF.

This software is hosted on a website within the University Hospital of Nîmes. Access to this application is secure and is via https://oc.bespim.fr with a username and password. The data collected through this software is backed up daily on a secure network. The network is connected to the Internet, access is protected by a firewall.

The clinical data of the study will be stored on a specific directory of the server. Only network administrators and authorized persons from the clinical research unit of the medical information department can access this directory.

The data extraction for analysis will be carried out either by a person in charge of the BESPIM department at the Nîmes University Hospital or by the "data manager" who will have the necessary rights in the application.

13.3 Conservation and Archiving

The closure of the trial, including the closure of the centres, will be carried out in accordance with Good Clinical Practices and ICH. Medical, administrative and observation records will be kept for the duration of the study in the department and archived for a minimum period of 30 years.

Computer measures taken to implement the confidentiality commitment

- The technical resources are located in the BESPIM department, access to which is controlled and secure.
- The servers on which the data is stored are backed up daily.

Backups are made on magnetic media such as tapes. A seven-day and a one-month band rotation is performed.

The hard disks of the servers are mounted "in raid" which guarantees a high level of security. No data storage tapes are stored in the machine room. The weekly storage tapes are stored in a locked cabinet in the absence of the user(s) authorized by the study sponsor.

Each month, a monthly backup tape is stored in a geographically different location with controlled and locked access.

14 Financing and insurance

14.1 Duration of the insurance coverage and specific terms and conditions for covering risks beyond

The duration of the insurance coverage taken out by the promoter is 1 year

14.2 Acts and processing specific to research

In the interventional arm, an ultrasound will be performed within the UAS.

14.3 Total amount needed for research

The total amount of expenses allocated to this study amounts to 8930.00€.

14.4 Sources of funding

All the expenses of this study will be covered by the University Hospital of Nîmes.

15 Rules on publication

15.1 Study Report :

The data analysis will be carried out by BESPIM, Nîmes University Hospital.

An official report dated and signed by the investigator will be sent to the sponsor, who will forward it to the Persons Protection Committee and the competent authority within 12 months of the end of the study.

The investigator acknowledges that the results of the study are the property of the sponsor.

15.2 Publication Rules :

Any written or oral communication of the results of the research must receive the prior consent of the coordinating investigator and, where applicable, of any research committee.

15.2.1 Mention of the University Hospital of Nîmes, Nîmes, France

For publications in French-speaking journals, the affiliation for all authors of the article will be written in French, with the acronym CHU noted without space and without points.

Example: service of..., University Hospital of Nîmes, Nîmes, France

When authors publish in English-language journals, the affiliation for all authors of the article will be written in English, the CHU will be cited as follows: Department of..., Nîmes University Hospital, Nîmes, France

The contact details of the corresponding author will be written in French to ensure the delivery of any correspondence.

When the Nîmes University Hospital is the promoter, the affiliation of the authors to the Nîmes University Hospital is mandatory and must be at the top of the list.

15.2.2 Articles and Conferences

Any written or oral communication of the results of the research must have the name of at least one permanent holder of the University Hospital of Nîmes among the authors.

Account will be taken of the international rules for writing and publishing defined by the International Committee of Medical Journal Editors (ICMJE).

15.2.3 Press Releases and Reactive Nîmes

The publication of an article in a newspaper of rank A is reported to the Communication Department of the CHU Nîmes.

15.3 Communication of results to patients

In accordance with law n°2002-303 of 4 March 2002, patients are informed, at their request, of the overall results of the research.

15.4 Data transfer

Data management is provided by the BESPIM (Biostatistics, Clinical Epidemiology, Public Health, & Medical Information Department (BESPIM), Nîmes University Hospital, Place du Professeur Debré, 30029 Nîmes Cedex 09). The conditions for the transfer of all or part of the research database are decided by the research sponsor and are the subject of a written contract.

16 Appendices

16.1 Patient information letter

Patient information letter

Interest of ultrasound guidance in "difficult" radial arterial blood puncture.

Version n°2 of March 5th, 2013

RCB NUMBER: **2012-A01525-38**

Promoter of the study: **Centre Hospitalier Universitaire de Nîmes**
Place du Professeur Debré
30029 Nîmes Cedex 09

Presentation of the study

Dear Sir or Madam,

As part of your care at the Emergency Department (EDS), blood must be collected by radial artery (wrist artery) for diagnostic purposes. The sampling consists of taking a blood sample from the radial artery. This may require several attempts, especially if your arteries are not easily identifiable by palpation. We believe that an ultrasound guidance (guidance through an ultrasound examination) of this procedure could allow a better success rate from the first attempt and consequently a reduction in the time required to perform it and increased comfort for you.

This is what we propose to evaluate in a clinical study entitled "Interest of ultrasound guidance in difficult radial arterial puncture" in which Dr. GENRE-GRANDPIERRE proposes to participate. This study will determine whether the use of ultrasound equipment can result in a gain for patients in terms of the duration of the procedure and the pain experienced.

This note is intended to provide you with information about this study. If you do not understand certain words or elements of this note well, do not hesitate to ask the doctor for explanations.

Signing the consent form will attest to your final agreement to participate in this study.

The subjects included in the study must be affiliated or beneficiaries of a social security scheme.

Objectives of the study

The main objective of this study is to determine whether the use of an ultrasound scanner in the emergency department to perform a "difficult" radial arterial puncture (due to an unpalpable artery or after two consecutive failures) improves the success rate at the first attempt.

Failure is defined as an insufficient amount of blood collected, no blood flow or accidental collection from the vein instead of the artery.

The secondary objectives of this study will be to determine whether the use of an ultrasound scanner reduces the number of needle sticks required for successful blood collection, the duration of the procedure, immediate complications (hematoma, malaise, etc.), and an improvement in the pain experienced, as well as patient and caregiver satisfaction.

Study methodology

Following the inclusion of a patient in the study, a randomization draw allows the patient to be assigned either to the reference management group ("palpation") or to the interventional management group ("ultrasound guidance"). The comparison of these two groups provides the methodological framework for this research.
The chances of falling into either group are 50/50.

Number of patients

74 patients admitted to the emergency department of the University Hospital of Nîmes will be included in the study.

Conduct of the study and number of visits

The study consists of a single visit that corresponds to the performance of a radial arterial puncture; during this procedure, no modification of the care usually performed will be performed other than an ultrasound examination in the group with interventional management ("ultrasound guidance"). During this visit you will be asked to estimate the pain felt and your satisfaction after the procedure has been performed.

Constraints and Obligations

The volume of blood collected will be 2 ml as for any sample for the determination of blood gases.

The only additional constraint will be to assess your pain and satisfaction using a visual analogue scale (a kind of scale graduated from 0 to 10).

Potential benefit expected for the participant and compensation

The expected benefit in the interventional arm (puncture with ultrasound guidance) would be an increase in the success rate at the first attempt and a decrease in the duration of the act. However, this hypothesis must be confirmed, which is why we are carrying out this study.

All processing and investigations will be fully covered within the framework of the research and, if necessary, you will be entitled to reimbursement of the expenses incurred.

This study does not give rise to any compensation.

Risks arising from research

The risks associated with an arterial puncture with ultrasound guidance are the same as those associated with a puncture without ultrasound guidance: pain, haematomas, pseudo-aneurysm (partial damage to the artery), infection, nerve damage, vagal reaction.

Rights and guarantees of individuals

As part of this research, your personal data will be processed in order to allow the results to be analysed in the light of the objective of this research. To this end, the medical data concerning you, identified by a code number, will be transmitted to the sponsor. They may also, under conditions ensuring their confidentiality, be transmitted to the French health authorities.

In accordance with the provisions of the "Informatique et Libertés" law (law n° 2004-801 of 6 August 2004), you may have a right of access and rectification. You also have the right to object to the transmission of data covered by professional secrecy that may be used in the context of the research. You can access all your medical data directly or through a doctor of your choice in accordance with the provisions of Article L 1111-7 of the Public Health Code.

These rights are exercised with the doctor in charge of the study.

If you wish, the overall results of this work will be communicated to you at its conclusion.

You will be able to benefit from additional information about the research at any time and any new knowledge that may call your participation into question will be communicated to you.

In accordance with the law of 4 March 2002 on the rights of patients, you have the right to be accompanied by a trusted person for any medical act involving your consent.

Your attending physician may be informed of your participation in this project, unless you object.

Regulatory Data

In accordance with the laws and regulations, the University Hospital of Nîmes, under the number 131562, has subscribed an insurance policy covering the Civil Liability of the Promoter of Biomedical Research with the ,18 rue Edouard Rochet - 69372 LYON Cedex 08).

The terms of this protocol have been submitted for examination to the Comité de Protection des Personnes (CPP) Sud Méditerranée III, whose mission is to verify whether the conditions required for your protection and the respect of your rights are respected.

This committee issued a favourable opinion on :……………………

This protocol was approved by the competent authority (ANSM) on……………………..

You are completely free to accept or refuse to participate in this study. Your doctor will give you the time you need to make your decision.

If you have any questions, do not hesitate to ask the doctor.

Name and contact details of the principal investigator:
**Dr Romain GENRE-GRANDPIERRE**<br>**Emergency Service**<br>**Nîmes University Hospital**

Please be assured that your participation is extremely valuable to us. We thank you in advance, Madam, Sir, for the help you are giving to medical research.

Investigator's name and signature :………………………

16.2 Free and Informed Consent Form for the patient participating in biomedical research

Free and Informed Consent Form for the patient participating in biomedical research

Read carefully the different parts of this document. Sign this document only after reading and completing all parts (3 copies, I for the sponsor, I for the local investigator and I for the patient).

Interest of ultrasound guidance in "difficult" radial arterial blood puncture.

Interest of ultrasound guidance in "difficult" radial arterial blood puncture.

Version n°1 of July 26th, 2012

Version n°1 of July 26th, 2012

*RCB NUMBER: **2012-A01525-38***

*RCB NUMBER: **2012-A01525-38***

I, the undersigned……………………………………………………………………………, born (e), ……………………………………agree to participate, under the conditions defined in the newsletter, in the research designated above conducted by Dr Romain GENRE-GRANDPIERRE.

- I certify that I am a member or beneficiary of a health insurance plan.

Faculty of opposition and withdrawal of consent:

- I understand that my participation in this study is voluntary and that I may refuse it. If I wish, I will be **FREE AT ANY TIME DURING THE STUDY TO STOP MY PARTICIPATION** without having to justify myself. I will inform the principal investigator of the study, Dr. GENRE-GRANDPIERRE, in writing at the following address: Emergency Department, Nîmes University Hospital, Place du Professeur Debré, 30029 Nîmes Cedex 09.

- I understand that my decision to participate in this study does not release either the researchers or the host institution from their professional and legal obligations to me.
- I have read my right to access and rectify personal information concerning me that is processed automatically. The right of access and rectification provided for by the "Informatique et Liberté" law is exercised at any time with the persons in charge of the study.

Recognition of prior information:

- The doctor informed me of the nature and aims of this research project, as well as how it was conducted and the possible benefits and risks of the study.
- In this respect, I have in my possession an information note (Version N°1 of 26 July 2012) that I have read and understood.
- I was able to ask all the questions I wanted about this project and I got satisfactory answers. I acknowledge that I have had sufficient time to reflect between this information and this consent and that I have had the opportunity, if I so wished, to discuss it with my doctor or relatives.
- In particular, I acknowledge that I have been informed of the right to be assisted by a trusted person of my choice.
- I acknowledge that I have been informed that the study may be interrupted at any time by decision of the sponsor or the authorities, that all measures will be taken in this case to ensure my safety and, if necessary, the continuation of my treatment, and that my personal participation may be suspended if I do not comply with the protocol.
- I am aware that any new facts that may affect my consent to my participation in the study will be communicated to me.

Acceptance of constraints:

- I undertake to observe the constraints explained and specified in the information document, both to minimize risks and for the successful completion of the protocol.
- I formally certify that I have not participated in any biomedical research since 3 months and that I have not participated in any ongoing studies.
- I accept the prohibition I have been told not to participate in any other research at the same time.
- I have understood that hiding the truth can have detrimental consequences for my health. I therefore certify that I have answered all the questions asked of me in a sincere manner, in particular those relating to my state of health and lifestyle.
- I undertake to respect the confidentiality rules applicable to the study, as explained to me beforehand.

Other authorizations :

- As part of this study, all DATA AND INFORMATION concerning me will remain strictly CONFIDENTIAL. I only allow their consultation by persons designated by the investigators and possibly by a representative of the health authorities. I am informed

that this clinical data collected will be subject to computerized processing authorized by the National Commission for Information Technology and Freedom.

- In accordance with the law of 4 March 2002, relating to patients' rights, I know that I have the possibility of being accompanied by a trusted person for any medical act involving my consent.

Date and signature of the patient:	Date and signature of the investigator:
______________________	______________________
______________________	______________________

Proponent of the study:

University Hospital Centre of Nîmes

Place du Pr Debré

30029 Nîmes Cedex 09

16.3 Visual Analogue Scale (VAS)

<u>Instructions for the patient</u>:

Draw a vertical line **(|)** through the point on the scale below that describes the level of pain (satisfaction) you feel, considering "0" as the absence of any pain (satisfaction), and "10" as intolerable pain (total satisfaction).

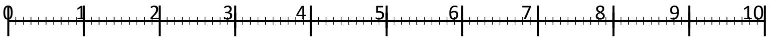

No pain	Unbearable pain
Totally dissatisfied	Total satisfaction

Enter your initials, date and time of measurement below:

Subject's initials: ___________

Date: : _____ /_____ / /_____

16.4 Références

Andersen, A., K. Milojevic, A. Nédélec-Jaffuel, S. Silva, A. Beltramini, R. Ruiz Almenar, C. Barbier, et J. L. Ricôme. 2009. Intérêt des gaz du sang en préhospitalier pour les patients ventilés: étude prospective. *Journal Européen des Urgences* 22: A119.

Bauman, M., D. Braude, et C. Crandall. 2009. Ultrasound-guidance vs. standard technique in difficult vascular access patients by ED technicians. *American Journal of Emergency Medicine* 27, n°. 2: 135-140.

Chabot, F., E. Gomez, A. Guillaumot, A. Kheir, et A. Chaouat. 2009. Exacerbations de bronchopneumopathie chronique obstructive= Acute exacerbations of chronic obstructive pulmonary disease. *La Presse médicale* 38, n°. 3: 485-495.

Costantino, T. G., A. K. Parikh, W. A. Satz, et J. P. Fojtik. 2005. Ultrasonography-guided peripheral intravenous access versus traditional approaches in patients with difficult intravenous access. *Annals of emergency medicine* 46, n°. 5: 456-461.

Donati, S. Y., M. Gainnier, et O. Chibane-Donati. 2005. Intoxication au Monoxyde de carbone. *EMC-Anesthésie-Réanimation* 2, n°. 1: 46-67.

Dudeck, O., Teichgraeber, U., Podrabsky, P., Lopez Haenninen, E., Soerensen, R., Ricke, J. 2004. A randomized trial assessing the value of ultrasouns-guided puncture of the femoral artery for interventional investigations. Int J Cardiovascular Imaging 20:363-368

Franklin, C. 1995. The technique of radial artery cannulation. Tips for maximizing results while minimizing the risk of complications. *The Journal of Critical Illness* 10, n°. 6 (Juin): 424-432.

Ganesh, A., R. Kaye, A. M. Cahill, W. Stern, R. Pachikara, P. R. Gallagher, et M. Watcha. 2009. Evaluation of ultrasound-guided radial artery cannulation in children. *Pediatric Critical Care Medicine* 10, n°. 1: 45.

Giner, J., P. Casan, J. Belda, H. Litvan, et J. Sanchis. 2000. Use of the anesthetic cream EMLA in arterial punction. *Revista española de anestesiologia y reanimacion* 47, n°. 2: 63.

Gratrix, A. P., J. D. Atkinson, et A. R. Bodenham. 2009. Cannulation of the impalpable section of radial artery: preliminary clinical and ultrasound observations. *European journal of anaesthesiology* 26, n°. 10: 887.

Kannan, S. 2005. Another use for ultrasound in the ICU. Anaesthesia 60: 944.

Levin, PD., Sheinin, O., Gozal, Y. 2003. Use of ultrasound guidance in the insertion of radial artery catheters. Critical Care Medicine 31, n° 2: 481-484.

Lorut, C. 2005. Embolies pulmonaires. *EMC-Cardiologie-Angéiologie* 2, n°. 4: 531-541.

Maggiore, S. M., et P. Jolliet. 2005. Quels outils et quels paramètres ventilatoires peuvent permettre d'adapter au mieux les réglages du ventilateur et quelle est leur place dans la surveillance ventilatoire au cours du Syndrome de Détresse Respiratoire Aiguë?=

Which tools and parameters can be useful for ventilatory settings and monitoring in Acute Respiratory Distress Syndrome? *Réanimation* 14, n°. 5: 359-366.

Marshall, B. E. 1964. METHOD OF ARTERIAL SAMPLING. *The Lancet* 284, n°. 7349: 40.

Martin, C. 1995. Cathétérisme artériel et mesure invasive de la pression artérielle en anesthésie-réanimation chez l'adulte= Arterial catheterisation and invasive measurement of arterial blood pressure in anaesthesia and intensive care in adults. Dans *Annales françaises d'anesthésie et de réanimation*, 14:444-453. Elsevier.

Micu, E., C. Guillot, M. Badier, S. Delpierre, J. M. Régis, et P. Roussel. 2006. Pain induced by radial artery puncture is not reduced by lidocaine-prilocaine patch. *Respiratory Medicine Extra* 2, n°. 1: 52-53.

Mizukoshi, K., Shibasaki, M., Amaya, F., Hirayama, T., Shimizu, F., Hosokawa, K., Hashimoto, S., Tanaka, Y. 2009. Ultrasound evidence of the optimal wrist position for radial artery cannulation. Can J Anesth 56: 427-431.

Nagabhushan, S., Colella, JJ., Wagner, R. 1976. Use of Doppler ultrasound in performing percutaneous cannulation of the radial artery. Crit Care Med 4:327.

Sandhu, NS., Patel, B. 2005. Use of ultrasonography as a rescue technique for failed radial artery cannulation. J Clinical Anesthesia 18: 138-141.

Sarrot-Reynauld, F., et B. Paramelle. 2003. La ponction artérielle radiale pour analyse des gaz du sang. Etude du vécu des patients. *Revue de Medecine Interne* 24, n°. 4: 484.

Scheer, Bernd, Azriel Perel, et Ulrich J Pfeiffer. 2002. Clinical review: complications and risk factors of peripheral arterial catheters used for haemodynamic monitoring in anaesthesia and intensive care medicine. *Critical Care (London, England)* 6, n°. 3 (Juin): 199-204.

Schwemmer, U., Arzet, HA., Traunter, H., Rauch, S., Roewer, N., Greim, CA. 2006. Ultrasound-guided arterial cannulation in infants improves success rate. Eur. J. Anaesthesiol. 23, 476-480.

Shiloh, AL, Eisen, LA. 2010. Ultrasound-guided arterial catheterization: a narrative review. Intensive Care Med 36: 214-221.

Shiver, S., M. Blaivas, et M. Lyon. 2006. A prospective comparison of ultrasound-guided and blindly placed radial arterial catheters. *Academic Emergency Medicine* 13, n°. 12: 1275-1279.

Spunda, R., T. Urban, J. Tosovský, J. Táborský, et M. Semrád. 2005. Postcatheterization pseudoaneurysm of the radial artery. *Rozhledy v chirurgii: m sí ník eskoslovenské chirurgické spole nosti* 84, n°. 5: 244.

Taytard, A., J. P. Daures, P. Arsac, J. L. Chirumberro, J. P. Grignet, M. Micoud, R. Poirier, P. Romand, J. M. Tartavel, et D. Touron. 2001. Prise en charge des infections respiratoires basses en médecine générale en France. *Rev Mal Respir* 18: 163-170.

Wilson, SR., Grunstein, I., Hirvela, ER., Price, DD. 2010. Ultrasound-guided radial artery catheterization and the modified Allen's test. J of Emerg Med 38, n°3, 354-358.
