# Supplementary material for: Ultrasound guidance in difficult radial artery puncture for blood gas analysis: A prospective, randomized controlled trial
Source: PLoS One. 2019 Mar 20;14(3):e0213683. doi: 10.1371/journal.pone.0213683 (PMC6426205; doi:10.1371/journal.pone.0213683)
Supplement: S2 File — Original protocol in French. (PDF) [file pone.0213683.s002.pdf]

Intérêt de l'échoguidage dans la ponction artérielle sanguine radiale « difficile ».

Dr. Romain GENRE GRANDPIERRE

Protocole de recherche clinique

1 Informations générales

1.1 Identification

Intérêt de l'ECHOguidage dans la Ponction Artérielle sanguine Radiale « DIFficile ».

Etude ECHO-PARDIF

N° RCB: 2012-A01525-38
N° de code du protocole : Local/2012/RGG-01
N° de version et date du protocole : Version N°11 du 7 mars 2016
Type d'appel d'offre : Local 2012
Type d'étude : essai monocentrique, pilote, comparatif, contrôlé à 2 bras parallèles, randomisé, réalisé en ouvert
Source de financement : CHU de Nîmes
Durée du projet (année) : 3 ans
Nombre total de sujets à inclure : 74

1.2 Principaux correspondants

Promoteur :
CHU de Nîmes, Direction de la Recherche et de l'Innovation, Place du Professeur Debré, 30029 Nîmes Cedex 09 ; Tél : 04.66.68.42.36 ; Fax : 04.66.68.34.00 ; email : drc@chu-nimes.fr

Nom et qualité de l'investigateur principal :
Dr Romain GENRE-GRANDPIERRE, Service des Urgences, DARDU, CHU de Nîmes, Place du Professeur Debré, 30029 Nîmes Cedex 09 ; Tél : 04.66.68.30.50 ; Fax : 04.66.6838.51 ; email : romainsbs@hotmail.com

Nom et qualité de la ou des personnes autorisées à signer le protocole et ses modifications éventuelles au nom du promoteur :
Anissa Megzari, Directrice de la Recherche, des Partenariats et des Projet Médicaux.
CHU de Nîmes, Direction, Place du Professeur Debré, 30029 Nîmes Cedex 09 ; Tél : 04.66.68.42.36 ; Fax : 04.66.68.34.00 ; email : drc@chu-nimes.fr

Méthodologiste :
Dr. Christophe Demattei (PhD), Service de Service de Biostatistique, Epidémiologie Clinique, Santé Publique et Information Médicale (BESPIM), CHU de Nîmes, Place du Professeur Debré, 30029 Nîmes Cedex 09 ; Tél : 04.66.68.35.53 ; Fax : 04.66.68.34.33 ; email : christophe.demattei@chu-nimes.fr

1.3 Approbation du protocole

Nom Date et Signature du Représentant légal du Promoteur :	Nom Date et Signature de l'Investigateur Coordonnateur ou Investigateur Principal :
Le 10 mars 2016<br>La Directrice de la Recherche Clinique, des Partenariats, et des Projets Médicaux<br>Anissa MEGZARI	Le 10 mars 2016<br>Dr Romain GENRE GRANDPIERRE

1.4 Historique des mises à jour du protocole

Version	Date	Raison de la mise à jour
1	26 Juillet 2012	Création
2	30 Août 2012	Relecture et modifications CD
3	31 Août 2012	Corrections PR
4	7 Septembre 2012	Corrections RGG
5	7 Septembre 2012	Version avant CS
6	24 Septembre 2012	Version finale
7	21 Novembre 2012	CPP
8	12 décembre 2012	Corrections CD
9	5 mars 2012	Réponse avis différé CPP
10	22 octobre 2014	MS 1
11	7 mars 2016	MS 2

1.5 Liste des investigateurs

Titre. Prénom NOM	*Adresse du centre*	*Téléphone - Fax - email*
**Dr. Romain GENRE-GRANDPIERRE**<br>***Investigateur principal***<br>*Qualification : PH*<br>*Spécialité : Médecine d'Urgence*	CHU de Nîmes<br>Service des Urgences<br>Place du Professeur Debré<br>30029 Nîmes Cedex 09	Tél : 04 66 68 30 50<br>Fax : 04 66 68 38 51<br>romainsbs@hotmail.com
**Dr. Patrick Richard**<br>***Investigateur***<br>*Qualification : PH*<br>*Spécialité : Médecine d'Urgence*	CHU de Nîmes<br>Service Urgences-Réanimation<br>Place du Professeur Debré<br>30029 Nîmes Cedex 09	Tél : 04.66.68.36.28<br>Fax : 04.66.68.38.51<br>patrick.richard@chu-nimes.fr<br>N° RPPS : 10003196747
**Dr. Xavier BOBBIA**<br>***Investigateur***<br>*Qualification : PH*<br>*Spécialité : Médecine d'Urgence*	CHU de Nîmes<br>Service des Urgences<br>Pôle A.R.D.U.<br>Place du Professeur Debré<br>30029 Nîmes Cedex 09	Tél : 04 66 68 42 24<br>Fax : 04 66 68 38 51<br>xavier.bobbia@chu-nimes.fr
**Dr. Rémi PERRIN-BAYARD**<br>***Investigateur***<br>*Qualification : PH*<br>*Spécialité : Médecine d'Urgence*	CHU de Nîmes<br>Service des Urgences<br>Pôle A.R.D.U.<br>Place du Professeur Debré<br>30029 Nîmes Cedex 09	Tél : 06 08 32 68 70<br>Fax : 04 66 68 38 51<br>remspb@yahoo.fr
**Dr. Stéphane POMMET**<br>***Investigateur***<br>*Qualification : PH*<br>*Spécialité : Médecine d'Urgence*	CHRU de Nîmes<br>Services des Urgences-SAMU 30<br>Place du Professeur Debré<br>30029 Nîmes Cedex 09	Tél : 04.66.68.30.50<br>Fax : 04.66.68.38.51<br>stephane.pommet@free.fr<br>N° RPPS : 10100029379
**Dr. Alexandre MOREAU**<br>***Investigateur***<br>*Qualification : PH*	CHRU de Nîmes<br>Services des Urgences-SAMU 30<br>Place du Professeur	Tél : 04.66.68.30.50<br>Fax : 04.66.68.38.51<br>Duphalac69@yahoo.fr

Titre. Prénom NOM	*Adresse du centre*	*Téléphone - Fax - email*
Spécialité : Médecine d'Urgence	Debré<br>30029 Nîmes Cedex 09	N° RPPS : 1010097871
**Dr. Pierre-Géraud CLARET**<br>***Investigateur***<br>*Qualification : PH*<br>*Spécialité : Médecine d'Urgence*	CHU de Nîmes<br>Service d'Accueil des Urgences<br>Pôle ARDU<br>Place du Professeur Debré<br>30029 Nîmes Cedex 09	Tél : 04 66 68 80 83<br>Fax : 04 66 68 38 51<br>pierre.geraud.claret@gmail.com<br>N° RPPS : 10003255907
**Dr. Yan BODIN**<br>***Investigateur***<br>*Qualification : PH*<br>*Spécialité : Médecine d'Urgence*	CHU de Nîmes<br>Service Urgences-Réanimation<br>Place du Professeur Debré<br>30029 Nîmes Cedex 09	Tél : 04.66.68.36.28<br>Fax : 04.66.68.38.51<br>yan.bodin@chu-nimes.fr<br>N° RPPS : 10100187318
**Dr. Emmanuelle GERVAIS**<br>***Investigateur***<br>*Qualification : PH*<br>*Spécialité : Médecine d'Urgence*	CHU de Nîmes<br>Service des Urgences<br>Pôle A.R.D.U.<br>Place du Professeur Debré<br>30029 Nîmes Cedex 09	Tél : 04 66 68 42 24<br>Fax : 04 66 68 38 51<br>emmanuelle.gervais@chu-nimes.fr<br>N° RPPS : 10100215424
**Dr. Aurélie COTTEL**<br>*Collaborateur*<br>*Qualification : PH*<br>*Spécialité : Médecine d'Urgence*	CHU de Nîmes<br>Service des Urgences<br>Pôle A.R.D.U.<br>Place du Professeur Debré<br>30029 Nîmes Cedex 09	Tél : 06 08 32 68 70<br>Fax : 04 66 68 38 51<br>aurelie.cottel@chu-nimes.fr<br>N° RPPS : 10100215390
**Dr. Nadine HANSEL**<br>*Collaborateur*<br>*Qualification : PH*<br>*Spécialité : Médecine d'Urgence*	CHRU de Nîmes<br>Services des Urgences-SAMU 30<br>Place du Professeur Debré<br>30029 Nîmes Cedex 09	Tél : 04.66.68.30.50<br>Fax : 04.66.68.38.51<br>nadine.hansel@chu-nimes.fr<br>N° RPPS : 10100029379
**Dr. Charlotte BOYARD**<br>*Collaborateur*<br>*Qualification : PH*	CHRU de Nîmes<br>Services des Urgences-SAMU 30<br>Place du Professeur	Tél : 04.66.68.30.50<br>Fax : 04.66.68.38.51<br>Charlotte.boyard@chu-nimes.fr

Titre. Prénom NOM	*Adresse du centre*	*Téléphone - Fax - email*
Spécialité : Médecine d'Urgence	Debré<br>30029 Nîmes Cedex 09	N° RPPS : 10005181051
**Dr. Patricia WAGNER**<br>*Collaborateur*<br>*Qualification : PH*<br>*Spécialité : Médecine d'Urgence*	CHU de Nîmes<br>Service d'Accueil des Urgences<br>Pôle ARDU<br>Place du Professeur Debré<br>30029 Nîmes Cedex 09	Tél : 04 66 68 80 83<br>Fax : 04 66 68 38 51<br>patricia.wagner@ chu-nimes.fr<br>N° RPPS : 10003201083
**Dr. Aurélie DARDALHON**<br>*Collaborateur*<br>*Qualification : PH*<br>*Spécialité : Médecine d'Urgence*	CHU de Nîmes<br>Service des Urgences<br>Pôle A.R.D.U.<br>Place du Professeur Debré<br>30029 Nîmes Cedex 09	Tél : 04 66 68 42 24<br>Fax : 04 66 68 38 51<br>N° RPPS : 10003248951
**Dr. François HERNANDEZ**<br>*Collaborateur*<br>*Qualification : PH*<br>*Spécialité : Médecine d'Urgence*	CHU de Nîmes<br>Service des Urgences<br>Pôle A.R.D.U.<br>Place du Professeur Debré<br>30029 Nîmes Cedex 09	Tél : 06 08 32 68 70<br>Fax : 04 66 68 38 51<br>aurelie.cottel@chu-nimes.fr<br>N° RPPS : 10100215390
**Dr. Nadine HANSEL**<br>*Collaborateur*<br>*Qualification : PH*<br>*Spécialité : Médecine d'Urgence*	CHRU de Nîmes<br>Services des Urgences-SAMU 30<br>Place du Professeur Debré<br>30029 Nîmes Cedex 09	Tél : 04.66.68.30.50<br>Fax : 04.66.68.38.51<br>Francois.hernandez@chu-nimes.fr<br>N° RPPS : 10003199873
**Dr. Michael ROBERT**<br>*Collaborateur*<br>*Qualification : PH*<br>*Spécialité : Médecine d'Urgence*	CHRU de Nîmes<br>Services des Urgences-SAMU 30<br>Place du Professeur Debré<br>30029 Nîmes Cedex 09	Tél : 04.66.68.30.50<br>Fax : 04.66.68.38.51<br>michael.robert@chu-nimes.fr<br>N° RPPS : 10100165884
**Dr. Hélène ROBERT**<br>*Collaborateur*	CHU de Nîmes<br>Service d'Accueil des Urgences	Tél : 04 66 68 80 83<br>Fax : 04 66 68 38 51

Titre. Prénom NOM	*Adresse du centre*	*Téléphone - Fax - email*
Qualification : PH<br>*Spécialité : Médecine d'Urgence*	Pôle ARDU<br>Place du Professeur Debré<br>30029 Nîmes Cedex 09	Helene.robert@ chu-nimes.fr<br>N° RPPS : 10003256285
**Dr. Andrew STOWELL**<br>*Collaborateur*<br>*Qualification : PH*<br>*Spécialité : Médecine d'Urgence*	CHRU de Nîmes<br>Services des Urgences-SAMU 30<br>Place du Professeur Debré<br>30029 Nîmes Cedex 09	Tél : 04.66.68.30.50<br>Fax : 04.66.68.38.51<br>andrew.stowell@chu-nimes.fr<br>N° RPPS : 10100158053
**Dr. Jacques CRAMPAGNE**<br>*Collaborateur*<br>*Qualification : PH*<br>*Spécialité : Médecine d'Urgence*	CHRU de Nîmes<br>Services des Urgences-SAMU 30<br>Place du Professeur Debré<br>30029 Nîmes Cedex 09	Tél : 04.66.68.30.50<br>Fax : 04.66.68.38.51<br>jacques.crampagne@chu-nimes.fr<br>N° RPPS : 10003201117
**Dr. Jean FLECHET**<br>*Collaborateur*<br>*Qualification : PH*<br>*Spécialité : Médecine d'Urgence*	CHU de Nîmes<br>Service d'Accueil des Urgences<br>Pôle ARDU<br>Place du Professeur Debré<br>30029 Nîmes Cedex 09	Tél : 04 66 68 80 83<br>Fax : 04 66 68 38 51<br>Jean.flechet@ chu-nimes.fr<br>N° RPPS : 10003245510

1.6 Liste des abréviations et définitions

ARC : Attaché de Recherche Clinique

BESPIM : service de Biostatistique, Epidémiologie clinique, Santé Publique, & Information Médicale

EVA : Echelle Visuelle Analogique

IMC : Indice de Masse Corporelle

PAC : Pontage Aortocoronarien

SAU : Service d'Accueil des Urgences

TEC : Technicien d'Etudes Cliniques

1.7 Document additionnel

1. Numéro RCB

2012-A01525-38

2. Titre complet de la recherche

Intérêt de l'échoguidage dans la ponction artérielle sanguine radiale « difficile ».

3. Justification et analyse critique de la pertinence de la recherche

La ponction de gaz de sang artériel est un geste très fréquent dans les services de médecine, de réanimation ou d'accueil des urgences (8 à 12% des patients). La technique de prélèvement a été décrite dès le dix-neuvième siècle puis réactualisée. Elle consiste après désinfection par une solution antiseptique, en un repérage de l'artère par palpation suivi de l'introduction d'une aiguille avec un afflux spontané de sang artériel dans la seringue. La voie privilégiée reste la voie radiale plutôt que les voies fémorales ou axillaires car l'artère y est superficielle, facilement accessible, relativement éloignée des veines adjacentes.

Ce geste très pratiqué n'est cependant pas totalement sans risques. Même si les complications restent rares (moins de 1% pour les cathéters), la ponction artérielle peut être source de complications vasculaires (pseudo-anévrisme, hématome), d'infection ou de lésion d'un nerf), mais aussi de mauvais vécu de la part du patient suite à des douleurs, à un nombre élevé de tentatives ou d'échecs.

Diverses solutions ont été étudié afin de tenter de diminuer le mauvais ressenti lié au geste comme en particulier l'utilisation de pommade anesthésiante de type EMLA, mais sans résultat significatif. L'utilisation de la technique d'imagerie ultrasonographique sur les ponctions veineuses périphériques difficiles a montré des résultats encourageants en termes de nombre de tentatives, de durée de réalisation et de nombres de complications. Cette expérience acquise pourrait être appliquée à la ponction artérielle et des cas ont été décrits dès le milieu des années 1970.

Il n'existe cependant que très peu d'études randomisées évaluant l'intérêt de l'échographie dans la mise en place de cathéters artériels; Levin et al. (2003) dans une étude portant sur 69 adultes devant subir une chirurgie, a démontré un taux de succès de 60% à la première tentative grâce à l'utilisation de l'échographie comparé au taux de 34% dans le groupe palpation traditionnelle. Sur 60 patients nécessitant une cathétérisation randomisés en salle d'urgence, Shiver et al (2006) démontrent un taux de réussite au premier essai de 87% dans le groupe échographie versus 50% dans le groupe palpation. Dans une étude portant sur la cathétérisation de 30 enfants avant neurochirurgie, Schwemmer et al (2006) décrivent un taux de succès de 100% dans le groupe avec échographie (avec un taux de réussite de 67% à la première tentative) alors qu'il n'est que de 80% dans le groupe palpation.

Cependant d'autres études randomisées décrivent des résultats plus équivoques. Ainsi sur 152 patients pédiatriques devant subir une chirurgie, Ganesh et al. (2009) ont trouvé que l'échographie ne facilitait pas une cathétérisation artérielle radiale plus rapide de la part d'anesthésistes inexpérimentés tant dans les techniques de palpation que d'échographie ; de plus il n'y avait pas de différence entre les deux groupes en termes de taux de succès, de nombre de tentatives ou de nombre de cathéters utilisés. Tada et al. (2003) n'ont pas

trouvé de bénéfice à utiliser un Doppler bidirectionnel dans la cathétérisation artérielle de routine chez 166 patients devant subir une anesthésie générale.

Nous avons réalisé en 2010, une étude préliminaire qui a permis de valider l'échographie comme technique de prélèvement de sang artériel radial puisque nous avons pu montrer qu'elle ne majore pas l'estimation de la douleur ressentie au cours de la ponction et qu'elle donne satisfaction aux patients. Cependant, elle majore le temps de réalisation du geste ce qui n'est pas négligeable aux urgences en termes de gestion du temps médical et de la qualité de la prise en charge du patient. Or nous avions retenu pour cette première étude tous les patients se présentant au SAU du CHU de Nîmes et dont l'état de santé nécessitait à des fins diagnostique, pronostique ou thérapeutique une ponction artérielle sanguine.

Dans leur étude, Ganesh et al. (2009) ont constaté qu'un opérateur expérimenté en échographie réussissait à de nombreuses occasions à réaliser la ponction sous échographe alors que l'opérateur initial avait échoué par la technique de la palpation.

Ces constatations nous conduisent à formuler l'hypothèse que l'utilisation de l'échoguidage pourrait être validée comme alternative à la technique classique dans le cas de ponctions jugées difficiles. C'est ce que nous chercherons à évaluer dans cette étude où ne seront inclus que les patients dont les artères radiales ne sont pas palpables ou ayant subi deux échecs de ponction par l'IDE.

4. Hypothèse principale de la recherche et objectifs

Nous formulons l'hypothèse que le recours au guidage par échographie lors de ponctions artérielles radiales difficiles permettra une amélioration du taux de réussite à la première tentative, une réduction du nombre d'effractions cutanées et de complications immédiates.

L'objectif principal de cette étude est de déterminer si la réalisation, sous échographie, d'une ponction artérielle radiale « difficile » (artère non palpable ou deux échecs consécutifs par l'IDE) au SAU permet une amélioration du taux de réussite à la première tentative. L'échec est défini par une quantité de sang prélevé insuffisante, l'absence de reflux sanguin ou une ponction veineuse accidentelle.

Les objectifs secondaires de cette étude sont de déterminer si l'utilisation d'un échographe pour la réalisation d'une ponction artérielle radiale « difficile » au SAU permet :

A. Une diminution du nombre d'effractions cutanées
B. Une diminution de la durée de réalisation (min)
C. Une diminution des complications immédiates (hématome, lésion d'un nerf, réaction vagale, pseudo-anévrisme)
D. Une amélioration du ressenti patient vis-à-vis de l'acte (EVA)
E. Une amélioration du ressenti du personnel soignant (EVA)
F. Une amélioration de la douleur ressentie par le patient (EVA)
G. Une diminution du nombre de cathéters utilisés
H. Une diminution du recours à une assistance additionnelle

5. Evaluation des bénéfices et des risques que présente la recherche, notamment les bénéfices escomptés pour les personnes qui se prêtent à la recherche et les risques prévisibles liés au

traitement et aux procédures d'investigation de la recherche (incluant notamment la douleur, l'inconfort, l'atteinte à l'intégrité physique des personnes se prêtant à la recherche, les mesures visant à éviter et/ou prendre en charge les événements inattendus)

L'échoguidage pourrait permettre une augmentation du nombre de réussites à la première tentative comme déjà observé dans le cas de ponctions veineuses périphériques difficiles, donc une diminution du nombre d'effractions cutanées.

Les risques associés avec une ponction artérielle avec échoguidage sont les mêmes que pour une ponction sans échoguidage : risques d'effractions cutanées, douleur, hématomes, pseudo-anévrisme, infection, lésion d'un nerf, réaction vagale.

6. Justification de l'inclusion des personnes visées aux articles L.1121-5 à L.1121-8 et L.1122-1-2 du code de la santé publique (ex. : mineurs, majeurs protégés, etc.) et procédure mise en œuvre afin d'informer et de recueillir le consentement de ces personnes ou de leurs représentants légaux

1. Justifications de l'inclusion de personnes visées aux articles L. 1121-5 à L. 1121-8 et L. 1122-1-2 du code de la santé publique:

Les patients mineurs, les femmes enceintes, les parturientes, les mères qui allaitent, les personnes en situation d'urgence ainsi que celles qui sont sous sauvegarde de justice ne sont pas inclus dans l'étude.

2. Procédures mises en œuvre pour informer :

Les patient(e)s seront recruté(e)s dans le service des Urgences. Lorsqu'un(e) patient(e) répond aux critères de sélection, il(elle) sera informé(e) par l'investigateur de l'existence de l'étude. Au cours de cet entretien, les objectifs ainsi que les contraintes liées à la réalisation de l'étude et ses droits seront explicitées au patient qui pourra poser toutes les questions qu'il souhaite.

Une fois que cette information aura été donnée et que l'investigateur sera convaincu que le(la) patient(e) comprend les implications de sa participation à l'étude, le (la) patient(e) sera libre d'accepter ou de refuser de participer à l'étude d'où il (elle) pourra se retirer à tout moment et ce quelle qu'en soit la raison, et sans encourir aucune responsabilité. Quel qu'en soit le motif, le refus de participer n'aura aucune conséquence sur la qualité des soins que continuera de donner l'investigateur ni sur la qualité des relations médecin-malade.

Conformément à l'article R.5120 du code de la santé publique, les investigateurs ainsi que toutes les personnes appelées à collaborer à l'étude sont tenus au secret professionnel en ce qui concerne notamment la nature de l'étude, les personnes qui s'y prêtent et les résultats obtenus. L'investigateur s'assurera que l'anonymat des patients sera respecté. Sur l'ensemble des documents, les patients seront identifiés par un code numérique.

La signature du consentement libre et éclairé du sujet sera recueillie pendant la visite d'inclusion. Il sera laissé au patient un délai de réflexion de quelques minutes entre la présentation de la note d'information et la signature du consentement.

7. Description des modalités de recrutement des personnes

Il s'agit d'un essai monocentrique, pilote, comparatif, contrôlé à 2 bras parallèles, randomisé, réalisé en ouvert.

Les patients seront recrutés lors de leur admission au Service d'Accueil des Urgences.

Critères d'inclusion des patients : le patient doit avoir donné son consentement libre et éclairé et signé le consentement, doit être affilié ou bénéficiaire d'un régime d'assurance maladie, être âgé d'au moins 18 ans. L'état de santé du patient doit nécessiter la réalisation d'une ponction artérielle sanguine radiale et les artères radiales du patient sont non palpables ou il y a eu deux échecs préalables de ponction par l'IDE.

Critères de non inclusion des patients : le sujet participe à une autre étude, est en période d'exclusion déterminée par une étude précédente, est sous sauvegarde de justice, sous tutelle ou sous curatelle. Le sujet refuse de signer le consentement, il s'avère impossible de donner au sujet des informations éclairées. La patiente est enceinte, parturiente, ou elle allaite. La ponction doit être réalisée sur l'artère fémorale, humérale ou axillaire. Le patient est en arrêt cardio-respiratoire, présente des troubles circulatoires connus, une ischémie, une infection locale. Le patient présente des troubles de la coagulation ou de l'hémostase sévère (hémophilie, hypoprothrombinémie, plaquettes < 50000). Le patient est amputé d'un membre supérieur ou a son bras plâtré. Le patient présente des allergies connues au méthyl ou propylbenzoate, au propylène glycol ou au gluconate de chlorexhidine. Le patient présente une fistule artérioveineuse ou une malformation artério-veineuse.

8. Procédures d'investigation menées et différences par rapport à la prise en charge habituelle

Une seule visite d'inclusion/fin de recherche est nécessaire pour la réalisation de cette étude.

Suite à l'inclusion d'un patient, une randomisation est faite pour l'assigner soit au groupe prise en charge de référence (« palpation »), soit au groupe prise en charge interventionnelle (« échoguidage »). La comparaison de ces deux groupes constitue le cadre méthodologique de cette recherche.

La douleur ressentie, la satisfaction du patient et du soignant seront évaluées à l'aide d'une EVA.

En dehors de la réalisation d'une échographie dans le bras interventionnel, la prise en charge du patient est inchangée par rapport à la pratique médicale habituelle. Aucun examen biologique spécifique n'est associé à cette étude.

La durée d'inclusion sera de 30 mois. La durée totale de l'essai sera de 3 ans.

9. Justification de l'existence ou non : (1) d'une interdiction de participer simultanément à une autre recherche ; (2) d'une période d'exclusion pendant laquelle la participation à une autre recherche est interdite.

Les patients ayant participé à une étude dans les 3 mois précédant leur inclusion dans ce protocole sont exclus dans le but d'éliminer toute interférence (connue ou non) qui pourrait conduire à sur ou sous estimer les potentielles modifications dans l'appréciation de critères d'évaluation au cours de l'étude et donc d'en fausser les conclusions. Les patients inclus ont interdiction de participer simultanément à une autre recherche interventionnelle pour les mêmes raisons.
Il n'y aura pas de période d'exclusion à la fin de l'essai.

10. Modalités et montant de l'indemnisation des personnes se prêtant à la recherche

Il n'est pas prévu d'indemniser les personnes se prêtant à la recherche.

11. Motifs de constitution ou non d'un comité de surveillance indépendant

Il n'est pas prévu de constituer un comité de surveillance indépendant.

12. Nombre prévu de personnes à inclure dans la recherche

74 soit 37 par bras

1.8 Sommaire

1 Informations générales _____ 2
1.1 Identification _____ 2
1.2 Principaux correspondants _____ 2
1.3 Approbation du protocole _____ 2
1.4 Historique des mises à jour du protocole _____ 3
1.5 Liste des investigateurs _____ 4
1.6 Liste des abréviations et définitions _____ 8
1.7 Document additionnel _____ 9
1.8 Sommaire _____ 13
2 Justification scientifique et description générale de la recherche _____ 17
2.1 Etat actuel des connaissances _____ 17
2.1.1 La ponction artérielle radiale _____ 17
2.1.2 Hypothèses de recherche _____ 19
2.1.3 Originalité et retombées attendues de l'étude _____ 19
2.2 Résumé des bénéfices et des risques prévisibles et connus pour les personnes se prêtant à la recherche _____ 19
2.2.1 Bénéfices _____ 19
2.2.2 Risques _____ 19
2.3 Déclaration indiquant que la recherche sera conduite conformément au protocole, aux bonnes pratiques et aux dispositions législatives et réglementaires en vigueur _____ 20
2.4 Références _____ 20
3 Objectifs de la recherche _____ 20
3.1 Objectif principal _____ 20
3.2 Objectifs secondaires _____ 20
4 Conception de la recherche _____ 21
4.1 Méthodologie _____ 21
4.1.1 Type d'essai et caractéristiques _____ 21
4.1.2 Critères d'évaluation _____ 23
4.1.3 Cofacteurs _____ 23
4.2 Les visites et examens prévus _____ 23
4.2.1 Visite de pré-inclusion _____ 24

4.3 Description des paramètres d'évaluation et des méthodes pour mesurer, recueillir et analyser ces paramètres ____ 26
4.3.1 Les observations cliniques ____ 26
4.3.2 Les échelles visuelles analogiques ____ 26
4.3.3 Les ponctions artérielles ____ 26
4.3.4 Les critères extraits des la base de données ____ 27
4.4 Identification de toutes les données à recueillir directement dans les cahiers d'observation ____ 27
5 Sélection et exclusion des personnes de la recherche ____ 27
5.1 Description de la population à étudier ____ 27
5.2 Critères d'inclusion des personnes qui se prêtent à la recherche ____ 27
5.3 Critères de non-inclusion des personnes qui se prêtent à la recherche ____ 28
5.4 Durée prévue de participation des personnes, et description de la chronologie et de la durée de toutes les périodes de la recherche, y compris le suivi ____ 28
5.4.1 Durée prévue de participation d'un sujet ____ 28
5.4.2 Durée prévue de la recherche ____ 29
5.4.3 Fin de la recherche ____ 29
5.5 Description des règles d'arrêt définitif ou temporaire ____ 30
5.5.1 De la participation d'une personne à la recherche ____ 30
5.5.2 D'une partie ou de la totalité de la recherche ____ 30
5.6 Modalités de surveillance des personnes ____ 30
6 Traitement utilisé chez les personnes qui se prêtent à la recherche ____ 31
6.1 Description de tout dispositif médical ou dispositif médical de diagnostic in vitro ou médicament qui est utilisé pour les besoins de la recherche y compris pour la période de suivi 31
6.2 Médicaments et traitements autorisés et interdits dans le cadre du protocole, y compris les médicaments de secours ____ 31
7 Faisabilité ____ 31
7.1 Gestion des patients ____ 31
7.1.1 Nombre prévu de personnes à inclure dans la recherche ____ 31
7.1.2 Recrutement des patients ____ 32
7.1.3 Circuit des patients ____ 33
7.1.4 Transports des patients ____ 33
7.1.5 Informations supplémentaires pour les patients ____ 33
7.2 Gestion médico-technique ____ 33
7.2.1 Circuit pharmaceutique ____ 33
7.2.2 Analyses biologiques ____ 33
7.2.3 Imagerie ____ 33

7.3 Randomisation et mise à l'insu 34
7.3.1 Le tirage au sort 34
7.3.2 Les méthodes de mise en insu 34
7.3.3 Dispositions mises en œuvre en vue du maintien de l'insu et procédures de levée de l'insu 34
7.4 Gestion du personnel 34
7.4.1 Formations à prévoir 34
7.4.2 Temps d'ARC/TEC nécessaire 35
7.4.3 Réunions à prévoir 35
8 Evaluation de la sécurité 35
8.1 Description des paramètres d'évaluation de la sécurité 35
8.2 Méthodes et calendrier prévus pour mesurer, recueillir et analyser les paramètres d'évaluation de sécurité 35
8.3 Procédures mises en place en vue de l'enregistrement et de la notification des événements indésirables 35
8.3.1 Responsabilités en matière de déclarations 35
8.3.2 Définitions 36
8.3.3 Description des événements indésirables attendus 36
8.3.4 Conduite à tenir en cas d'évènement indésirable ou de fait nouveau 37
8.4 Modalités et durée du suivi des personnes suite à la survenue d'événements indésirables 38
8.5 Comité de surveillance 38
9 Statistiques 38
9.1 Description des méthodes statistiques prévues, y compris du calendrier des analyses intermédiaires prévues 38
9.1.1 Description de la population incluse et des principaux paramètres étudiés 38
9.1.2 Analyses statistiques 38
9.2 Degré de signification prévu 39
9.3 Critères statistiques d'arrêt de la recherche 39
9.4 Méthode de prise en compte des données manquantes, inutilisées ou non valides ; 39
9.5 Choix des personnes à inclure dans les analyses 39
9.6 Gestion des modifications apportées au plan statistique initial 39
10 Droit d'accès aux données et documents source 40
10.1 Droit d'accès du promoteur et de l'autorité compétente aux données et documents source 40
10.2 Mention de la soumission à la CNIL 40
11 Contrôle et assurance de la qualité 40
11.1 Monitorage & contrôle de qualité 40

11.2 Audit et inspection ____ 41
12 Considérations éthiques ____ 41
12.1 Obligation de l'investigateur ____ 41
12.1.1 La possibilité pour l'investigateur de réserver certaines informations ____ 42
12.2 Obligation du promoteur ____ 42
12.2.1 Amendement au protocole ____ 42
12.3 Justification du recueil d'informations à caractère ethnique ou racial, le cas échéant. 42
13 Traitement des données et conservation des documents et des données relatives à la recherche ____ 43
13.1 Recueil et gestion des données ____ 43
13.2 Saisie des Données et Procédures de Data management ____ 43
13.3 Conservation et Archivage ____ 44
14 Financement et assurance ____ 45
14.1 Durée de la garantie d'assurance et modalités particulières de couverture des risques au-delà 45
14.2 Actes et traitements propres à la recherche ____ 45
14.3 Montant total nécessaire pour la recherche ____ 45
14.4 Sources de financement ____ 45
15 Règles relatives à la publication ____ 45
15.1 Rapport d'Etude : ____ 45
15.2 Règles de Publication : ____ 45
15.2.1 Mention du CHU de Nîmes, Nîmes, France ____ 45
15.2.2 Articles et Conférences ____ 46
15.2.3 Les Communiqués de Presse et Reactif Nîmes ____ 46
15.3 Communication des résultats aux patients ____ 46
15.4 Cession des données ____ 46
16 Annexes ____ 47
16.1 Lettre d'information destinée au patient ____ 47
16.2 Formulaire de Consentement Libre et Eclairé pour le(la) patient(e) participant à une recherche biomédicale ____ 52
16.3 Echelle Visuelle Analogique (EVA) ____ 55
16.4 Références ____ 56

2 Justification scientifique et description générale de la recherche

2.1 Etat actuel des connaissances

2.1.1 La ponction artérielle radiale

La ponction de gaz de sang artériel est un geste très fréquent dans les services de médecine (Sarrot-Reynauld et Paramelle 2003), de réanimation ou d'accueil des urgences (8 à 12% des patients).

L'étude des gaz du sang est une aide établie au diagnostic et au suivi de nombreuses pathologies telles que les maladies trombo-emboliques (Lorut 2005), les pathologies respiratoires en décompensation (Chabot et al. 2009; Taytard et al. 2001) ou non (Taytard et al. 2001), les patients ventilés en pré-hospitalier (Andersen et al. 2009) et en hospitalier (Maggiore et Jolliet 2005), les perturbations de l'équilibre acido-basique ou encore les intoxications au monoxyde de carbone (Donati, Gainnier, et Chibane-Donati 2005).

La technique de prélèvement a été décrite dès le dix-neuvième siècle puis réactualisée (Marshall 1964). Elle consiste après désinfection par une solution antiseptique, en un repérage de l'artère par palpation suivi de l'introduction d'une aiguille avec un afflux spontané de sang artériel dans la seringue (Franklin 1995). La voie privilégiée reste la voie radiale plutôt que les voies fémorales ou axillaires (Martin 1995) car l'artère y est superficielle, facilement accessible, relativement éloignée des veines adjacentes.

Ce geste très pratiqué n'est cependant pas totalement sans risques. Même si les complications restent rares (moins de 1% pour les cathéters (Scheer, Perel, et Pfeiffer 2002)), la ponction artérielle peut être source de complications vasculaires (pseudo-anévrisme, hématome), d'infection ou de lésion d'un nerf (Sarrot-Reynauld et Paramelle 2003; Spunda et al. 2005)), mais aussi de mauvais vécu de la part du patient suite à des douleurs, à un nombre élevé de tentatives ou d'échecs (Sarrot-Reynauld et Paramelle 2003).

Diverses solutions ont été étudiées afin de tenter de diminuer le mauvais ressenti lié au geste comme en particulier l'utilisation de pommade anesthésiante de type EMLA (Giner et al. 2000; Micu et al. 2006), mais sans résultat significatif.

L'utilisation de la technique d'imagerie ultrasonographique sur les ponctions veineuses périphériques difficiles (Costantino et al. 2005; Bauman, Braude, et Crandall 2009) a montré des résultats encourageants en termes de nombre de tentatives, de durée de réalisation et de nombres de complications. Cette expérience acquise pourrait être appliquée à la ponction artérielle et des cas ont été décrits dès le milieu des années 1970 (Nagabhushan et al. 1976). Les études montrent qu'en plus de la cathétérisation artérielle standard à des fins de monitorage, les ultrasons ont aussi été utilisés pour guider des procédures interventionnelles (Shiloh et Eisen 2010). Quelques rapports de cas ont identifié de nouvelles utilisations pour le guidage échographique en cathéthérisation artérielle. Kannan (2005) décrit un cas de cathéthérisation de l'artère brachiale par assistance échographique en service de soins intensifs, chez un patient présentant un œdème important chez lequel les essais par la technique classique restaient infructueux. Sandhu et Patel (2005) a reporté quelques cas où l'utilisation des ultrasons pouvait être une alternative de secours dans le cas

d'échec de canulations d'artére radiale car elle permet de franchir les obstacles que peuvent constituer les œdèmes, vasospasme ou hématome. Wilson et al. (2010) rapportent un cas où l'échographie est validée aux urgences comme un outil de guidage de la cathéthérisation de l'artère radiale chez un patient dans l'incapacité de coopérer pour le traditionnel test de Allen.

Il n'existe cependant que très peu d'études randomisées évaluant l'intérêt de l'échographie dans la mise en place de cathéters artériels (Shiloh et Eisen 2010) ; Levin et al. (2003) dans une étude portant sur 69 adultes devant subir une chirurgie, a démontré un taux de succès de 60% à la première tentative grâce à l'utilisation de l'échographie comparé au taux de 34% dans le groupe palpation traditionnelle. Sur 60 patients nécessitant une cathétérisation randomisés en salle d'urgence, Shiver et al (2006) démontrent un taux de réussite au premier essai de 87% dans le groupe échographie versus 50% dans le groupe palpation.

Dans une étude portant sur la cathétérisation de 30 enfants avant neurochirurgie, Schwemmer et al (2006) décrivent un taux de succès de 100% dans le groupe avec échographie (avec un taux de réussite de 67% à la première tentative) alors qu'il n'est que de 80% dans le groupe palpation. Une des principales raisons de la difficulté de cathétérisation chez l'enfant est bien sûr la faible section de l'artère radiale rendant la technique de palpation difficile. Schwemmer et al. (2006) décrivent en effet une section de l'artère radiale comprise entre 1 et 4,2 mm2, cette surface étant réduite de 19% par une dorsiflexion du poignet. Mizukoshi et al. (2009) ont d'ailleurs évalué le positionnement optimal du poignet par échoguidage lors de la canulation de l'artère radiale chez 17 patients sains et chez 17 patients devant subir des pontages aortocoronariens (PAC) ; les dimensions de l'artère radiale ont été mesurées à des angles du poignet de 0°, 15°, 30°, 45°, 60° et 75°. Chez les patients sains, les dimensions de l'artère radiale ne changent pas lorsque l'articulation du poignet est augmentée jusqu'à un angle de 45°. Une extension à 60° chez les patients sains et à 75° chez les patients subissant un PAC a toutefois pour résultat une réduction de la hauteur de l'artère radiale, ce qui pourrait rendre la cathétérisation artérielle plus difficile.

Dans ces trois premières études, un nombre de tentatives ainsi que de cathéters significativement plus bas a été nécessaire pour le positionnement de cathéters dans l'artère radiale sous échographie. Egalement, la ponction artérielle radiale sous échographie a nécessité significativement moins de temps que les techniques plus traditionnelles. Cependant d'autres études randomisées décrivent des résultats plus équivoques. Ainsi sur 152 patients pédiatriques devant subir une chirurgie, Ganesh et al. (2009) ont trouvé que l'échographie ne facilitait pas une cathétérisation artérielle radiale plus rapide de la part d'anesthésistes inexpérimentés tant dans les techniques de palpation que d'échographie ; de plus il n'y avait pas de différence entre les deux groupes en termes de taux de succès, de nombre de tentatives ou de nombre de cathéters utilisés. Tada et al. (2003) n'ont pas trouvé de bénéfice à utiliser un Doppler bidirectionnel dans la cathétérisation artérielle de routine chez 166 patients devant subir une anesthésie générale. Enfin en 2004, Dudeck et al. dans le cas de ponction de l'artère fémorale lors d'investigations interventionnelles chez 112 patients ont conclu que l'échographie devrait être utilisé dans le cas de patients ayant un pouls artériel faible ou une circonférence de jambe supérieure ou égale à 60 cm ; chez ces

sujets, on observait une diminution du nombre de tentatives et une diminution du temps nécessaire à la ponction par comparaison avec la technique de palpation traditionnelle.

Nous avons réalisé en 2010, une étude préliminaire qui a permis de valider l'échographie comme technique de prélèvement de sang artériel radial puisque nous avons pu montrer qu'elle ne majore pas l'estimation de la douleur ressentie au cours de la ponction et qu'elle donne satisfaction aux patients. Cependant, elle majore le temps de réalisation du geste ce qui n'est pas négligeable aux urgences en termes de gestion du temps médical et de la qualité de la prise en charge du patient. Or nous avions retenu pour cette première étude tous les patients se présentant au SAU du CHU de Nîmes et dont l'état de santé nécessitait à des fins diagnostique, pronostique ou thérapeutique une ponction artérielle sanguine.

Dans leur étude, Ganesh et al. (2009) ont constaté qu'un opérateur expérimenté en échographie réussissait à de nombreuses occasions à réaliser la ponction sous échographe alors que l'opérateur initial avait échoué par la technique de la palpation.

Ces constatations nous conduisent à formuler l'hypothèse que l'utilisation de l'échoguidage pourrait être validée comme alternative à la technique classique dans le cas de ponctions jugées difficiles. C'est ce que nous chercherons à évaluer dans cette étude où ne seront inclus que les patients dont les artères radiales ne sont pas palpables ou ayant subi deux échecs de ponction par l'IDE.

2.1.2 Hypothèses de recherche

Nous formulons l'hypothèse que le recours au guidage par échographie lors de ponctions artérielles radiales difficiles permettra une amélioration du taux de réussite à la première tentative, une réduction du nombre d'effractions cutanées et de complications immédiates.

2.1.3 Originalité et retombées attendues de l'étude

Les retombées attendues de cette étude sont un changement des pratiques professionnelles lors des ponctions de gaz de sang artériel.

2.2 Résumé des bénéfices et des risques prévisibles et connus pour les personnes se prêtant à la recherche

2.2.1 Bénéfices

L'échoguidage pourrait permettre une augmentation du nombre de réussites à la première tentative comme déjà observé dans le cas de ponctions veineuses périphériques difficiles, donc une diminution du nombre d'effractions cutanées.

2.2.2 Risques

Les risques associés avec une ponction artérielle avec échoguidage sont les mêmes que pour une ponction sans échoguidage : risques d'effractions cutanées, douleur, hématomes, pseudo-anévrisme, infection, lésion d'un nerf, réaction vagale.

2.3 Déclaration indiquant que la recherche sera conduite conformément au protocole, aux bonnes pratiques et aux dispositions législatives et réglementaires en vigueur

L'investigateur s'engage à travailler selon les Bonnes Pratiques Cliniques définies par la décision du 24 Novembre 2006 par le Ministère de la santé et des Solidarités, et selon la réglementation en vigueur (Code de santé Publique).

L'investigateur s'engage, pour lui-même et pour toutes les personnes amenées à suivre le déroulement de l'étude, à garantir la confidentialité de toutes les informations portant sur le projet jusqu'à la publication des résultats de l'essai. Cette obligation de confidentialité ne s'appliquera pas aux renseignements que l'investigateur sera amené à communiquer aux patients dans le cadre de leur participation à l'essai ni aux informations déjà publiées.

2.4 Références

Voir l'annexe 16.4 en fin de protocole.

3 Objectifs de la recherche

3.1 Objectif principal

L'objectif principal de cette étude est de déterminer si la réalisation, sous échographie, d'une ponction artérielle radiale « difficile » (artère non palpable ou deux échecs consécutifs par l'IDE) au SAU permet une amélioration du taux de réussite à la première tentative. L'échec est défini par une quantité de sang prélevé insuffisante, l'absence de reflux sanguin ou une ponction veineuse accidentelle.

3.2 Objectifs secondaires

Les objectifs secondaires de cette étude sont de déterminer si la réalisation sous échographie d'une ponction artérielle radiale « difficile » (artère non palpable ou deux échecs consécutifs par l'IDE) au SAU permet :

A- Une diminution du nombre d'effractions cutanées

B- Une diminution de la durée de réalisation (min)

C- Une diminution des complications immédiates (hématome, lésion d'un nerf, réaction vagale, pseudo-anévrisme)

D- Une amélioration du ressenti patient vis-à-vis de l'acte (EVA)

E- Une amélioration du ressenti du personnel soignant (EVA)

F- Une amélioration de la douleur ressentie par le patient (EVA)

G- Une diminution du nombre de cathéters utilisés

H- Une diminution du recours à une assistance additionnelle

4 Conception de la recherche

4.1 Méthodologie

4.1.1 Type d'essai et caractéristiques

Il s'agit d'un essai monocentrique, pilote, comparatif, contrôlé à 2 bras parallèles, randomisé, réalisé en ouvert.

Suite à l'inclusion d'un patient, une randomisation est faite pour l'assigner soit au groupe prise en charge de référence (« palpation »), soit au groupe prise en charge interventionnelle (« échoguidage »). La comparaison de ces deux groupes constitue le cadre méthodologique de cette recherche (figure 4.1).

Le soignant qui pratiquera la ponction artérielle radiale difficile devra être désigné avant la randomisation (afin d'éviter un biais dû à un manque de maîtrise dans l'utilisation de l'échoguidage) ce qui implique que seuls les soignants capables de réaliser la ponction avec les 2 techniques (palpation et échoguidage) pourront réaliser la ponction.

Tous les médecins participant à l'étude ont validé un diplôme en échographie et sont entrainés à la réalisation pratique de ce genre de ponctions (au moins 20 demi-journées) ; une formation sur « fantômes » (prothèse synthétique en gel de silicione servant à la formation et à l'entrainement au geste) sera également réalisée pour l'étude.

Dans le cas où la ponction liée à l'essai s'effectue après deux tentatives infructueuses de l'IDE, elle sera réalisée du même côté en respectant un temps de repos de 15 minutes.

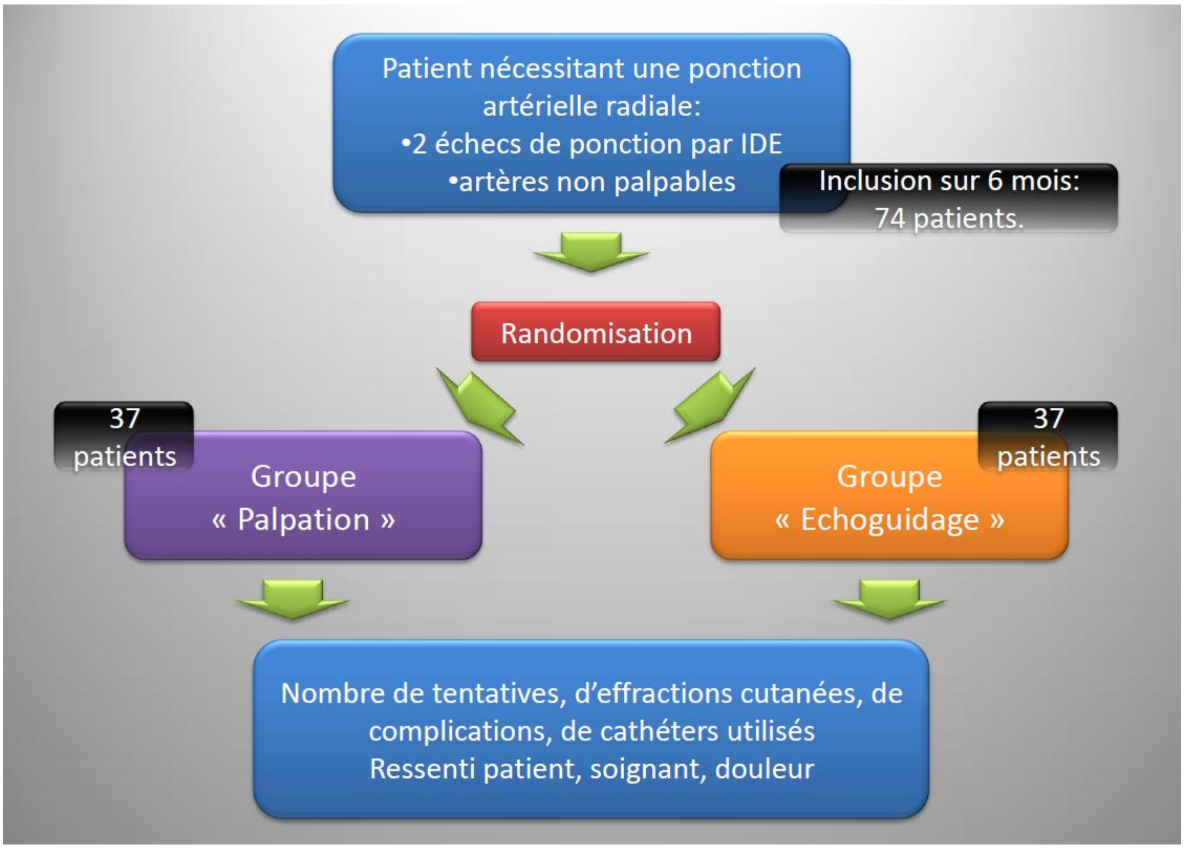

Figure 4.1 : Schéma de l'étude.

4.1.2 Critères d'évaluation

Tableau 4-1 : Les critères d'évaluation, ainsi que les unités, horizons temporels et objectifs associés.

Critère d'évaluation	Unités	Horizon temporel	Objectif
Nombre d'effractions cutanées	N	J0	A
Durée de réalisation	min	J0	B
**Une seule tentative est nécessaire**	**O/N**	**J0**	**Principal**
Complications (hématome, lésion d'un nerf, réaction vagale, pseudo-anévrisme)	O/N	J0	C
Satisfaction du patient (EVA)	0-10	J0	D
Satisfaction du soignant (EVA)	0-10	J0	E
Evaluation de la douleur ressentie par le patient (EVA)	0-10	J0	F
Nombre de cathéters utilisés	N	J0	G
Recours à une assistance additionnelle	O/N	J0	H

4.1.3 Cofacteurs

Age (an)

Sexe (F/M)

Patient dont l'artère est non palpable/patient ayant subi deux échecs de ponction par l'IDE

Indication médicale de la ponction artérielle

Pression artérielle systolique

Pathologie vasculaire sous-jacente : O/N

Fonction du soignant effectuant la ponction

Niveau d'expertise en échographie : DIUE, DU

4.2 Les visites et examens prévus

Une seule visite d'inclusion/fin de recherche (Vinc/Vfin) est nécessaire pour la réalisation de cette étude.

Tableau 4-2 Les visites prévues, leur chronologie, et les actes, examens, prélèvements associés.

	Vinc/Vfin
Chronologie (jours, semaines, mois, ans)	J0
Actes, examens, prélèvements :	

Consultation	✖
Informations générales	✖
Présentation de la note d'information	✖
Validation des critères d'inclusion et de non-inclusion	✖
Recueil de consentement éclairé	✖
Randomisation	✖
Echographie (dans le groupe Echoguidage)	✖*
Evaluation de la douleur (EVA)	✖*
Evaluation de la satisfaction du patient (EVA)	✖*
Evaluation de la satisfaction du soignant (EVA)	✖*
Relevé des complications	✖*
Recueil des événements indésirables	✖

*Ces examens ne font pas partie de la prise en charge habituelle du patient et sont propres à la recherche.

4.2.1 Visite d'inclusion

Au cours de cette visite l'investigateur :

- Délivrera une information orale et écrite (remise de la note d'information ; voir annexe 16.1 en fin de protocole) sur la mise en place de l'étude, ses objectifs, ses contraintes et les droits du patient.
- Recueillera le consentement éclairé (voir annexe 16.2 en fin de protocole)
- Une fois le consentement signé, le sujet sera randomisé. Cette randomisation est accessible via une application WEB mise à disposition par le service de Biostatistique, Epidémiologie clinique, Santé Publique, & Information Médicale (BESPIM) du CHU de Nîmes.
- Procèdera à la ponction artérielle radiale avec ou sans échographie selon le bras de randomisation
- Après ponction, évaluera la douleur ressentie par le patient (EVA)
- Evaluera la satisfaction du patient (EVA)
- Evaluera la satisfaction du soignant (EVA)

4.2.1.1 Procédures mises en œuvre pour informer, et signature du consentement libre et éclairé du sujet avant toute procédure de l'étude:

4.2.1.1.1 Procédures mises en œuvre pour informer les majeurs

Les patient(e)s seront recruté(e)s dans le service des Urgences. Lorsqu'un(e) patient(e) répond aux critères de sélection, il (elle) sera informé(e) par l'investigateur de l'existence de

l'étude. Au cours de cet entretien, les objectifs ainsi que les contraintes liées à la réalisation de l'étude et ses droits seront explicitées au patient qui pourra poser toutes les questions qu'il souhaite.

Une fois que cette information aura été donnée et que l'investigateur sera convaincu que le (la) patient(e) comprend les implications de sa participation à l'étude, le (la) patient(e) sera libre d'accepter ou de refuser de participer à l'étude d'où il (elle) pourra se retirer à tout moment et ce quelle qu'en soit la raison, et sans encourir aucune responsabilité. Quel qu'en soit le motif, le refus de participer n'aura aucune conséquence sur la qualité des soins que continuera de donner l'investigateur ni sur la qualité des relations médecin-malade.

Conformément à l'article R.5120 du code de la santé publique, les investigateurs ainsi que toutes les personnes appelées à collaborer à l'étude sont tenus au secret professionnel en ce qui concerne notamment la nature de l'étude, les personnes qui s'y prêtent et les résultats obtenus. L'investigateur s'assurera que l'anonymat des patients sera respecté. Sur l'ensemble des documents, les patients seront identifiés par un code numérique.

La signature du consentement libre et éclairé du sujet sera recueillie pendant la visite d'inclusion. Il sera laissé au patient un délai de réflexion de quelques minutes entre la présentation de la note d'information et la signature du consentement.

4.2.1.1.2 Procédures mises en œuvre pour informer les mineures et leurs parents/représentants légaux

Les mineurs sont exclus de cette recherche. Il n'y a donc pas de dispositions spécifiques pour l'inclusion de ces personnes en ce qui concerne le consentement libre et éclairé.

4.2.1.1.3 Procédures mises en œuvre pour informer les patients dans une situation d'urgence

Il n'y aura aucune situation d'urgence, car il s'agit d'une intervention programmée. Il n'y a donc pas de dispositions spécifiques pour les situations d'urgence en ce qui concerne le consentement libre et éclairé.

4.2.1.1.4 Procédures mises en œuvre pour informer les patients sous tutelle ou sous curatelle et leurs personnes de confiance/responsables légaux

Les personnes sous tutelle ou sous curatelle sont exclues de cette recherche. Il n'y a donc pas de dispositions spécifiques pour l'inclusion de ces personnes en ce qui concerne le consentement libre et éclairé.

4.2.1.1.5 Procédures mises en œuvre pour informer les patients sous sauvegarde de justice

Les personnes sous sauvegarde de justice sont exclues de cette recherche. Il n'y a donc pas de dispositions spécifiques pour l'inclusion de ces personnes en ce qui concerne le consentement libre et éclairé.

4.2.1.2 L'interdiction pour les personnes de participer simultanément à une autre recherche et la période d'exclusion prévue à l'issue de la recherche

Les patients ayant participé à une étude dans les 3 mois précédant leur inclusion dans ce protocole sont exclus dans le but d'éliminer toute interférence (connue ou non) qui pourrait conduire à sur ou sous estimer les potentielles modifications dans l'appréciation de critères d'évaluation au cours de l'étude et donc d'en fausser les conclusions. Les patients inclus ont interdiction de participer simultanément à une autre recherche interventionnelle pour les mêmes raisons.

Il n'y aura pas de période d'exclusion à la fin de l'essai.

4.2.1.3 Indemnisation des participants

Il n'y aura aucune indemnisation des patientes participant à l'étude.

4.3 Description des paramètres d'évaluation et des méthodes pour mesurer, recueillir et analyser ces paramètres

4.3.1 Les observations cliniques

Les observations cliniques seront notées dans le cahier d'observation au fur et à mesure que l'étude se déroule.

4.3.2 Les échelles visuelles analogiques

L'échelle EVA (voir section 16.3 en fin de protocole) sera présentée aux patients, qui marqueront le niveau de douleur ressentie lors de l'évaluation directement sur l'échelle graduée. Une échelle identique sera utilisée afin d'évaluer la satisfaction du patient ou du soignant concernant la réalisation de l'acte.

4.3.3 Les ponctions artérielles

Dans le cas où la ponction liée à l'essai s'effectue après deux tentatives infructueuses de l'IDE, elle sera réalisée du même côté en respectant un temps de repos de 15 minutes.

4.3.3.1 Ponction artérielle non échoguidée dite standard (groupe « palpation »)

La ponction artérielle dite standard s'effectue selon l'algorithme défini comme suit :

- Désinfection cutanée par antiseptique local
- Repérage de l'artère radiale par palpation
- Introduction de l'aiguille positionnée à 70° en regard de l'artère palpée
- Vérification de la montée de sang artériel dans la seringue.

Pour la mesure de la durée de l'acte, le chronomètre sera activé dès la première palpation de l'artère et stoppé dès la montée de sang dans la seringue.

Le prélèvement sera uniquement réalisé par des médecins thésés, praticiens habituels des Urgences.

4.3.3.2 Ponction artérielle échoguidée

La ponction artérielle avec échoguidage s'effectue comme suit :

- Désinfection cutanée par antiseptique local

- Application de gel stérile de type K-Y (ETHICON SAS)

- Repérage de l'artère par sonde vasculaire de type 9L sur appareil ***Philips Vivid S6***

-Centrage de l'artère au centre de l'écran

-Introduction de la seringue à 70° en regard de la sonde, en plein centre de celle-ci

- Vérification de la montée de sang artériel dans la seringue.

Pour la mesure de la durée de l'acte, le chronomètre sera activé dès la première étape de désinfection et stoppé dès la montée de sang dans la seringue.

4.3.4 Les critères extraits des la base de données

Les critères composites fondés sur des observations cliniques n'apparaissent pas nécessairement dans le cahier d'observation, mais sont extraits de la base de données finale. Par exemple, le poids et la taille du patients seront notés dans le cahier d'observation, mais l'IMC (= kg/m^2) sera calculé via l'informatique.

4.4 Identification de toutes les données à recueillir directement dans les cahiers d'observation

Les données à recueillir directement dans les cahiers d'observation comporte les données minimums nécessaires pour analyser :

- les critères de jugement décrits en section 4.1.2
- les cofacteurs décrits en section 4.1.3
- les variables décrites dans les descriptions des visites (section 4.2.1)
- les critères d'inclusion et de non-inclusion décrits en section 5
- les complications et événements indésirables liés à l'étude

5 Sélection et exclusion des personnes de la recherche

5.1 Description de la population à étudier

La population de l'étude est constituée de patients se présentant au Service d'Accueil des Urgences (SAU) du CHU de Nîmes et nécessitant la réalisation d'une ponction artérielle radiale à but diagnostique, pronostique ou thérapeutique.

5.2 Critères d'inclusion des personnes qui se prêtent à la recherche

Critères d'inclusion généraux :

- Le patient doit avoir donné son consentement libre et éclairé et signé le consentement

- Le patient doit être affilié ou bénéficiaire d'un régime d'assurance maladie
- Les femmes et les hommes sont inclus(es)
- Le patient est âgé d'au moins 18 ans

Critères d'inclusion concernant la population cible :

- L'état de santé du patient nécessite la réalisation d'une ponction artérielle sanguine radiale ET ses artères radiales sont non palpables ou il y a eu deux échecs préalables de ponction par l'IDE.

5.3 Critères de non-inclusion des personnes qui se prêtent à la recherche

Critères de non-inclusion généraux :

- Le sujet participe à une autre étude
- Le sujet est en période d'exclusion déterminée par une étude précédente
- Le sujet est sous sauvegarde de justice, sous tutelle ou sous curatelle
- Le sujet refuse de signer le consentement
- Il s'avère impossible de donner au sujet des informations éclairées

Critères de non-inclusion concernant les maladies ou conditions associé(e)s interférent(e)s :

- La patiente est enceinte, parturiente, ou elle allaite
- La ponction doit être réalisée sur l'artère fémorale, humérale ou axillaire.
- Le patient est en arrêt cardio-respiratoire
- Le patient présente des troubles circulatoires connus, une ischémie, une infection locale
- Le patient présente des troubles de la coagulation ou de l'hémostase sévère (hémophilie, hypoprothrombinémie, plaquettes < 50000)
- Le patient est amputé d'un membre supérieur ou a son bras plâtré.
- Le patient présente une fistule artérioveineuse ou une malformation artério-veineuse.
- Le patient présente des allergies connues au méthyl ou propylbenzoate, au propylène glycol ou au gluconate de chlorexhidine.

5.4 Durée prévue de participation des personnes, et description de la chronologie et de la durée de toutes les périodes de la recherche, y compris le suivi

5.4.1 Durée prévue de participation d'un sujet

La participation du sujet à l'étude consiste en une visite unique dont la durée n'excédera pas 2 heures.

5.4.2 Durée prévue de la recherche

Le planning prévisionnel de l'étude prévoit 30 mois d'inclusion, 6 mois de gestion des données, analyse statistique et rédaction du rapport d'étude (voir Figure 4-2).

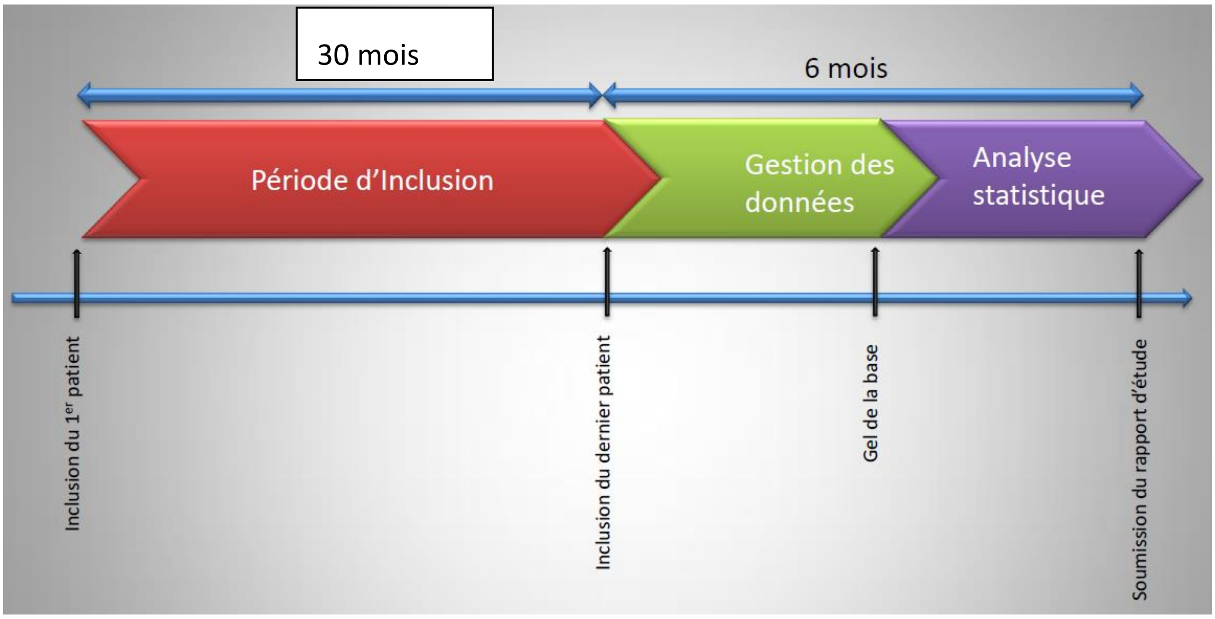

Figure 4.2 : Schématisation des différentes phases de l'étude.

Tableau 5-1 : Calendrier Prévisionnel

Jalon	Date
Demandes CPP, ANSM	Avril 2013
Début des inclusions	Février 2014
Fin des inclusions	Aout 2015
Fin de la recherche + fin des analyses statistiques et rédaction rapport	Février 2016

5.4.3 Fin de la recherche

La fin de la partie clinique de cette recherche correspond à la date de la dernière visite de la dernière personne qui se prête à la recherche.

La fiche de fin d'essai sera complétée pour tout patient ayant terminé normalement l'étude ou étant sorti prématurément.

Après la fin d'essai, le suivi habituel sera effectué par l'investigateur, qui décidera de la fréquence des visites de suivi.

La fin de la recherche correspond à la date du gel de la base de données (voir Figure 4-2).

5.5 Description des règles d'arrêt définitif ou temporaire

5.5.1 De la participation d'une personne à la recherche

La sortie du protocole se fera

- soit par décision délibérée du sujet (la participation d'un patient à la recherche s'arrêtera en cas de retrait du consentement).
- soit par nécessité, après décision de l'investigateur, dans les cas suivants:
    - Non-respect des conditions de l'étude (mauvaise compliance au traitement éventuellement prescrit, prise intercurrente de médicaments interdits, …)
    - Apparition d'un effet indésirable (grave ou non grave)

La fiche de fin d'essai sera complétée pour tout patient étant sorti prématurément de l'étude.

Le suivi habituel sera effectué par l'investigateur, qui décidera de la fréquence des visites de suivi.

5.5.2 D'une partie ou de la totalité de la recherche

La recherche pourra s'arrêter de façon temporaire ou définitive :

- Par décision de l'investigateur principal
- En cas de connaissance de données compromettant la réalisation de l'étude pour raison de sécurité des patients
- En cas de publication de données scientifiques nouvelles remettant en cause la recherche
- En cas d'évènement indésirable jugé sévère et pouvant mettre en jeu la santé des patients non clairement établi comme étant sans rapport avec la recherche.

5.6 Modalités de surveillance des personnes

La surveillance des complications et des événements indésirables est programmée selon le Tableau 4-2.

Tout événement d'urgence sera pris en charge par l'investigateur.

Tout patient, ayant subi un événement indésirable (ou non), ayant entraîné (ou non) son exclusion de la recherche sera suivi par le médecin investigateur jusqu'à la complète résolution de cet événement.

6 Traitement utilisé chez les personnes qui se prêtent à la recherche

6.1 Description de tout dispositif médical ou dispositif médical de diagnostic in vitro ou médicament qui est utilisé pour les besoins de la recherche y compris pour la période de suivi

La réalisation de l'échographie, nécessite l'utilisation :

- d'un échographe : l'appareil disponible au SAU qui sera utilisé dans le cadre de l'étude est un ***Philips Vivid S6***
- d'un gel stérile de type K-Y dont la composition à base de substance hydrosoluble ne présente pas de risques en cas de passage dans la circulation systémique (gel utilisé également pour les sondages urinaire ou les cytoponctions-biopsie percutanées du foie ou de la thyroïde).
- de matériel de prélèvement artériel composé d'une aiguille 26 Gauge et d'une seringue de 2 cc.

6.2 Médicaments et traitements autorisés et interdits dans le cadre du protocole, y compris les médicaments de secours

Il n'y a pas de médicaments proscrits dans le cadre de ce protocole.

7 Faisabilité

7.1 Gestion des patients

7.1.1 Nombre prévu de personnes à inclure dans la recherche

7.1.1.1 Justification statistique

Les données concernant les ponctions artérielles n'existent pas, à notre connaissance, dans la littérature. Mais on peut considérer le placement de cathéters artériels radiaux comme exemple de procédure comparable. Ainsi, selon l'étude de Shiver et al (2006), nous pouvons nous attendre à un taux de succès de ponction à la première tentative de 50% dans le groupe « sans échoguidage » et un taux de succès de ponction à la première tentative de 87% dans le groupe « avec échoguidage. »

Pour mettre en évidence une différence de taux de succès de ponction à la première tentative de 37% entre les deux groupes avec un risque alpha bilatéral de 5% et une puissance de 90%, le nombre de sujets à inclure au total est de 62 patients. Dans un esprit conservateur, nous augmenterons ce chiffre de 20%, ce qui nous donne un total de 74 patients soit 37 patients par groupe.

7.1.1.2 Estimation de la capacité de recrutement

La gazométrie artérielle n'étant pas cotée aux Urgences générales, nous nous sommes basés sur le potentiel de recrutement observé lors de la première étude : en 2 mois, 74 patients ont été recruté (les motifs de ponction observés sont schématisés dans la figure 1 ci-dessous).

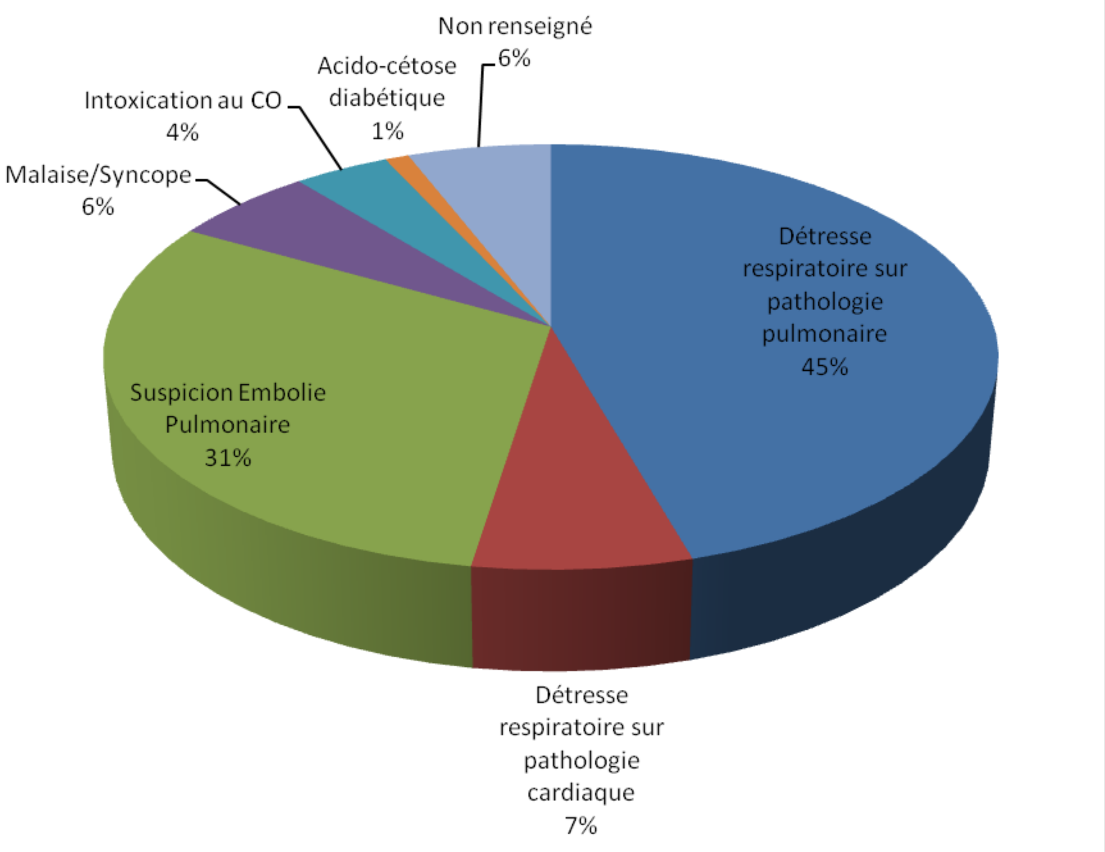

Figure1: **Motif de ponction artérielle au SAU**

7.1.2 Recrutement des patients

7.1.2.1 Lieu de recrutement

Les patients seront recrutés lors de leur admission au Service d'Accueil des Urgences.

7.1.2.2 Facteurs pouvant altérer la qualité de représentation de la population expérimentale.

A priori, la méthode de recrutement fournira une population expérimentale représentative de la population cible.

Toutefois les raisons d'un éventuel refus de participer à l'étude seront recueillies. Le taux de refus de participation sera comparé entre les patients ayant eu 2 échecs et les patients avec artère non palpable.

7.1.3 Circuit des patients

Le circuit des patients correspond à celui de la prise en charge habituelle. Dans le bras interventionnel « Echoguidage », les patients bénéficieront d'une aide à la ponction par échographie qui sera réalisée au sein du SAU.

7.1.4 Transports des patients

Cette recherche n'occasionne pas de déplacements supplémentaires par rapport à la prise en charge habituelle des patients. Il n'est donc pas nécessaire de fournir des transports spécifiques à cette étude.

7.1.5 Informations supplémentaires pour les patients

Sans objet.

7.2 Gestion médico-technique

Le circuit de patients aura lieu entièrement au sein du service des Urgences. Aucun circuit pharmaceutique n'est nécessaire à cette recherche ; les services transversaux ne seront pas sollicités pour les besoins de cette recherche.

7.2.1 Circuit pharmaceutique

Les traitements autorisés correspondent à la prise en charge habituelle du patient et ne sont pas propres à la recherche. De ce fait, la fourniture des médicaments nécessaires relève du circuit d'approvisionnement et de dispensation propre au service.

7.2.2 Analyses biologiques

Sans objet.

7.2.3 Imagerie

Dans le cas de l'utilisation de l'échographie, après une désinfection cutanée par solution hydro alcoolique antiseptique, nous appliquerons sur la zone stérile un gel lubrifiant de type K. Ce gel est stérile, sans graisse, soluble dans l'eau et non irritant. Il convient pour les explorations cytoscopiques comme les ponctions biopsie du foie ou la ponction biopsie rénale puisqu'il est aussi utilisé dans ce cadre là avec des aiguilles de diamètre plus large sans qu'aucun évènement indésirable ne soit rapporté. Les seules contre-indications sont les allergies connues au méthyl ou propylbenzoate ou au propylène glycol, ou au gluconate de chlorexhidine. En conséquence, le passage systémique d'une faible quantité de gel n'est pas dangereux, sauf en cas d'allergie connue.

7.2.3.1 Identification :

Appareil d'échographie disponible dans le service ; utilisation d'une sonde vasculaire linéaire 9L.

Toutes les mesures seront effectuées avec le même appareil.

7.2.3.2 Lieux d'imagerie

SAU du CHU de Nîmes.

7.3 Randomisation et mise à l'insu

7.3.1 Le tirage au sort

La randomisation s'effectue après vérification des critères d'inclusion et de non-inclusion, et avant la première ponction artérielle radiale difficile. Le soignant qui pratiquera la ponction artérielle radiale difficile devra être désigné avant la randomisation (afin d'éviter un biais dû à la maitrise de l'utilisation de l'échoguidage) ce qui implique que seuls les soignants capables de réaliser la ponction avec les 2 techniques (palpation et échoguidage) pourront réaliser la ponction.

Une liste de randomisation sera réalisée par le méthodologiste du service de Biostatistique, Epidémiologie clinique, Santé Publique et Information Médicale (le BESPIM) du CHU de Nîmes. Un programme sous SAS (Cary, NC, USA) spécifiquement conçu assurera ce tirage au sort.

Afin de s'assurer que les 2 motifs d'inclusion (artère non palpable ou deux échecs consécutifs par l'IDE) seront équitablement répartis dans les 2 groupes, la randomisation sera stratifiée sur ce critère.

Une application WEB (Internet®) sera spécifiquement créée. Après identification de l'utilisateur, du patient (la première lettre du nom + la première lettre du prénom + année de naissance) et vérification des critères d'inclusion et de non inclusion, le type de traitement sera indiqué à l'utilisateur. L'utilisation d'une application WEB permet ainsi une accessibilité permanente et assure un haut degré de sécurité vis-à-vis de la randomisation (impossibilité de modifier l'ordre de randomisation, assignation définitive du patient à un type de traitement et numéro de randomisation).

7.3.2 Les méthodes de mise en insu

Sans objet

7.3.3 Dispositions mises en œuvre en vue du maintien de l'insu et procédures de levée de l'insu

Sans objet

7.4 Gestion du personnel

7.4.1 Formations à prévoir

Il est prévu une présentation de l'étude lors d'un staff médical dans le service des urgences.

Seuls seront habilités à participer à cette étude les praticiens ayant un DU ou un DIU validant pour l'utilisation de l'échographie, notamment dans les abords vasculaires avec une formation technique théorique et pratique concernant la technique de prélèvement artériel radial.

Il n'y a donc pas de formation supplémentaire à prévoir hormis une démonstration pour rappel sur fantômes lors de la réunion de présentation.

7.4.2 Temps d'ARC/TEC nécessaire

Un technicien d'étude clinique (TEC) assistera l'investigateur lors de l'inclusion du patient ; il recueillera les données de l'essai disponible dans le dossier patient. Ces données seront validées par l'investigateur principal.

Il tiendra à jour le classeur investigateur et sera en charge de l'archivage de l'essai.

Le temps évalué pour réaliser cette mission est de 0.2 Equivalent Temps Plein.

Un ARC monitoring délégué par le promoteur assurera le monitoring de l'étude conformément à la réglementation en vigueur.

7.4.3 Réunions à prévoir

Une réunion de mise en place de l'étude sera organisée dans le service.

8 Evaluation de la sécurité

8.1 Description des paramètres d'évaluation de la sécurité

Il s'agit du recueil des complications et des événements indésirables.

Les numéros de série de l'échographe seront notés dans les cahiers d'observation.

8.2 Méthodes et calendrier prévus pour mesurer, recueillir et analyser les paramètres d'évaluation de sécurité

Le recueil des données concernant les complications et les évènements indésirables est programmé selon le tableau 4.1.

8.3 Procédures mises en place en vue de l'enregistrement et de la notification des événements indésirables

8.3.1 Responsabilités en matière de déclarations

Selon le code de la santé publique, Art L. 1123-10 :

« Les événements et les effets indésirables définis pour chaque type de recherche sont notifiés respectivement par l'investigateur au promoteur et par le promoteur à l'autorité compétente mentionnée à l'article L.1123-12* ainsi qu'au comité de protection des personnes compétent. Dans ce cas, le comité s'assure, si nécessaire, que les personnes participant à la recherche ont été informées des effets indésirables et qu'elles confirment leur consentement. »

« Lorsqu'un fait nouveau intéressant la recherche ou le produit faisant l'objet de la recherche est susceptible de porter atteinte à la sécurité des personnes qui s'y prêtent, le promoteur et l'investigateur prennent les mesures de sécurité urgentes appropriées. Le

promoteur informe sans délai l'autorité compétente et le comité de protection des personnes de ces faits nouveaux et, le cas échéant, des mesures prises. »

*ANSM : Agence nationale de sécurité du médicament et des produits de santé.

8.3.2 Définitions

Evénement indésirable : toute manifestation nocive survenant chez une personne qui se prête à une recherche biomédicale, que cette manifestation soit liée ou non à la recherche ou au produit sur lequel porte cette recherche.

Effet indésirable : tout événement indésirable lié à la recherche ou au produit sur lequel porte cette recherche est un effet indésirable.

Evénement/effet indésirable grave : tout événement/effet indésirable qui

- entraîne la mort ou met en danger la vie de la personne qui se prête à la recherche
- nécessite une hospitalisation ou la prolongation de l'hospitalisation
- provoque une incapacité ou un handicap important ou durable
- se traduit par une anomalie ou une malformation congénitale
- est médicalement significatif *

*Définition ICH (International Conference of Harmonization) applicable en France

Effet indésirable inattendu : Tout effet indésirable dont la nature, la sévérité ou l'évolution ne concorde pas avec les informations relatives aux produits, actes pratiqués et méthodes utilisées au cours de la recherche.

Fait nouveau : Tout fait intéressant la recherche ou le médicament faisant l'objet de la recherche et susceptible de porter atteinte à la sécurité des personnes qui s'y prêtent, qui conduit le promoteur et l'investigateur à prendre des mesures de sécurité urgentes appropriées.

8.3.3 Description des événements indésirables attendus

Dans le cadre de la présente étude les événements indésirables décrits ci-dessous sont considérés comme attendus :

8.3.3.1 Liés aux produits /actes pratiqués /méthodes utilisés au cours de la recherche

Les risques associés avec une ponction artérielle avec échoguidage sont les mêmes que pour une ponction sans échoguidage :

- Risques d'effractions cutanées,
- Douleur
- Hématomes
- Pseudo-anévrisme
- Infection

- Lésion d'un nerf
- Réaction vagale

L'échographie est réalisée après une désinfection cutanée par solution hydro alcoolique antiseptique puis l'application sur la zone stérile le gel K-Y (fiche technique en annexe 16.4) : pas de risque de contamination toxique ou infectieuse

Les effets indésirables décrits dans les notices d'utilisation de ces produits seront considérés comme attendus

8.3.3.2 Liés à l'évolution de la maladie

Sans objet.

8.3.4 Conduite à tenir en cas d'évènement indésirable ou de fait nouveau

Tous les événements indésirables graves et non graves, attendus et inattendus doivent être enregistrés dans le cahier d'observation.

Tous les faits nouveaux doivent être enregistrés dans le cahier d'observation.

8.3.4.1 Notification des événements/effets indésirable au Promoteur :

L'investigateur informe la cellule vigilance de la DRCI du CHU de Nîmes de toute survenue d'un événement indésirable grave (EIG) attendu ou inattendu, ainsi que de tout fait nouveau susceptible de porter atteinte à la sécurité des personnes se prêtant à la recherche, dès qu'il en a connaissance. Les notifications d'événements indésirables graves attendus et inattendus se font en complétant le formulaire de déclaration d'EIG et en l'adressant par fax ou par mail à l'adresse suivante:

Direction de la Recherche Clinique et de l'Innovation/ Cellule vigilance

CHU de Nîmes – Bâtiment polyvalent
Place du professeur Debré
30029 Nîmes Cedex 9

Tél. : 04 66 68 67 88
Fax : 04 66 68 34 33

Mail: vigilec@chu-nimes.fr

L'original de ce formulaire ainsi que la confirmation d'envoi sont conservés dans le dossier patient /cahier d'observation.

8.3.4.2 **Déclaration des événements indésirable graves aux autorités de santé** :

Elle est assurée par la cellule vigilance de la DRCI du CHU de Nîmes. Conformément aux articles L.1123-10, R.1123-42 et R.1123-48 du code de la santé publique, toute suspicion d'effet indésirable grave inattendu et/ou tout fait nouveau est déclaré par le promoteur CHU de Nîmes à l'autorité compétente, au CPP concerné et (le cas échéant) aux autres investigateurs dans les délais légaux.

8.4 Modalités et durée du suivi des personnes suite à la survenue d'événements indésirables

Les événements indésirables devront être suivi jusqu'à leur complète résolution.

Les informations complémentaires relatives au cas déclaré seront transmises à la cellule vigilance de la DRCI du CHU de Nîmes par fax ou mail, en complétant le formulaire de suivi de déclaration.

L'original de ce formulaire ainsi que la confirmation d'envoi seront conservés dans le dossier patient /cahier d'observation.

8.5 Comité de surveillance

Il n'est pas prévu de constituer un comité de surveillance indépendant.

9 Statistiques

9.1 Description des méthodes statistiques prévues, y compris du calendrier des analyses intermédiaires prévues

9.1.1 Description de la population incluse et des principaux paramètres étudiés

Une première analyse des données permettra la description de la population totale et par groupe. La normalité de la distribution des variables quantitatives sera explorée à l'aide du test de normalité de Shapiro-Wilks, ainsi que des coefficients de Kurtosis et de Skewness.

Les résultats statistiques seront présentés sous forme de moyennes ± écarts-types pour les variables quantitatives dont la distribution est gaussienne, moyennes et intervalles de confiance à 95% anti-transformés pour les variables dont la distribution est gaussienne après une transformation, et médianes et intervalles interquartiles pour les autres variables. Pour les variables qualitatives, les effectifs et les pourcentages associés seront présentés.

9.1.2 Analyses statistiques

L'analyse statistique sera conduite par le BESPIM du CHU de Nîmes sous SAS (SAS Institute, Cary, NC, USA) version 9 ou R 2.9.2 (R Development Core Team (2009). R Foundation for Statistical Computing, Vienna, Austria).

9.1.2.1 Vérification de la comparabilité initiale des groupes

Les variables qualitatives seront comparées par un test du Chi-2. A défaut, si les conditions de réalisation de ce test ne sont pas respectées, le test exact de Fisher sera utilisé.

Les variables quantitatives seront comparées entre les deux groupes :

- en cas de variables gaussiennes, par un test t de student ou une analyse de variance
- en cas de variables non-gaussiennes, par un test de Mann-Withney- Wilcoxon

9.1.2.2 Analyse du critère de jugement principal

Le taux de réussite à la première tentative sera comparé entre les deux groupes par un test de chi2 ou un test exact de Fisher. Le risque relatif de réussite à la première tentative dans le groupe échoguidage par rapport au groupe de référence sera estimé ainsi que son intervalle de confiance à 95%. Le nombre de sujets à traiter avec la technique sous échoguidage pour gagner un succès à la première tentative sera estimé ainsi que son intervalle de confiance à 95%.

9.1.2.3 Analyse des critères de jugement secondaires

Les critères de jugements secondaires quantitatifs (nombre d'effractions cutanées, durée de réalisation, EVA de satisfaction du patient, EVA de satisfaction du soignant, EVA douleur, nombre de cathéters utilisés) seront comparés entre les 2 groupes par un test de Student ou un test de Mann Whitney-Wilcoxon.

Les critères de jugement secondaires qualitatifs (présence de complications, recours à une assistance additionnelle) seront comparés entre les 2 groupes par un test du Chi-2 ou un test exact de Fisher.

9.2 Degré de signification prévu

Une différence sera considérée comme statistiquement significative lorsque le degré de signification du test sera inférieur ou égal à 0,05.

9.3 Critères statistiques d'arrêt de la recherche

Sans objet.

9.4 Méthode de prise en compte des données manquantes, inutilisées ou non valides ;

Pour le critère de jugement principal, il est très peu probable que nous ayons des données manquantes.

Pour les critères de jugement secondaires nous ne prévoyons pas de méthode de remplacement des données manquantes.

9.5 Choix des personnes à inclure dans les analyses

Tout patient randomisé dans l'étude sera également inclus dans les analyses qui seront réalisées en intention de traiter. Si un patient est inclus et randomisé mais si aucune tentative de ponction difficile n'est réalisée (ex : décès avant ponction), il sera exclu de l'analyse.

9.6 Gestion des modifications apportées au plan statistique initial

La décision de modifier les méthodes statistiques prévues dans ce protocole est prise par le méthodologiste indiqué en page 1.

Un changement de la méthode statistique prévue pour l'analyse du critère principal doit faire l'objet d'un amendement du protocole.

Tout changement des méthodes statistiques prévues pour l'analyse des critères secondaires sera signalé dans le rapport d'étude.

10 Droit d'accès aux données et documents source

10.1 Droit d'accès du promoteur et de l'autorité compétente aux données et documents source

Les investigateurs acceptent de se conformer aux exigences du promoteur et de l'Autorité Compétente en ce qui concerne un audit ou une inspection de l'étude.

L'audit pourra s'appliquer à tous les stades de l'étude, du développement du protocole à la publication des résultats et au classement des données utilisées ou produites dans le cadre de l'étude.

10.2 Mention de la soumission à la CNIL

Cette étude entre dans le cadre de la « Méthodologie de référence » MR-001 en application des dispositions de l'article 54 alinéa 5 de la loi du 6 Janvier 1978 modifié relative à l'informatique, aux fichiers et aux libertés.

Le CHU de Nîmes, promoteur de l'étude, s'est engagé à respecter cette méthodologie de référence et a reçu, en date du 9 mai 2006 le récépissé de déclaration de conformité MR 001.

11 Contrôle et assurance de la qualité

11.1 Monitorage & contrôle de qualité

Un assistant de recherche clinique, délégué par le promoteur (ARC promoteur), visitera de façon régulière chaque centre de l'étude lors de la mise en place de l'essai, une ou plusieurs fois en cours d'étude selon le rythme des inclusions et en fin d'étude.

Ces visites ont pour objet :

- de contrôler le respect du protocole,
- de vérifier les consentements éclairés,
- de vérifier la notification des Evènements Indésirables Graves,
- de suivre la traçabilité des dispositifs médicaux de l'étude (visite à la pharmacie, stockage et comptabilité des médicaments),
- d'assurer le contrôle de qualité : confronter les données du cahier d'observation avec les documents source du centre.

Les personnes chargées du contrôle de qualité d'une recherche biomédicale et dûment mandatées à cet effet par le promoteur ont accès, sous réserve de l'accord des personnes concernées, aux données individuelles strictement nécessaires à ce contrôle ; elles sont soumises au secret professionnel.

Toute visite fera l'objet d'un rapport de monitorage par compte-rendu écrit (traçabilité des visites).

11.2 Audit et inspection

Les investigateurs acceptent de se conformer aux exigences du promoteur et de l'Autorité Compétente en ce qui concerne un audit ou une inspection de l'étude.

L'audit pourra s'appliquer à tous les stades de l'étude, du développement du protocole à la publication des résultats et au classement des données utilisées ou produites dans le cadre de l'étude.

12 Considérations éthiques

La recherche est conduite conformément au présent protocole. Hormis dans les situations d'urgences nécessitant la mise en place d'actes thérapeutiques précis, l'(es) investigateurs(s) s'engage(nt) à respecter le protocole en tous points en particulier en ce qui concerne le recueil du consentement et la notification et le suivi des événements indésirables graves.

Le protocole sera soumis au Comité Protection des Personnes Sud Méditerranée III de Nîmes conformément à la réglementation en vigueur.

Une lettre d'information sera présentée au patient, lui précisant : le but, les objectifs et le déroulement de l'étude conformément à la réglementation, ainsi que leurs droits de refuser de participer à l'étude ou de la quitter à tout moment.

Le consentement du patient sera recherché et obtenu avant l'entrée de celui-ci dans l'étude. Un exemplaire du consentement signé lui sera remis, un exemplaire sera conservé par l'investigateur, le dernier exemplaire sera conservé par le promoteur.

Les personnes mineures, ou les personnes majeures faisant l'objet d'une mesure de protection légale ne pourront être incluses.

Il n'y aura pas de situation d'urgence dans la mesure où il s'agit d'une intervention programmée.

12.1 Obligation de l'investigateur

L'investigateur s'engage à ce que cette étude soit réalisée en conformité avec la loi n° 2004-806 du 9 août 2004 relative à la politique de santé publique et à ses décrets d'application, la déclaration d'Helsinki, les Bonnes Pratiques Cliniques.

Toutes les données, tous les documents et rapports pourront faire l'objet d'audits et d'inspections réglementaires sans que puisse être opposé le secret médical.

Toutes les informations recueillies sont confidentielles et ne pourront être divulguées. L'investigateur s'assurera que l'anonymat de chaque patient participant à l'étude est garanti. Aucune information permettant l'identification des personnes ne sera communiquée à des tiers autres que ceux, représentant du promoteur et du Ministère de la Santé, réglementairement habilités à détenir cette information (et qui sont tenus au secret professionnel).

12.1.1 La possibilité pour l'investigateur de réserver certaines informations

Il existe la possibilité pour l'investigateur, à titre exceptionnel, dans le respect de sa confiance, de réserver certaines informations liées au diagnostic (lorsque dans l'intérêt d'une personne malade le diagnostic de sa maladie n'a pu lui être révélé).

12.2 Obligation du promoteur

Conformément à la loi n° 2004-806 du 9 août 2004 relative à la politique de santé publique et à ses décrets d'application, le promoteur s'engage à réaliser l'ensemble des opérations qui lui incombent :

Assurance : Le CHU de Nîmes, promoteur de l'étude, souscrit pour toute la durée de l'étude une assurance garantissant sa propre responsabilité civile ainsi que celle de tout intervenant impliqué dans la réalisation de l'essai, indépendamment de la nature des liens existant entre les intervenants et le promoteur.

Autorité Compétente : Le promoteur sollicitera l'autorisation de l'ANSM. La recherche ne sera mise en œuvre qu'après l'avis favorable du CPP et l'autorisation de l'autorité compétente.

Commission Nationale Informatique et Liberté : Les informations recueillies lors de cette étude pourront faire l'objet d'un traitement informatique. Le fichier sera réalisé en conformité avec la CNIL (Commission Nationale de l'Informatique et des Libertés).

12.2.1 Amendement au protocole

Toute modification substantielle, c'est-à-dire toute modification de nature à avoir un impact significatif sur la protection des personnes, sur les conditions de validité et sur les résultats de la recherche, sur la qualité et la sécurité des produits expérimentés, sur l'interprétation des documents scientifiques qui viennent appuyer le déroulement de la recherche ou sur les modalités de conduite de celle-ci, fait l'objet d'un amendement écrit qui est soumis au promoteur ; celui-ci doit obtenir, préalablement à sa mise en œuvre, un avis favorable du CPP et une autorisation de l'autorité compétente.

Les modifications non substantielles, c'est-à-dire celles n'ayant pas d'impact significatif sur quelque aspect de la recherche que ce soit, sont communiquées au CPP à titre d'information.

Tous les amendements au protocole doivent être portés à la connaissance de tous les investigateurs qui participent à la recherche. Les investigateurs s'engagent à en respecter le contenu.

Tout amendement qui modifie la prise en charge des patients ou les bénéfices, risques et contraintes de la recherche fait l'objet d'une nouvelle note d'information et d'un nouveau formulaire de consentement dont le recueil suit la même procédure que celle précitée.

12.3 Justification du recueil d'informations à caractère ethnique ou racial, le cas échéant.

Sans objet.

13 Traitement des données et conservation des documents et des données relatives à la recherche

13.1 Recueil et gestion des données

Le patient ne sera identifié que par un numéro d'identification unique à 2 caractères (P☐☐), et la première lettre du nom, la première lettre du prénom, et l'année de naissance.

Une liste d'identification des sujets sera conservée dans le dossier de l'investigateur.

L'investigateur s'assurera que l'anonymat de chaque personne participant à l'étude est garanti. Aucune information permettant l'identification des personnes ne sera communiquée à des tiers autres que ceux réglementairement habilités à détenir cette information (et qui sont tenus au secret professionnel).

Les informations seront recueillies pour chaque patient sur un cahier d'observation standardisé rempli par l'investigateur ou le co-investigateur. Ce cahier comprendra l'identification du sujet, les caractéristiques de la pathologie initiale et les données de suivi.

Un cahier d'observation sera tenu pour chaque sujet participant à l'étude ; l'observation et le suivi médical concernant l'étude devront être consignés dans le document source.

Toutes les données de l'étude seront transcrites dans le cahier d'observation et/ou conservées sur support informatique par les médecins investigateurs ou leur(s) délégué(s). Les cahiers d'observation seront remplis de manière lisible et indélébile au stylo à bille bleu ou noir. En cas d'erreur, les informations erronées seront barrées d'un simple trait, la donnée initiale devant rester visible, et l'information correcte sera inscrite à côté. Chaque correction sera motivée et authentifiée (datée et signée ou paraphée par l'investigateur). L'investigateur principal signera chaque cahier d'observation pour attester de son accord avec les données y figurant.

Les données seront analysées par la cellule de méthodologie et de recherche clinique du BESPIM du CHU de Nîmes.

13.2 Saisie des Données et Procédures de Data management

Saisie des données :

La saisie sera assurée par une personne désignée.

Cette saisie sera réalisée sur un e-CRF directement ou à partir de la version papier du cahier d'observation.

Seules les personnes participant au projet de recherche et identifiées auront accès au logiciel permettant cette saisie : OpenClinica.

OpenClinica est un logiciel qui permet de générer des CRFs électroniques (e-CRF), de saisir électroniquement des données, de les gérer et de les restituer. Il permet également de suivre l'état d'avancement des études en termes de saisie et de les gérer. C'est un logiciel Open Source extensible, modulaire et fondé sur des normes internationales.

OpenClinica permet:

- La saisie de données, la validation et l'annotation par les cliniciens et les attachés de recherche
- L'extraction de données, le filtrage et l'analyse par les investigateurs
- La gestion d'étude par des coordinateurs d'étude
- La surveillance, l'audit, la configuration et le reporting par des administrateurs

La saisie dans l’e-CRF est contrôlée et formatée afin d’interdire la saisie de données hors bornes ou aberrantes. En cas de modification de saisie, une traçabilité et un suivi des activités est assuré. Une signature électronique engageant la responsabilité de l’investigateur de chaque centre permettra la validation de la visite et de l’e-CRF.

Ce logiciel est hébergé sur un site internet au sein du CHU de Nîmes. L’accès à cette application est sécurisé et se fait via l’adresse https://oc.bespim.fr avec un identifiant et un mot de passe. Les données recueillies au travers de ce logiciel fait l’objet de sauvegarde quotidienne sur un réseau sécurisé. Le réseau est connecté à internet, l’accès est protégé par un pare-feu.

Les données cliniques de l’étude seront stockées sur un répertoire spécifique du serveur. Seuls les administrateurs du réseau et les personnes habilités de la cellule recherche clinique du département information médicale peuvent avoir accès à ce répertoire.

L’extraction des données pour analyse sera réalisée soit par un responsable du département du BESPIM du CHU de Nîmes soit par le « data manager » qui possédera les droits nécessaires dans l’application.

13.3 Conservation et Archivage

La clôture de l’essai incluant la fermeture des centres sera effectuée en accord avec les Bonnes Pratiques Cliniques et ICH. Les dossiers médicaux, administratif, et des cahiers d'observations seront conservés pendant toute la durée de l'étude dans le service, puis archivés pendant une durée minimum de 30 ans.

Dispositions informatiques prises pour appliquer l’engagement de la confidentialité

- Les moyens techniques sont situés dans le service du BESPIM dont l’accès est contrôlé et sécurisé.
- Les serveurs sur lesquels sont enregistrées les données sont sauvegardés quotidiennement.

Des sauvegardes sont réalisées sur des supports magnétiques de type bandes. Un roulement des bandes sur sept jours et un roulement sur un mois est effectué.

Les disques durs des serveurs sont montés « en raid » ce qui garantit une sécurité de niveau importante. Aucune bande de stockage des données n’est stockée en salle machine. Les bandes hebdomadaires de stockage sont conservées dans une armoire fermée à clé en l’absence du ou des utilisateurs habilités par le promoteur de l’étude.

Chaque mois, une bande de sauvegarde mensuelle est conservée dans un lieu géographiquement différent dont l'accès est contrôlé et fermé à clé.

14 Financement et assurance

14.1 Durée de la garantie d'assurance et modalités particulières de couverture des risques au-delà

La durée de la garantie d'assurance souscrite par le promoteur est de 1 an

14.2 Actes et traitements propres à la recherche

Dans le bras interventionnel, une échographie sera réalisée au sein du SAU.

14.3 Montant total nécessaire pour la recherche

Le montant total des dépenses allouées à cette étude s'élève à 8930.00€.

14.4 Sources de financement

L'intégralité des dépenses de cette étude sera prise en charge par le CHU de Nîmes.

15 Règles relatives à la publication

15.1 Rapport d'Etude :

L'analyse des données sera réalisée par le BESPIM, CHU de Nîmes.

Un rapport officiel daté et signé par l'investigateur sera transmis au promoteur, qui le transmettra au Comité de Protection des Personnes et à l'autorité compétente dans les 12 mois suivant la fin de l'étude.

L'investigateur reconnaît que les résultats de l'étude sont la propriété du promoteur.

15.2 Règles de Publication :

Toute communication écrite ou orale des résultats de la recherche doit recevoir l'accord préalable de l'investigateur coordonnateur et, le cas échéant, de tout comité pour la recherche.

15.2.1 Mention du CHU de Nîmes, Nîmes, France

Pour les publications dans des revues francophones, l'affiliation pour tous les auteurs de l'article sera rédigée en français, le sigle CHU étant noté sans espace et sans point.

Exemple : service de …, CHU de Nîmes, Nîmes, France

Lorsque les auteurs publient dans des revues anglophones, l'affiliation pour tous les auteurs de l'article sera rédigée en anglais, le CHU sera cité de la manière suivante : Department of…, Nîmes University Hospital, Nîmes, France

Les coordonnées de l'auteur correspondant "corresponding author" seront écrites en français pour assurer l'acheminement de courriers éventuels.

Lorsque le CHU de Nîmes est promoteur, l'affiliation des auteurs au CHU de Nîmes est obligatoire et doit figurer en tête de liste.

15.2.2 Articles et Conférences

Toute communication écrite ou orale des résultats de la recherche doit avoir le nom d'au moins un titulaire permanent du CHU de Nîmes parmi les auteurs.

Il sera tenu compte des règles internationales d'écriture et de publication définies par l'International Committee of Medical Journal Editors (ICMJE).

15.2.3 Les Communiqués de Presse et Réactif Nîmes

La publication d'un article dans un journal de rang A est signalée au Service de Communication du CHU Nîmes.

15.3 Communication des résultats aux patients

Conformément à la loi n°2002-303 du 4 mars 2002, les patients sont informés, à leur demande, des résultats globaux de la recherche.

15.4 Cession des données

La gestion des données est assurée par le BESPIM (service de Biostatistique, Epidémiologie clinique, Santé Publique, & Information Médicale (BESPIM), CHU de Nîmes, Place du Professeur Debré, 30029 Nîmes Cedex 09). Les conditions de cession de tout ou partie de la base de données de la recherche sont décidées par le promoteur de la recherche et font l'objet d'un contrat écrit.

16 Annexes

16.1 Lettre d'information destinée au patient

Lettre d'information destinée au patient

Intérêt de l'échoguidage dans la ponction artérielle sanguine radiale « difficile ».

Version n°2 du 5 mars 2013

N° RCB : **2012-A01525-38**

Promoteur de l'étude: **Centre Hospitalier Universitaire de Nîmes**
Place du Professeur Debré
30029 Nîmes Cedex 09

Présentation de l'étude

Madame, Monsieur,

Dans le cadre de votre prise en charge au Service d'Accueil des Urgences (SAU), du sang doit être prélevé par voie artérielle radiale (artère du poignet) dans un but diagnostique. Le prélèvement consiste à effectuer une prise de sang au niveau de l'artère radiale. Celle-ci peut nécessiter plusieurs tentatives, notamment si vos artères ne sont pas facilement repérables à la palpation. Nous pensons qu'un échoguidage (guidage grâce à un examen échographique) de cet acte pourrait permettre un meilleur taux de réussite dès le premier essai et en conséquence une réduction du temps nécessaire à sa réalisation et un confort accru pour vous.

C'est ce que nous nous proposons d'évaluer dans une étude clinique intitulée « Intérêt de l'échoguidage dans la ponction artérielle radiale difficile » à laquelle le Docteur GENRE-GRANDPIERRE vous propose de participer. Cette étude permettra de déterminer si l'utilisation d'un appareil d'échographie peut présenter un gain pour les patients en termes de durée de l'acte et de douleur ressentie.

Cette note est destinée à vous donner des éléments d'information sur cette étude. Si vous ne comprenez pas bien certains mots ou éléments de cette note, n'hésitez pas à demander des explications au médecin.

La signature du formulaire de consentement attestera de votre accord final pour participer à cette étude.

Les sujets inclus dans l'étude devront être affiliés ou bénéficiaires d'un régime de sécurité sociale.

Objectifs de l'étude

L'objectif principal de cette étude est de déterminer si l'utilisation au service d'accueil des urgences d'un échographe pour la réalisation d'une ponction artérielle radiale « difficile », (en raison d'une artère non palpable ou après deux échecs consécutifs) permet d'améliorer le taux de réussite à la première tentative.

L'échec est défini par une quantité de sang prélevé insuffisante, l'absence de reflux sanguin ou un prélèvement accidentel dans la veine au lieu de l'artère.

Les objectifs secondaires de cette étude seront de déterminer si l'utilisation d'un échographe permet une diminution du nombre de piqûres d'aiguille nécessaires pour la réussite du prélèvement sanguin, de la durée de réalisation, des complications immédiates (hématome, malaise…), et une amélioration de la douleur ressentie, ainsi que de la satisfaction du patient et du soignant.

Méthodologie de l'étude

Suite à l'inclusion d'un patient dans l'étude, un tirage au sort (randomisation) permet de l'assigner soit au groupe prise en charge de référence (« palpation »), soit au groupe prise en charge interventionnelle (« échoguidage »). La comparaison de ces deux groupes constitue le cadre méthodologique de cette recherche.
Les chances de tomber dans l'un ou l'autre groupe sont de 50/50.

Nombre de patients

74 patients admis au service d'accueil des urgences du CHU de Nîmes seront inclus dans l'étude.

Déroulement de l'étude et nombre de visites

Le déroulement de l'étude consiste en une visite unique qui correspond à la réalisation d'une ponction artérielle radiale ; lors de cette intervention, aucune modification des soins habituellement réalisés ne sera effectuée en dehors d'un examen échographique dans le groupe avec prise en charge interventionnelle (« échoguidage »). Au cours de cette visite il vous sera demandé d'estimer après la réalisation de l'acte, la douleur ressentie ainsi que votre satisfaction.

Contraintes et Obligations

Le volume de sang prélevé sera de 2 ml comme pour tout prélèvement pour le dosage des gaz du sang.

La seule contrainte supplémentaire sera d'évaluer votre douleur et votre satisfaction à l'aide d'une échelle visuelle analogique (sorte d'échelle graduée de 0 à 10).

Bénéfice potentiel attendu pour le participant et indemnisation

Le bénéfice attendu dans le bras interventionnel (ponction avec échoguidage) serait une augmentation du taux de réussite à la première tentative et une diminution de la durée de réalisation de l'acte. Cette hypothèse doit cependant être confirmée, raison pour laquelle nous réalisons cette étude.

L'ensemble des traitements et des investigations sera entièrement pris en charge dans le cadre de la recherche et, le cas échéant vous aurez droit au remboursement des frais exposés.

Cette étude ne donne lieu à aucune indemnité.

Risques engendrés du fait de la recherche

Les risques associés avec une ponction artérielle avec échoguidage sont les mêmes que pour une ponction sans échoguidage : douleur, hématomes, pseudo-anévrisme (lésion partielle de l'artère), infection, lésion d'un nerf, réaction vagale.

Droits et garanties des personnes

Dans le cadre de cette recherche, un traitement de vos données personnelles va être mis en œuvre afin de permettre l'analyse des résultats au regard de l'objectif de cette recherche. A cette fin, les données médicales vous concernant, identifiées par un numéro de code, seront

transmises au promoteur. Elles pourront également, dans des conditions assurant leur confidentialité, être transmises aux autorités de santé françaises.

Vous pourrez, conformément aux dispositions de la loi « Informatique et Libertés » (loi n° 2004-801 du 6 août 2004) disposer d'un droit d'accès et de rectification. Vous disposez également d'un droit d'opposition à la transmission des données couvertes par le secret professionnel susceptibles d'être utilisées dans le cadre de la recherche. Vous pouvez accéder directement ou par l'intermédiaire d'un médecin de votre choix à l'ensemble de vos données médicales en application des dispositions de l'article L 1111-7 du Code de la Santé Publique.

Ces droits s'exercent auprès du médecin responsable de l'étude.

Si vous le désirez, les résultats globaux de ce travail vous seront communiqués à sa conclusion.

Vous pourrez à tout moment bénéficier d'informations supplémentaires sur la recherche et toute nouvelle connaissance susceptible de remettre en question votre participation vous sera communiquée.

Conformément à la loi du 4 mars 2002 relative au droit des malades, vous avez le droit de vous faire accompagner d'une personne de confiance pour tout acte médical mettant en jeu votre consentement.

Votre médecin traitant pourra être averti de votre participation à ce projet, sauf si vous vous y opposez.

Données Réglementaires

Conformément aux dispositions législatives et réglementaires, le CHU de Nîmes, a souscrit sous le n° 131562 un contrat d'assurance couvrant la Responsabilité Civile du Promoteur de Recherche Biomédicale auprès de la
(18 rue Edouard Rochet – 69372 LYON Cedex 08).

Les modalités de ce protocole ont été soumises à l'examen du Comité de Protection des Personnes (CPP) Sud Méditerranée III qui a pour mission de vérifier si les conditions requises pour votre protection et le respect de vos droits sont respectées.

Ce comité a émis un avis favorable en date du ……………………

Ce protocole a reçu l'aval de l'autorité compétente (ANSM) en date du …………………….

Vous êtes totalement libre d'accepter ou de refuser de participer à cette étude. Votre médecin vous laissera le temps nécessaire pour prendre votre décision.

Si vous avez des questions, n'hésitez pas à les poser au médecin.

Nom et coordonnées de l'investigateur principal :
**Dr Romain GENRE-GRANDPIERRE**<br>**Service des Urgences**<br>**CHU de Nîmes**

Soyez assuré(e) que votre participation nous est extrêmement précieuse. Nous vous remercions par avance, Madame, Monsieur de l'aide que vous apportez ainsi à la recherche médicale.

Nom et signature de l'Investigateur : ………………………

16.2 Formulaire de Consentement Libre et Eclairé pour le(la) patient(e) participant à une recherche biomédicale

Formulaire de Consentement Libre et Eclairé pour le(la) patient(e) participant à une recherche biomédicale

Lisez avec attention les différentes parties de ce document. Ne signez ce document qu'après en avoir lu et rempli toutes les parties (fait en 3 exemplaires, 1 pour le promoteur, 1pour l'investigateur local et 1 pour le(la) patient(e)).

Intérêt de l'échoguidage dans la ponction artérielle sanguine radiale « difficile ».

Version n°1 du 26 Juillet 2012

*N° RCB : **2012-A01525-38***

Je soussigné(e) ………………………………………………………………………………, né(e) le …………………………………… accepte de participer, selon les conditions définies dans la lettre d'information, à la recherche désignée ci-dessus menée par le Dr Romain GENRE-GRANDPIERRE.

- J'atteste être affilié(e) ou bénéficiaire d'un régime d'assurance maladie.

Faculté d'opposition et retrait du consentement :

- Je comprends que ma participation à cette étude est volontaire et que je peux la refuser. Si je le désire, je serai **LIBRE A TOUT MOMENT DE L'ETUDE D'ARRETER MA PARTICIPATION** sans avoir à me justifier. J'en informerai l'investigateur principal de l'étude le Dr GENRE-GRANDPIERRE par écrit à l'adresse

suivante : Service des Urgences, CHU de Nîmes, Place du Professeur Debré, 30029 Nîmes Cedex 09.

- Je comprends que ma décision de participer à cette étude ne libère ni les chercheurs, ni l'établissement hôte de leurs obligations professionnelles et légales envers moi.
- J'ai pris connaissance de mon droit d'accès et de rectification des informations nominatives me concernant et qui sont traitées de manière automatisée. Le droit d'accès et de rectification prévu par la loi "Informatique et Liberté" s'exerce à tout moment auprès des responsables de l'étude.

Reconnaissance de l'information préalable :

- Le médecin m'a informé(e) de la nature et des buts de ce projet de recherche, ainsi que de son déroulement et des bénéfices et risques éventuels de l'étude.
- A cet égard, j'ai en ma possession une note d'information (Version N°1 du 26 Juillet 2012) que j'ai lue, comprise.
- J'ai pu poser toutes les questions voulues concernant ce projet et j'ai obtenu des réponses satisfaisantes. Je reconnais avoir disposé d'un temps de réflexion suffisant entre ces informations et le présent consentement et avoir eu, si je le souhaitais, l'opportunité d'en discuter avec mon médecin ou mes proches.
- Je reconnais en particulier que le droit de me faire assister par une personne de confiance de mon choix m'a été communiqué.
- Je reconnais avoir été informé(e) que l'étude pouvait être interrompue à tout moment sur décision du promoteur ou des autorités, que toutes les mesures seraient prises dans ce cas pour assurer ma sécurité et, le cas échéant, la poursuite de mon traitement, et que ma participation personnelle pouvait être suspendue si je ne respectais pas le protocole.
- Je sais que tout fait nouveau susceptible de remettre en cause mon consentement à ma participation dans l'étude me sera communiqué.

Acceptation des contraintes :

- Je m'engage à observer les contraintes expliquées et spécifiées dans le document d'information, à la fois pour minimiser les risques et pour la bonne fin du protocole.
- J'atteste formellement n'avoir participé à aucune recherche biomédicale depuis 3 moid et ne participer à aucune étude en cours.
- J'accepte l'interdiction qui m'a été signifiée de participer simultanément à une autre recherche.
- J'ai bien compris que le fait de dissimuler la vérité peur entraîner des conséquences préjudiciables pour ma santé. J'atteste en conséquence, avoir répondu de façon sincère à toutes les questions qui m'ont été posées, notamment celles relatives à mon état de santé et mes habitudes de vie.
- Je m'engage à respecter les règles de confidentialité s'appliquant à l'étude, telles quelles m'ont été expliquées préalablement.

Autres autorisations :

- Dans le cadre de cette étude, toutes les DONNEES ET INFORMATIONS me concernant, resteront strictement CONFIDENTIELLES. Je n'autorise leur consultation que par des personnes désignées par les investigateurs et éventuellement par un représentant des autorités de santé. Je suis informée que ces données cliniques recueillies feront l'objet d'un traitement informatisé autorisé par la Commission Nationale Informatique et Liberté.
- Conformément à la loi du 4 mars 2002, relative au droit des patients, je sais que j'ai la possibilité de me faire accompagner d'une personne de confiance pour tout acte médical mettant en jeu mon consentement.

Date et signature du patient :	Date et signature de l'investigateur :
______________________________	______________________________
______________________________	______________________________

Promoteur de l'étude :

Centre Hospitalier Universitaire de Nîmes

Place du Pr Debré

30029 Nîmes Cedex 09

16.3 Echelle Visuelle Analogique (EVA)

Instructions pour le(la) patient(e) :

Tracez une ligne verticale **(|)** passant par le point de l'échelle ci-dessous qui décrit le niveau de la douleur (de satisfaction) que vous ressentez, en considérant « 0 » comme l'absence de toute douleur (de satisfaction), et « 10 » comme une douleur intolérable (satisfaction totale).

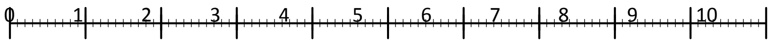

Pas de douleur — Douleur intolérable

Totalement insatisfait — Satisfaction totale

Inscrivez ci-dessous vos initiales, la date et l'heure de mesure :

Initiales du sujet : ___________

Date : _____/_____/_____

16.4 Références

Andersen, A., K. Milojevic, A. Nédélec-Jaffuel, S. Silva, A. Beltramini, R. Ruiz Almenar, C. Barbier, et J. L. Ricôme. 2009. Intérêt des gaz du sang en préhospitalier pour les patients ventilés: étude prospective. *Journal Européen des Urgences* 22: A119.

Bauman, M., D. Braude, et C. Crandall. 2009. Ultrasound-guidance vs. standard technique in difficult vascular access patients by ED technicians. *American Journal of Emergency Medicine* 27, n°. 2: 135-140.

Chabot, F., E. Gomez, A. Guillaumot, A. Kheir, et A. Chaouat. 2009. Exacerbations de bronchopneumopathie chronique obstructive= Acute exacerbations of chronic obstructive pulmonary disease. *La Presse médicale* 38, n°. 3: 485-495.

Costantino, T. G., A. K. Parikh, W. A. Satz, et J. P. Fojtik. 2005. Ultrasonography-guided peripheral intravenous access versus traditional approaches in patients with difficult intravenous access. *Annals of emergency medicine* 46, n°. 5: 456-461.

Donati, S. Y., M. Gainnier, et O. Chibane-Donati. 2005. Intoxication au Monoxyde de carbone. *EMC-Anesthésie-Réanimation* 2, n°. 1: 46-67.

Dudeck, O., Teichgraeber, U., Podrabsky, P., Lopez Haenninen, E., Soerensen, R., Ricke, J. 2004. A randomized trial assessing the value of ultrasouns-guided puncture of the femoral artery for interventional investigations. Int J Cardiovascular Imaging 20:363-368

Franklin, C. 1995. The technique of radial artery cannulation. Tips for maximizing results while minimizing the risk of complications. *The Journal of Critical Illness* 10, n°. 6 (Juin): 424-432.

Ganesh, A., R. Kaye, A. M. Cahill, W. Stern, R. Pachikara, P. R. Gallagher, et M. Watcha. 2009. Evaluation of ultrasound-guided radial artery cannulation in children. *Pediatric Critical Care Medicine* 10, n°. 1: 45.

Giner, J., P. Casan, J. Belda, H. Litvan, et J. Sanchis. 2000. Use of the anesthetic cream EMLA in arterial punction. *Revista española de anestesiologia y reanimacion* 47, n°. 2: 63.

Gratrix, A. P., J. D. Atkinson, et A. R. Bodenham. 2009. Cannulation of the impalpable section of radial artery: preliminary clinical and ultrasound observations. *European journal of anaesthesiology* 26, n°. 10: 887.

Kannan, S. 2005. Another use for ultrasound in the ICU. Anaesthesia 60: 944.

Levin, PD., Sheinin, O., Gozal, Y. 2003. Use of ultrasound guidance in the insertion of radial artery catheters. Critical Care Medicine 31, n° 2: 481-484.

Lorut, C. 2005. Embolies pulmonaires. *EMC-Cardiologie-Angéiologie* 2, n°. 4: 531-541.

Maggiore, S. M., et P. Jolliet. 2005. Quels outils et quels paramètres ventilatoires peuvent permettre d'adapter au mieux les réglages du ventilateur et quelle est leur place dans la surveillance ventilatoire au cours du Syndrome de Détresse Respiratoire Aiguë?=

Which tools and parameters can be useful for ventilatory settings and monitoring in Acute Respiratory Distress Syndrome? *Réanimation* 14, n°. 5: 359-366.

Marshall, B. E. 1964. METHOD OF ARTERIAL SAMPLING. *The Lancet* 284, n°. 7349: 40.

Martin, C. 1995. Cathétérisme artériel et mesure invasive de la pression artérielle en anesthésie-réanimation chez l'adulte= Arterial catheterisation and invasive measurement of arterial blood pressure in anaesthesia and intensive care in adults. Dans *Annales françaises d'anesthésie et de réanimation*, 14:444-453. Elsevier.

Micu, E., C. Guillot, M. Badier, S. Delpierre, J. M. Régis, et P. Roussel. 2006. Pain induced by radial artery puncture is not reduced by lidocaine-prilocaine patch. *Respiratory Medicine Extra* 2, n°. 1: 52-53.

Mizukoshi, K., Shibasaki, M., Amaya, F., Hirayama, T., Shimizu, F., Hosokawa, K., Hashimoto, S., Tanaka, Y. 2009. Ultrasound evidence of the optimal wrist position for radial artery cannulation. Can J Anesth 56: 427-431.

Nagabhushan, S., Colella, JJ., Wagner, R. 1976. Use of Doppler ultrasound in performing percutaneous cannulation of the radial artery. Crit Care Med 4:327.

Sandhu, NS., Patel, B. 2005. Use of ultrasonography as a rescue technique for failed radial artery cannulation. J Clinical Anesthesia 18: 138-141.

Sarrot-Reynauld, F., et B. Paramelle. 2003. La ponction artérielle radiale pour analyse des gaz du sang. Etude du vécu des patients. *Revue de Medecine Interne* 24, n°. 4: 484.

Scheer, Bernd, Azriel Perel, et Ulrich J Pfeiffer. 2002. Clinical review: complications and risk factors of peripheral arterial catheters used for haemodynamic monitoring in anaesthesia and intensive care medicine. *Critical Care (London, England)* 6, n°. 3 (Juin): 199-204.

Schwemmer, U., Arzet, HA., Traunter, H., Rauch, S., Roewer, N., Greim, CA. 2006. Ultrasound-guided arterial cannulation in infants improves success rate. Eur. J. Anaesthesiol. 23, 476-480.

Shiloh, AL, Eisen, LA. 2010. Ultrasound-guided arterial catheterization: a narrative review. Intensive Care Med 36: 214-221.

Shiver, S., M. Blaivas, et M. Lyon. 2006. A prospective comparison of ultrasound-guided and blindly placed radial arterial catheters. *Academic Emergency Medicine* 13, n°. 12: 1275-1279.

Spunda, R., T. Urban, J. Tosovský, J. Táborský, et M. Semrád. 2005. Postcatheterization pseudoaneurysm of the radial artery. *Rozhledy v chirurgii: m sí ník eskoslovenské chirurgické spole nosti* 84, n°. 5: 244.

Taytard, A., J. P. Daures, P. Arsac, J. L. Chirumberro, J. P. Grignet, M. Micoud, R. Poirier, P. Romand, J. M. Tartavel, et D. Touron. 2001. Prise en charge des infections respiratoires basses en médecine générale en France. *Rev Mal Respir* 18: 163-170.

Wilson, SR., Grunstein, I., Hirvela, ER., Price, DD. 2010. Ultrasound-guided radial artery catheterization and the modified Allen's test. J of Emerg Med 38, n°3, 354-358.
